# Glutaminolysis Blockade‐Empowered Bimodal Nanodepot for Spatially Complementary Sono‐Thermal Ablation via PANoptosis and STING Activation Against Large Tumors

**DOI:** 10.1002/advs.77933

**Published:** 2026-09-27

**Authors:** Yi‐Ming Liu, Juan Qin, Si‐Yuan Wu, Xue‐Zhen Zhu, Yun‐Fei Cai, Pei‐Yao Sun, Hao Yang, Xing‐Yue Wang, Shu Li, Duo Wang, Hai‐Dong Zhu

**Affiliations:** ^1^ Center of Interventional Radiology and Vascular Surgery Department of Radiology Zhongda Hospital Medical School Southeast University Nanjing China; ^2^ The Third Affiliated Hospital of Zhengzhou University Zhengzhou China

**Keywords:** cGAS‐STING pathway, glutaminolysis blockade, immunogenic PANoptosis, injectable dual‐crosslinked nanodepot, microwave‐ultrasound synergy, Spatially complementary sono‐thermal ablation

## Abstract

Microwave ablation (MWA) for large hepatocellular carcinoma (HCC) is frequently compromised by non‐uniform thermal distribution and immunosuppressive niche formation. To address these challenges, we engineered an injectable bimodal nanodepot (TCPD) via catalytic crosslinking of dopamine‐modified hyaluronic acid by microwave thermal converter cesium‐doped Prussian blue, enabling co‐delivery of glutaminase 1 inhibitor Telaglenastat and sonosensitizer Chlorin e6. Under combined MWA and ultrasound irradiation, TCPD nanodepot disrupts cellular antioxidant defenses and bioenergetic pathways through glutaminolysis‐TCA cycle blockade, metabolically sensitizing large tumors to spatially complementary sono‐thermal ablation and enabling complete treatment coverage. This multimodal intervention induces multiple subcellular stresses, including mitochondrial and endoplasmic reticulum dysfunction, genotoxicity, and NAD^+^ depletion, which converge to facilitate PANoptosome assembly and immunogenic PANoptosis while activating the cGAS‐STING pathway. Subsequent release of damage‐associated molecular patterns and immunoregulatory cytokines promotes dendritic cell maturation, M1 macrophage polarization, and T lymphocyte infiltration, ultimately reprogramming the immunosuppressive microenvironment toward an immune‑activated niche. Integration with anti‐PD‐1 therapy further reinvigorates cytotoxic T lymphocytes and establishes antitumor immune memory to suppress tumor progression and metastasis, offering a multimodal therapeutic strategy for large HCC.

## Graphical abstract

An injectable bimodal TCPD nanodepot disrupts glutaminolysis and TCA metabolism, sensitizing large tumors to spatially complementary sono‐thermal ablation for complete treatment coverage. This intervention induces mitochondrial and endoplasmic reticulum dysfunction, genotoxicity, and NAD^+^ depletion, converging to trigger PANoptosome assembly and immunogenic PANoptosis while activating the cGAS‐STING pathway, ultimately reprogramming the immunosuppressive microenvironment and enhancing the antitumor immune response.

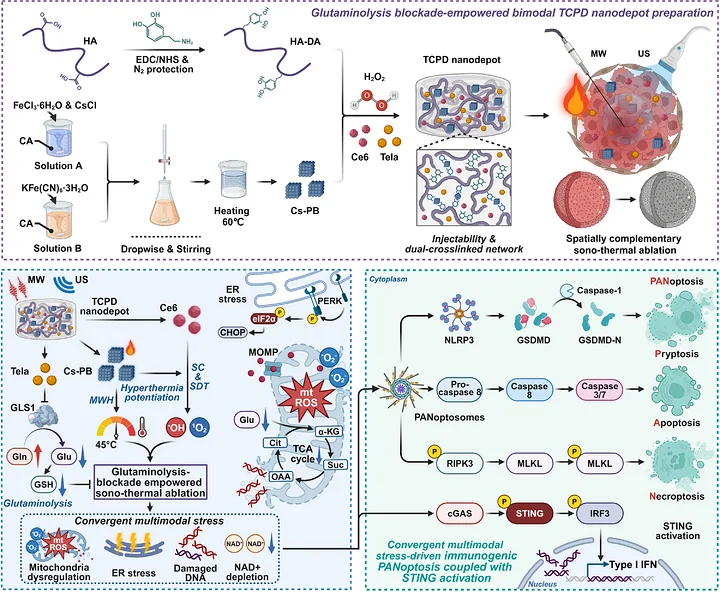

## 1 Introduction

Extending microwave ablation (MWA) to large hepatocellular carcinoma (HCC; >3 cm) remains challenging due to substantial tumor burden, pronounced intratumoral heterogeneity, and the biophysical constraints of thermal energy deposition [1, 2, 3]. Although integration of MWA with immune checkpoint inhibitors (ICIs) has been pursued to leverage ablation‐induced immune activation, achieving robust abscopal effects and durable clinical benefit in large HCC remains an elusive goal. A critical driver of this therapeutic limitation is the inherent non‐uniformity of thermal dose distribution during MWA of large HCC, which subjects residual tumor subpopulations to sublethal thermal stress, triggering profound molecular remodeling toward a more aggressive and therapy‐resistant phenotype and thereby curtailing the expected immunogenic potential [4, 5, 6]. Furthermore, these thermally stressed residual cells actively drive the local tumor microenvironment (TME) toward an immunosuppressive state by promoting recruitment of myeloid‐derived suppressor cells (MDSCs) and regulatory T cells (Tregs), while concurrently driving functional exhaustion of tumor‐infiltrating cytotoxic T lymphocytes (CTLs) [7, 8, 9]. Consequently, the post‐ablation malignant niche establishes a dual barrier of cell‑death resistance and immune suppression, which substantially undermines the efficacy of subsequent ICIs and poses a major obstacle to achieving long‐term survival.

To overcome these dual barriers, an ideal therapeutic strategy should combine efficient eradication of tumor cells by bypassing their intrinsic death resistance with generation of a systemic antitumor immune response. Consequently, research efforts have shifted toward the coordinated induction of multiplexed regulated cell death pathways to harness their synergistic effects, thereby maximizing tumor clearance and counteracting intratumoral heterogeneity [10, 11, 12, 13]. One modality central to this paradigm is PANoptosis, a highly inflammatory programmed cell death modality structurally governed by the PANoptosome, which functions as a multiprotein supramolecular complex that integrates sensors, adapters, and effectors to execute concurrent pyroptosis, apoptosis, and necroptosis [14, 15]. Upon sensing danger signals such as excessive reactive oxygen species (ROS) or cytosolic DNA, sensors including Z‐DNA binding protein 1 (ZBP1), absent in melanoma 2 (AIM2), or NLR family pyrin domain containing 3 (NLRP3) assemble the PANoptosome to simultaneously trigger gasdermin D (GSDMD)‐mediated pyroptosis, caspase‐3‐dependent apoptosis, and mixed lineage kinase domain‐like pseudokinase (MLKL)‐driven necroptosis [16, 17]. Unique therapeutic potency of PANoptosis stems from its dual capacity for integrated death execution coupled with profound immunogenicity, highlighted by massive release of pro‐inflammatory cytokines, specifically interleukin (IL)‑1β and IL‑18, alongside various damage‐associated molecular patterns (DAMPs) [18, 19, 20]. Crucially, damaged DNA released during severe mitochondrial disintegration serves as a pivotal convergent signal that not only facilitates PANoptosome assembly but also activates the stimulator of interferon genes (STING) innate immune pathway [21, 22, 23, 24]. Specifically, cytosolic accumulation of double‐stranded DNA is recognized by cyclic GMP‐AMP synthase (cGAS), initiating the STING‐dependent production of type I interferons (IFN‐α/β) [25, 26, 27]. Therefore, triggering a profound subcellular damage to drive immunogenic PANoptosis in coordination with cGAS‑STING pathway activation establishes a therapeutic cascade that promises to eradicate death‐resistant tumor populations while providing antigenic and adjuvant signals to initiate and sustain a durable cancer‐immunity cycle [23, 28].

Translating the proposed cascade of immunogenic PANoptosis coupled with STING activation into a precise therapeutic intervention presents substantial challenges, primarily because MWA alone cannot trigger this complex biological process. Sonodynamic therapy (SDT), which exploits low‐intensity ultrasound (US)‐induced cavitation and sonoluminescence to non‐invasively activate sonosensitizers for spatiotemporally controlled ROS generation, provides an effective approach to address these limitations [29, 30, 31, 32]. Superior penetration depth of US, exceeding 10 cm with minimal attenuation, enables therapeutic reach into both peripheral and central regions of deep‐seated tumors such as HCC, distinguishing SDT from light‐driven modalities constrained by superficial tissue penetration limits [33, 34, 35]. Integrating SDT with MWA within a unified sono‐thermal ablation capitalizes on their spatial complementarity, wherein peripheral sonodynamic ROS generation and central MWA‐mediated hyperthermia combine to provide comprehensive tumor coverage [36, 37]. This design leverages the existing clinical use of US for MWA guidance, converting it from a passive imaging tool into an active therapeutic modality that simultaneously performs imaging and SDT without additional equipment. Beyond this spatial synergy, these combined physical modalities induce mitochondrial oxidative injury and promote cytosolic leakage of damaged DNA, thereby facilitating immunogenic PANoptosis while activating the cGAS‐STING pathway. Nevertheless, this organelle‐level injury may be partially mitigated by metabolic plasticity and antioxidant defenses inherent to tumor cells, underscoring the need for an additional metabolic intervention to suppress cellular adaptation and enhance the therapeutic efficacy of spatially complementary sono‐thermal ablation.

Glutamine (Gln) is a conditionally essential amino acid that functions as a metabolic hub connecting carbon and nitrogen flux with redox homeostasis [38]. Critically, large HCC typically exhibits increased dependence on Gln metabolism to adapt to hypoxic and nutritional stress. Mechanistically, while this metabolic adaptation relies on Gln uptake, it is primarily executed by its derivative, glutamate (Glu), which accumulates via the upregulation of glutaminase 1 (GLS1)‐mediated glutaminolysis to establish a metabolic defense network. First, generated Glu conjugates with cysteine through the γ‐glutamyl cycle to synthesize glutathione (GSH), a primary intracellular antioxidant that neutralizes cytotoxic ROS [39, 40, 41]. Second, intracellular Glu serves as an anaplerotic substrate to replenish α‐ketoglutarate in the tricarboxylic acid (TCA) cycle, sustaining bioenergetics required for cell survival and repair under thermal and oxidative stress [42]. Third, Glu‐derived metabolic flux supports salvage pathways that maintain the nicotinamide adenine dinucleotide (NAD^+^) pool, supplying cofactors for NADPH regeneration and maintaining GSH in a reduced state [43]. Given that this metabolic defense limits the efficacy of spatially complementary sono‐thermal ablation, pharmacological inhibition of GLS1 can disrupt these adaptive pathways. Notably, depletion of the NAD^+^ pool impairs redox recycling and acts as a metabolic signal to upregulate NLR family CARD domain‐containing protein 5 (NLRC5), a sensor that nucleates a distinct branch of PANoptosome [44]. Therefore, integrating glutaminolysis blockade with spatially complementary sono‐thermal ablation may provide a sensitization strategy to enhance tumor clearance, inducing immunogenic PANoptosis and STING pathway activation driven by multiple danger signals.

**SCHEME 1:**
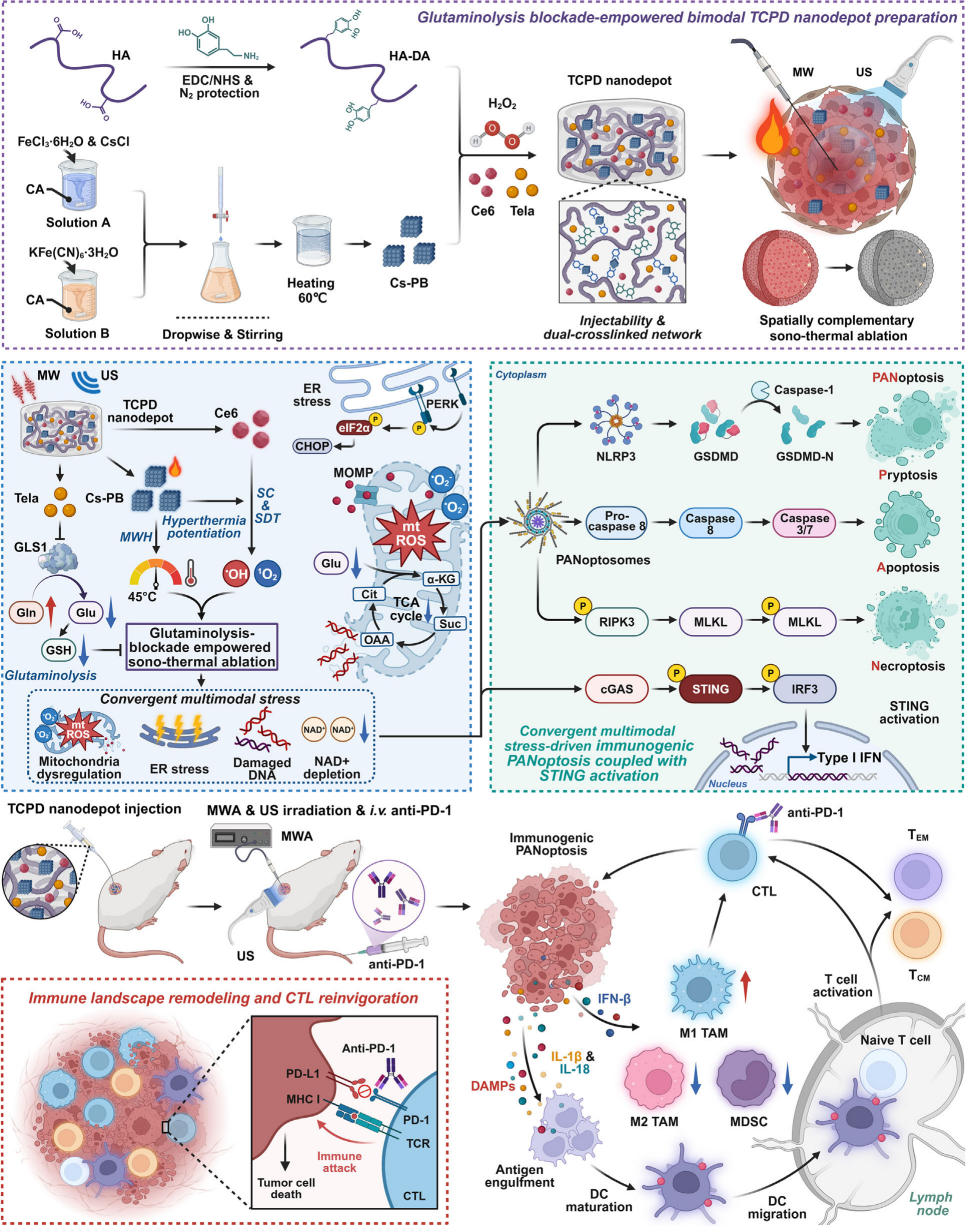
Schematic illustration of the engineered bimodal TCPD nanodepot disrupting the glutaminolysis‐TCA axis to empower spatially complementary sono‐thermal ablation. This synergistic modality subsequently orchestrates immunogenic PANoptosis and robust STING activation, driving systemic immune reprogramming for the eradication of large tumors.

In this study, we further engineered a glutaminolysis blockade‐empowered bimodal nanodepot incorporating GLS1 inhibitor Telaglenastat (Tela), sonosensitizer Chlorin e6 (Ce6), and MW thermal converter cesium‐doped Prussian blue (Cs‐PB) by leveraging a Cs‐PB‐catalyzed dopamine‐modified hyaluronic acid (HD) crosslinking strategy, designated as Tela/Ce6/Cs‐PB@HD (TCPD). Under combined MWA with US irradiation, locally administered TCPD nanodepot serves as a bimodal‐responsive reservoir, exerting spatially complementary sono ‐ thermal ablation empowered by targeted glutaminolysis‐TCA cycle blockade. Mechanistically, this coordinated intervention induces multimodal subcellular stress, including mitochondrial dysfunction, endoplasmic reticulum (ER) stress, genotoxicity, and NAD^+^ depletion. Critically, these danger signals converge to facilitate PANoptosome assembly to drive immunogenic PANoptosis, while simultaneously activating the cGAS‐STING pathway to enhance IFN‑β production. Subsequent release of DAMPs and immunoregulatory cytokines promotes dendritic cell (DC) maturation, polarizes macrophages toward an M1 phenotype, and facilitates T‑cell infiltration, ultimately reprogramming immunosuppressive TME into an immune‑activated state. Crucially, synergizing with anti‐PD‐1 blockade further reinvigorates CTLs and establishes durable antitumor immune memory to effectively suppress large tumor progression and metastasis. This integrated strategy addresses dual barriers of cell death resistance and immune suppression, providing a potential therapeutic approach for large HCC, as summarized in Scheme 1.

## 2 Results and Discussion

### 2.1 Design and Physicochemical Characterization of Injectable TCPD Nanodepot

**FIGURE 1:**
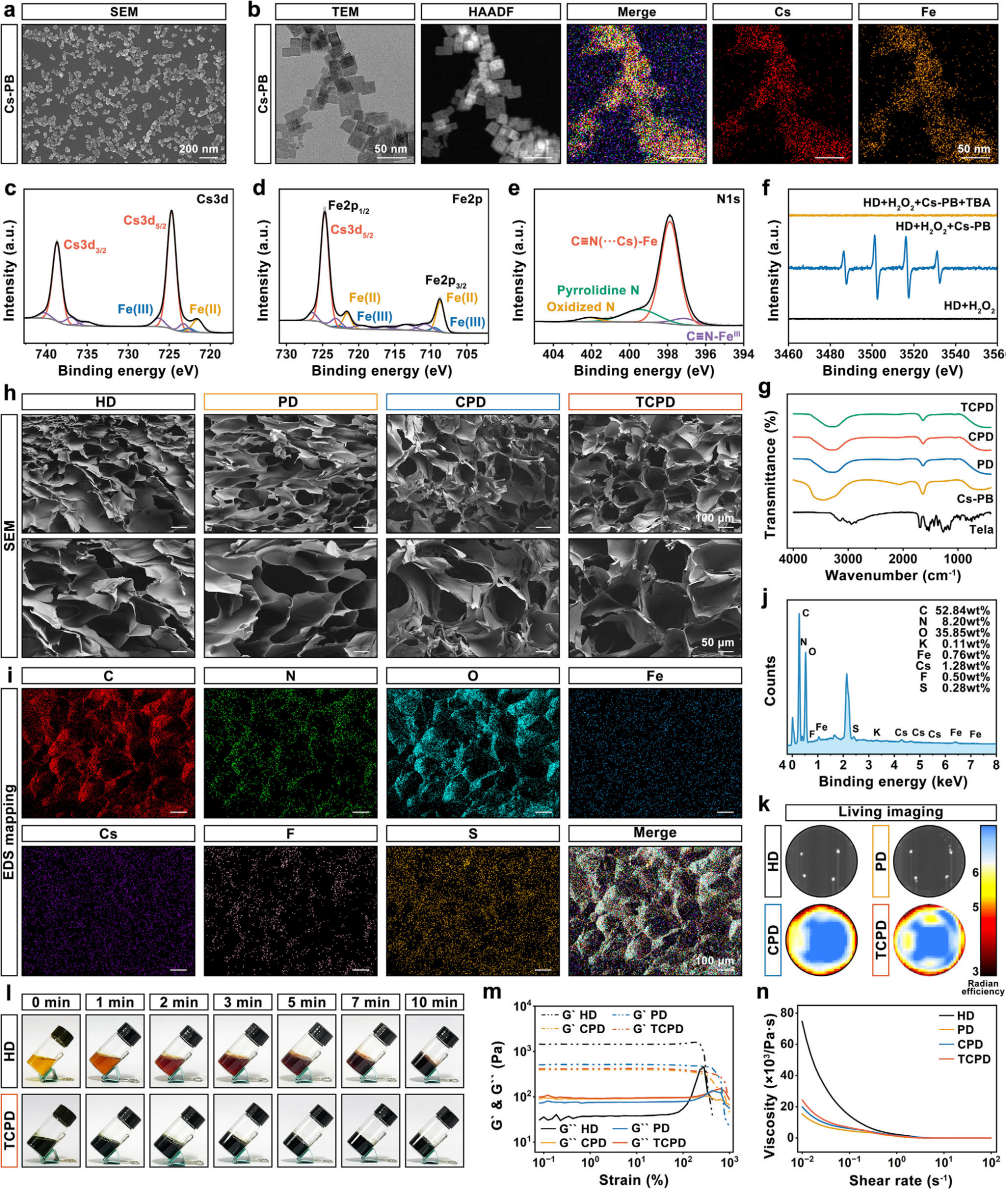
Physicochemical characterization of injectable dual‑crosslinked nanodepot. (a) Scanning electron microscope (SEM) images of Cs‐PB. (b) Transmission electron microscope (TEM), high‐angle annular dark‐field (HAADF‐STEM), and energy dispersive spectroscopy (EDS) elemental mapping of Cs‐PB. (c‐e) Cs3d, Fe2p, and N1s curve‐fitting analyses based on X‐ray photoelectron spectroscopy (XPS) of Cs‐PB. (f) Electron spin resonance (ESR) spectroscopy of hydroxyl radical during gelation. (g) Fourier transform infrared (FTIR) spectroscopy of Tela, Cs‐PB, HD, PD, CPD, and TCPD. (h) SEM images of HRP‐catalyzed HD hydrogel, PD, CPD, and TCPD nanodepots. (i‐j) EDS mapping images and analysis of TCPD nanodepot. (k) Ce6 fluorescence images of HRP‐catalyzed HD hydrogel, PD, CPD, and TCPD nanodepots. (l) Representative visual documentation of the gelation kinetics for HRP‐catalyzed HD hydrogel and TCPD nanodepot. (m) Rheological property profile of HRP‐catalyzed HD hydrogel, PD, CPD, and TCPD nanodepots. (n) Viscosity‐shear rate profile of HRP‐catalyzed HD hydrogel, PD, CPD, and TCPD nanodepots. Scale bars: 50 and 200 nm, and 50 and 100 µm.

To achieve the intended catalytic design, Cs‐PB was synthesized via a citrate‐capped dual‐precursor co‐precipitation method [45]. Morphological and crystallographic evaluations using scanning electron microscopy (SEM) and high‐resolution transmission electron microscopy (HRTEM) revealed that the resulting Cs‐PB maintained a highly uniform cubic architecture with well‐defined lattice fringes (Figure 1a and Figure S1a). Dynamic light scattering (DLS) analysis showed a hydrodynamic size of 42.8 ± 2.35 nm and a zeta potential of ‐47.27 ± 1.95 mV (Figure S1b). Elemental quantification by inductively coupled plasma optical emission spectrometry (ICP‐OES) yielded a Cs to Fe molar ratio of 1.65 ± 0.02, while energy‐dispersive X‐ray spectroscopy (EDS) mapping confirmed homogeneous elemental distribution (Figure 1b and Figure S1c). Structural validation via full‐pattern Rietveld refinement of powder X‐ray diffraction (PXRD) established a face‐centered cubic framework with a lattice parameter of a = 10.3985 Å, high relative crystallinity, and a Scherrer crystallite size of 43.4 nm (Figure S1d,e). Raman spectroscopy elucidated coordination characteristics, where a shift in the Eg peak alongside an enhanced ν(Fe^II^–CN–Fe^II^) shoulder peak indicated an FeN_6_ coordination sphere and high lattice geometry intactness (Figure S1f,g). X‐ray photoelectron spectroscopy (XPS) survey spectra showed no detectable potassium signals, confirming the absence of precursor residue (Figure S1h). High‐resolution Cs 3d XPS analysis displayed characteristic doublet peaks at 738.7 and 724.7 eV, corresponding to Cs^+^ ions situated in interstitial cavities, which elevates the Fe coordination number to favor radical‐generating FeN_5_ and FeN_6_ configurations (Figure 1c). Deconvolution of Fe 2p XPS spectra demonstrated an increased Fe(II)/Fe(III) ratio to maintain electroneutrality (Figure 1d), consistent with N 1s XPS features identifying CN(···Cs)–Fe species at 397.87 eV (Figure 1e) [46, 47, 48]. Cumulative cesium release remained below 10% under physiological conditions (pH 7.4) and reached 12.52 ± 0.66% under mildly acidic conditions (pH 6.5) over one week, confirming stable framework integration rather than loose surface adsorption (Figure S1i). These trace concentrations of released cesium remained well below cytotoxicity thresholds, as verified by CCK‐8 assays in both H22 tumor cells and L929 fibroblasts treated with free CsCl, where cell viabilities remained above 94% at the maximum released concentration range (5‐10 µM), ensuring biocompatibility of Cs‐PB (Figure S1j). To directly establish a structure‐function relationship, side‐by‐side comparative evaluations were conducted between Cs‐PB and conventional Prussian blue nanoparticles at matched iron concentrations, showing that Cs‐PB exhibited enhanced peroxidase‐like activity and improved MW‐thermal conversion performance (Figure S2). Thus, incorporated Cs^+^ ions function exclusively as biologically inert structural mediators that optimize lattice crystallinity and enhance catalytic performance.

To utilize this catalytic capability for biological applications, an in situ crosslinking strategy was employed that relies on the intrinsic peroxidase‐like activity of Cs‐PB. Carboxylate groups along the hyaluronic acid‐dopamine (HD) polymer backbone provide a mildly acidic microenvironment that establishes a favorable local catalytic niche, accelerating Cs‐PB‐mediated decomposition of hydrogen peroxide (H_2_O_2_) into hydroxyl radicals (•OH). These generated radicals oxidize catechol moieties on polymer chains to initiate covalent crosslinking, while surface‐exposed Fe^3+^ ions on the nanozyme concurrently form reversible coordination bonds with adjacent catechol groups to reinforce the hydrogel network [49]. Unlike conventional approaches that require exogenous horseradish peroxidase (HRP), this nanozyme construct serves both as a catalytic crosslinking initiator and as an integrated functional component [50, 51, 52]. To verify the radical‐driven crosslinking mechanism, electron spin resonance (ESR) spectroscopy was performed using 5,5‐dimethyl‐1‐pyrroline N‐oxide (DMPO) as a spin‐trapping agent. Upon introducing H_2_O_2_ into the Cs‐PB‐containing precursor solution, a characteristic 1:2:2:1 quartet signal corresponding to the DMPO‐•OH adduct was detected, whereas this signal was absent in the nanozyme‐free control. Furthermore, inclusion of selective •OH scavenger *tert*‐butanol (TBA) eliminated both the radical signal and macroscopic hydrogel formation, confirming that •OH generation is essential for the crosslinking process (Figure 1f).

Systematic incorporation of this catalytic principle into a functional therapeutic platform was achieved through encapsulation of Ce6 and/or Tela into Cs‐PB‐catalyzed HD nanodepots, yielding formulations designated as Cs‐PB@HD (PD), Ce6/Cs‐PB@HD (CPD), and Tela/Ce6/Cs‐PB@HD (TCPD), respectively (Table S1). Cs‐PB exhibited peroxidase‐like activity of 583.69 ± 23.51 U/mg (pH 6.5, 25 °C), and HRP‐catalyzed HD hydrogels were prepared as controls at equivalent peroxidase activity for comparative analysis. Gelation of HD hydrogels and TCPD nanodepots was observed in Figure S3. Quantitative analysis across three independent batches demonstrated encapsulation efficiencies of 98.73 ± 0.48% for Cs‐PB, 96.94 ± 1.59% for Ce6, and 95.47 ± 0.93% for Tela in TCPD nanodepots, with corresponding wet‐weight loading contents detailed in Figure S4. Fourier transform infrared (FTIR) spectroscopy characterized the chemical composition of Tela, Cs‐PB, and PD, CPD, and TCPD nanodepots. As shown in Figure 1g, characteristic FTIR peaks of Tela were identified at 696 cm^−1^ for C–S–C stretching vibration of thiadiazole ring, 1267 cm^−1^ and 1156 cm^−1^ for C–F stretching and C–O–C stretching vibrations of –OCF_3_ group, 1697 cm^−1^ for C═O stretching of amide I band, 1529 cm^−1^ for N–H in‐plane bending vibration of amide II band, and 2859 cm^−1^ and 2947 cm^−1^ for asymmetric and symmetric C–H stretching vibrations of methylene groups within butyl linker chain connecting aromatic rings [53, 54]. While strong baseline absorption from the biopolymeric HD backbone partially obscured these functional signatures in FTIR and Raman spectra (Figure S5), spatial compositional mapping confirmed homogeneous integration. Specifically, SEM coupled with EDS mapping illuminated that PD, CPD, and TCPD nanodepots maintained an interconnected mesh‐like microporous structure with smoothly contoured pore walls comparable to HD hydrogels, while displaying uniform spatial distribution of carbon, nitrogen, and oxygen throughout the matrix, along with iron and cesium as elemental signatures of Cs‐PB, and fluorine and sulfur as markers of Tela, thereby confirming successful integration of Cs‐PB and Tela into TCPD nanodepots (Figure 1h–j). Successful Ce6 incorporation was validated using an IVIS Spectrum in vivo imaging system, which detected characteristic fluorescence signals in both CPD and TCPD nanodepots, consistent with the intrinsic optical profile of Ce6 (Figure 1k).

Dynamic gelation kinetic profiles demonstrated an accelerated gelation process for Cs‐PB‐catalyzed nanodepots (PD, CPD, and TCPD), which achieved complete phase transition within 5 min compared to 10 min required for HRP‐catalyzed HD hydrogels (Figure 1l and Figure S6). This acceleration may be attributed to Cs‐PB‐catalyzed Fenton‐like generation of •OH that exhibits faster reaction kinetics than HRP‐mediated ferryl species, while concurrent Fe‐catechol coordination spatially constrains polymer chains to accelerate radical‐mediated covalent crosslinking [45, 55]. Subsequently, push injection experiments using syringes with 23G and 26G needles confirmed that TCPD nanodepots exhibit smooth injectability, facilitating percutaneous intratumoral injection (Figure S7). Stability and injectability of nanodepots were further evaluated by rheological testing. Dynamic strain amplitude sweeps revealed that all formulations maintained a predominantly elastic solid‐like state (G′ > G″) across the 1%–100% low‐strain range, indicating an intact three‐dimensional (3D) polymeric network. Under increasing mechanical strain, standard HD hydrogel underwent a gel‐to‐sol phase transition (G′ < G″) at approximately 300% strain, whereas the Cs‐PB‐catalyzed nanodepots extended the linear viscoelastic crossover threshold to 700–800% strain, reaching a liquid‐like sol state at 1000% strain (Figure 1m). In parallel, all formulations displayed a decrease in viscosity with increasing shear rate, exhibiting characteristic shear‐thinning behavior, with Cs‐PB‐catalyzed nanodepots displaying lower viscosities than HD hydrogel across the tested shear rate range (Figure 1n). These rheological features stem from reversible Fe^3+^‐catechol coordination bonds, which function as sacrificial crosslinks to dissipate mechanical energy while reducing polymer chain entanglement and facilitating chain alignment under shear. Collectively, increased critical strain supports structural integrity under mechanical deformation in tumor tissues, while shear‐thinning behavior enables low injection resistance through fine needles [56, 57], providing adequate depot retention and stability for subsequent spatially complementary sono‐thermal ablation.

Thermogravimetric analysis revealed two‐stage thermal degradation profiles for all formulations. Initial water loss (8%–10%) occurred between 30–100°C, followed by polymer matrix decomposition above 200°C. Incorporation of Cs‐PB, Ce6, and Tela did not alter thermal stability compared to the HD control, with all groups maintaining >85% residual mass at 200°C (Figure S8). In parallel, swelling behavior analysis demonstrated that dual‐crosslinked nanodepots (PD, CPD, and TCPD) maintained lower equilibrium swelling ratios relative to the HD hydrogel (Figure S9a). The reduced swelling ratio of Cs‐PB‐catalyzed nanodepots is attributed to their dual covalent‐coordination network architecture, which increases effective crosslinking density and results in a more compact network structure. In contrast, HRP‐catalyzed hydrogels rely on a single covalent crosslinking network, where polymer chain segments retain higher mobility and water molecules more readily penetrate the network. Hemolysis experiments confirmed that injectable nanodepots exhibited minimal hemolytic activity (all hemolysis rates below 5%), demonstrating biocompatibility for in vivo applications (Figure S9b).

### 2.2 Integrated Catalytic Activity, MW Thermal Conversion, and Stimuli‐Responsive Release Profiles

**FIGURE 2:**
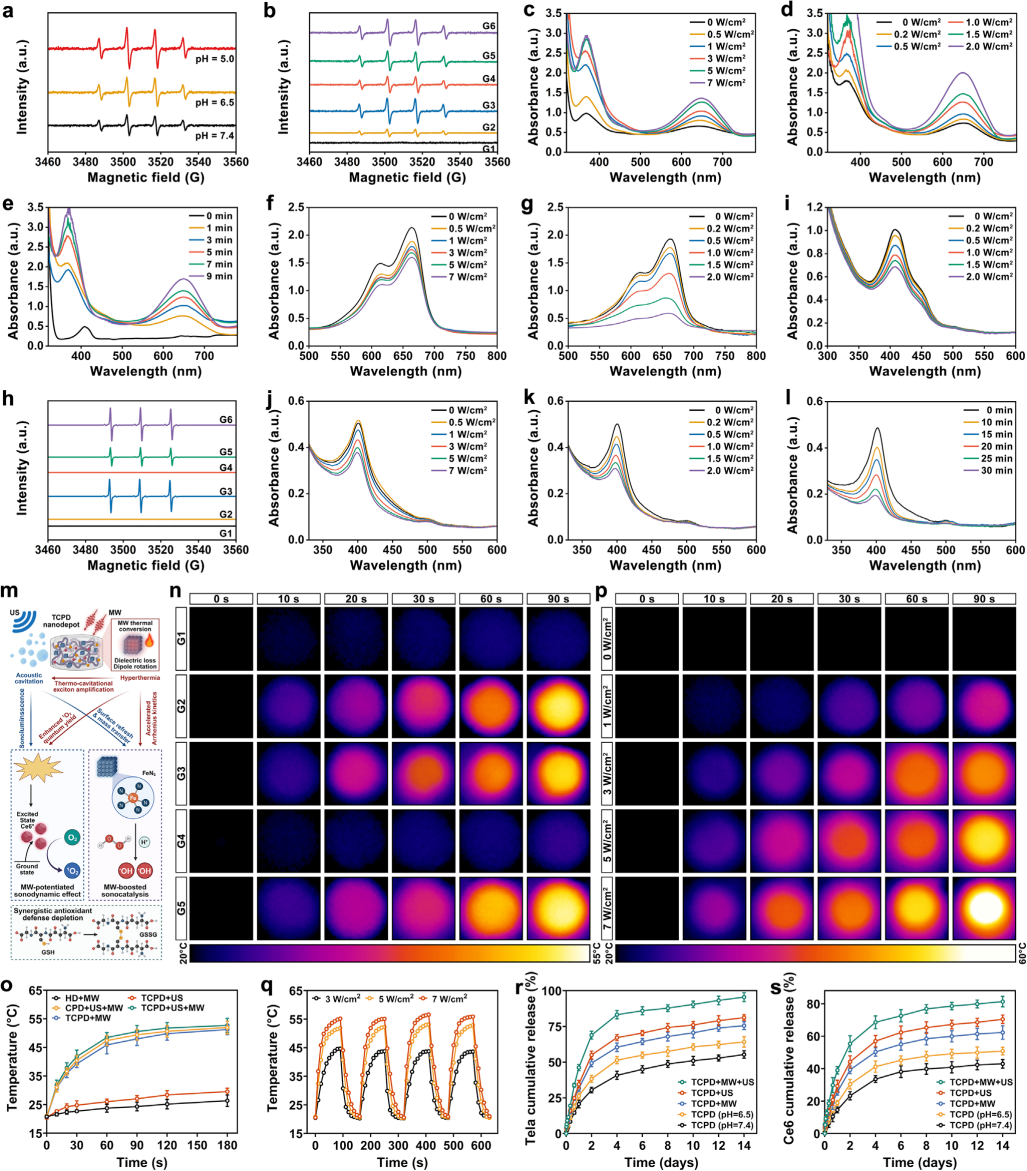
Integrated catalytic activity, MW thermal conversion, and stimuli‐responsive release profiles. (a‐b) ESR spectroscopy for hydroxyl radical detection at varying pH and under various interventions. (c) Absorbance changes of the TMB probe under different experimental conditions. (d‐e) Dependency of TMB absorbance on MW and US power with TCPD nanodepots (20 mg/mL). (f‐g) Dependency of MB absorbance on MW and US power with the TCPD nanodepots (20 mg/mL). (h) ESR spectroscopy for singlet oxygen detection under various interventions. (i) US power‐dependent absorbance changes of DPBF probe treated with TCPD nanodepots (20 mg/mL). (j) Absorbance changes of the DTNB probe under various interventions. (k‐l) MW and US power‐dependent absorbance changes of the DTNB probe with the TCPD nanodepots (20 mg/mL). (m) Schematic representation of the catalytic reactions of TCPD triggered by MW and US irradiation. (n‐o) Infrared thermographic images and corresponding temperature‐time profiles under various interventions. (p) Infrared thermography of the TCPD nanodepot under varying MW power. (q) Temperature cycles of the TCPD nanodepot under graded MW power (3, 5, and 7 W/cm^2^). (r‐s) Cumulative in vitro release profiles of Tela and Ce6. Data represent n = 3 biologically independent experiments and are presented as mean ± standard deviation.

To investigate catalytic cascades of TCPD nanodepots, ESR spectroscopy was conducted to monitor •OH generation. In the engineered Cs‐PB framework, high‐coordinated FeN_5_ sites require an acidic environment to facilitate the H^+^‐assisted homolytic cleavage of endogenous H_2_O_2_ [45]. Consistent with this mechanism, TCPD nanodepots generated strong DMPO‐•OH spin‐adduct signals under acidic conditions (pH 5.0), whereas negligible signals were observed under physiological conditions (Figure 2a). To evaluate the multimodal catalytic performance, ESR spectra were compared across six groups: PBS control (G1), TCPD alone (G2), CPD with US and MW irradiation (G3), TCPD with MW (G4), TCPD with US (G5), and TCPD with dual US and MW irradiation (G6). Compared with single‐stimulus or carrier controls, concurrent dual‐energy irradiation in G3 and G6 markedly increased the DMPO‐•OH signal intensities (Figure 2b). Moreover, TMB was utilized as a chromogenic substrate to capture oxidants (λ = 652 nm). To verify potential optical interference from Cs‐PB pigmentation and nanomaterial scattering, UV–vis absorption spectra of individual components, including probes alone, H_2_O_2_ alone, TCPD extract alone, and probes incubated with TCPD extract, were measured under identical conditions (Figure S10a). While single‐modality stimulation in G4 and G5 produced moderate catalytic activity, dual stimulation in G3 and G6 resulted in substantially higher absorbance, demonstrating enhanced peroxidase‐like activity (Figure S10b). Systematic optimization of reaction parameters for G6 confirmed that TMB oxidation increased in a concentration‐dependent (0, 3, 5, 10, 20, and 30 mg/mL), power density‐dependent (MW: 0, 0.5, 1, 3, 5, and 7 W/cm^2^, 2.45 GHz; US: 0, 0.2, 0.5, 1, 1.5, and 2 W/cm^2^, 1 MHz), and time‐dependent (0, 1, 3, 5, 7, and 9 min) manner (Figure 2c–e and Figure S10c). Consistently, •OH‐mediated oxidative degradation of methylene blue (MB) at 664 nm showed a similar trend, with G6 demonstrating the most extensive MB degradation in a power density‐dependent manner (Figure 2f,g and Figure S11). Mechanistically, US irradiation induces microbubble formation and collapse, generating high‐velocity microjets that disrupt the diffusion boundary layer around the Cs‐PB surface and promote substrate mass transfer to accelerate the sonocatalytic Fenton‐like reaction [58]. Meanwhile, MW exposure utilizes dielectric loss of structural nodes to induce localized hyperthermia, elevating kinetic energy according to Arrhenius principles and accelerating reactant molecular collisions [59]. Integrating these modalities produces a dielectro‐acoustic synergy where MWA reduces the threshold for acoustic cavitation [36].

Distinct from sonocatalytic Fenton‐like reaction, applied US concurrently initiates SDT mediated by organic sensitizer Ce6 loaded in TCPD nanodepots. During SDT, US‐induced acoustic cavitation generates sonoluminescence, functioning as an internal light source to excite ground‐state Ce6, which subsequently transfers energy to ambient tissue oxygen to generate cytotoxic singlet oxygen (^1^O_2_) [60, 61, 62]. This independent SDT mechanism was confirmed via ^1^O_2_‐specific ESR analysis utilizing the 2,2,6,6‐tetramethylpiperidine (TEMP) spin trap (Figure 2h). Acoustic excitation initiated mechanical cavitation to produce clear TEMP‐^1^O_2_ adduct signals in G5. Integration of simultaneous MW further increased adduct intensity to its maximum in G6. This synergy likely arises from multiple thermally facilitated processes whereby Cs‐PB‐mediated MW hyperthermia may enhance oxygen diffusion kinetics, accelerate Ce6 release, and increase the temperature‐dependent bimolecular reaction rates between Ce6 triplet states and molecular oxygen, thereby amplifying overall sonodynamic ^1^O_2_ generation efficiency [63, 64]. Macroscopic validation using ^1^O_2_‐specific DPBF probe supported this mechanistic synergy. To exclude potential optical interference from Ce6 absorption and nanomaterial scattering, UV–vis absorption spectra of individual components were measured under identical conditions (Figure S12a). G6 produced maximal probe decay at 410 nm, demonstrating enhanced activity compared to other groups (Figure S12b). Multidimensional parameter optimization for G6 demonstrated that DPBF depletion was dependent on both TCPD nanodepot concentration (0, 3, 5, 10, 20, and 30 mg/mL) and US power density (up to 2.0 W/cm^2^) over a 9‐min irradiation window (Figure 2i and Figure S12c,d). These results demonstrate that TCPD nanodepots utilize combined US and MW fields to execute a dual‐track process that couples sonocatalysis for •OH generation with sonodynamic action for ^1^O_2_ production.

To assess the consumption of cellular antioxidant defenses, GSH depletion was systematically examined using Ellman's reagent (DTNB), where reduction of the TNB^2−^ absorbance peak at 412 nm served as a measure of thiol consumption. Fe^3+^ nodes within Cs‐PB directly abstract electrons from intracellular GSH. Under external stimulation, MW hyperthermia accelerates this interfacial electron transfer [65]. Concurrently, US‐induced acoustic microstreaming reduces local diffusion barriers and refreshes exposed catalytic sites [66]. Additionally, generated •OH and ^1^O_2_ drive direct chemical oxidation of residual thiols [67, 68, 69]. To exclude potential optical interference, UV–vis absorption spectra of individual components, including DTNB reagent alone, DTNB reagent incubated with GSH, TCPD nanodepots extract alone, and DTNB reagent incubated with GSH and TCPD nanodepots extract, were measured under identical conditions (Figure S13a). Consequently, G6 exhibited the greatest decrease in DTNB absorbance compared to other groups (Figure S13b). Over a 30 min incubation period, GSH depletion showed clear dependence on material concentration, MW/US power density, and irradiation duration (Figure 2j–l and Figure S13c). Through integration of MW/US‐accelerated GSH depletion and dual‐track ROS generation, TCPD nanodepots have potential to induce substantial oxidative stress in target cells (Figure 2m).

Intrinsic MW thermal conversion performance of TCPD nanodepots was evaluated by real‐time infrared thermographic mapping across different configurations. Groups lacking either Cs‐PB or MW exhibited negligible temperature elevation, confirming that US irradiation alone contributes minimally to macroscopic heating. In contrast, all Cs‐PB‐containing groups subject to MW exhibited rapid thermal responses, reaching temperatures of 49.4 ± 1.8 °C, 48.0 ± 2.3 °C, and 50.6 ± 2.1 °C at 90 s, respectively (Figure 2n,o). This thermal effect is attributed to the dielectric loss of confined Cs^+^ ions and structural water molecules within the Cs‐PB lattice, which undergo dipole rotation and orientational polarization under the MW field [70]. Parametric analysis further demonstrated that MW‐thermal conversion output correlated directly with both TCPD concentration and MW power density (Figure 2p and Figure S14). Furthermore, TCPD nanodepots maintained consistent peak temperatures across four consecutive heating and cooling cycles under graded MW power densities of 3, 5, and 7 W/cm^2^, displaying minimal thermal attenuation (Figure 2q). This reproducible and tunable MW‐thermal conversion performance confirms that TCPD nanodepots serve as effective converters for sensitizing MWA.

Subsequently, in vitro release profiles of Tela and Ce6 from TCPD nanodepots were evaluated at different pH values (pH 7.4 and 6.5, 37°C) with or without external physical stimuli. Release of both Tela and Ce6 from stimuli‐responsive nanodepots can be divided into an initial burst release phase (0–6 days) and a subsequent constant steady release phase (7–14 days). The first burst release can rapidly exert drug efficacy to achieve synergistic anti‐tumor effects, while later sustained release can maintain effective drug concentration locally to continuously exert therapeutic efficacy. Without external stimulation, incubation in a mildly acidic medium (pH 6.5) accelerated Tela release during the first 6 days compared with physiological conditions (pH 7.4). Application of combined US and MW irradiation further promoted drug release, with cumulative Tela release reaching 86.01 ± 2.84% on day 6 and 95.56 ± 3.16% on day 14 (Figure 2r). Furthermore, Ce6 displayed consistent stimuli‐responsive release patterns from nanodepots. Under non‐stimulated conditions at pH 6.5, cumulative Ce6 release reached 50.84 ± 2.39%, whereas dual US and MW irradiation increased cumulative release to 81.33 ± 3.25% on day 14 (Figure 2s). Collectively, such on‐demand release kinetics reflect a diffusion‐mediated transport mechanism enhanced by acid‐promoted matrix relaxation and external bimodal stimulation‐induced structural disruption.

### 2.3 Disrupting the Glutaminolysis‐TCA Cycle Axis to Potentiate Synergistic Oxidative Stress

Prior to detailed mechanistic investigations, comprehensive cytotoxicity screening via Cell Counting Kit‐8 (CCK‐8) assays was performed across an expanded spectrum of formulation controls and physical stimulation to systematically deconvolute the therapeutic contribution of each component. As shown in Figure S15, the HD, PD, and CPD nanodepots alone, as well as the MW and US irradiation alone, exhibited negligible intrinsic cytotoxicity. Moreover, free Ce6 combined with US, free Cs‐PB or Tela combined with MW and US irradiation, as well as PD nanodepots combined with MW and US irradiation, demonstrated significantly lower efficacy compared to the integrated TCPD nanodepots. Based on these preliminary findings, six experimental groups were established to evaluate the therapeutic mechanisms as follows: (G1) Control, (G2) TCPD, (G3) CPD+US+MW, (G4) TCPD+MW, (G5) TCPD+US, and (G6) TCPD+US+MW.

**FIGURE 3:**
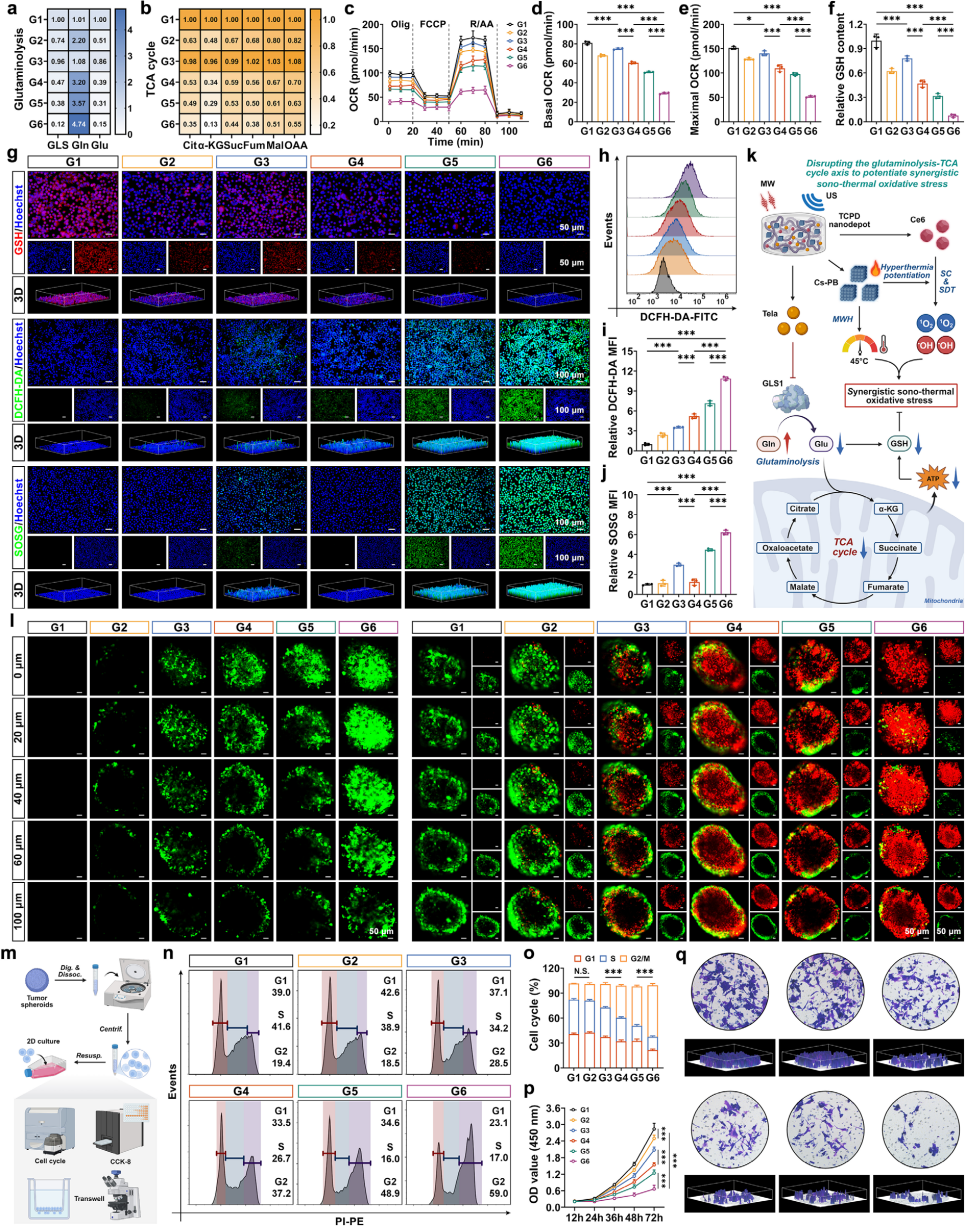
Disrupting the glutaminolysis‐TCA axis sensitizes tumors to spatially complementary sono‐thermal ablation. (a) Glutaminolysis‐related components assessment. (b) TCA cycle intermediates assessment. (c‐e) Oxygen consumption rate (OCR) kinetic profiles and corresponding basal and maximal OCR evaluation. (f) Intracellular GSH content determination. (g) Representative GSH, and DCFH‐DA and SOSG fluorescence staining images. (h‐i) Flow cytometry image and statistical analysis of DCFH‐DA relative mean fluorescence intensity (MFI). (j) Statistical analysis of SOSG relative MFI. (k) Schematic diagram of bimodal‐responsive TCPD nanodepot potentiating synergistic oxidative stress via dismantling the glutaminolysis‐TCA cycle axis. (l) DCFH‐DA and Live/Dead staining images for three dimensions tumor spheroids. (m) Schematic diagram of spheroid dissociation for single‐cell functional assays. (n‐o) Flow cytometry images and statistical analysis of the cell cycle. (p) Cell proliferation profiles measured by the CCK‐8 assay. (q) Representative images of the Transwell migration assay. Data represent n = 3 biologically independent experiments and are presented as mean ± standard deviation. Statistical comparisons were performed using one‐way ANOVA with Tukey's multiple comparisons test. Scale bars: 50 and 100 µm.

Encouraged by the favorable stimulus‐responsive release profiles, the metabolic impact of TCPD nanodepots on glutaminolysis was systematically investigated at the cellular level. Relative GLS1 activity was first quantified across all treatment groups. Compared to G1, G4 and G5 displayed intermediate suppression, and G6 resulted in marked GLS1 activity downregulation, with relative activity decreasing to 0.12 ± 0.04 (Figure 3a). This progressive reduction pattern corresponded with differential drug release kinetics governed by physical stimulation. Furthermore, G3 maintained GLS1 activity levels comparable to G1, confirming that non‐Tela components did not affect GLS1 activity. To determine downstream metabolic consequences of GLS1 inhibition, intracellular Gln and Glu levels were quantified using commercial assay kits (Table S2). G2 showed minimal metabolic variation, G4 and G5 induced progressive inhibition of metabolic flux, and G6 induced a 4.7 fold accumulation of intracellular Gln alongside a concomitant reduction in Glu to 0.15 ± 0.03 relative to G1, culminating in a 31.6 fold increase in the intracellular Gln/Glu ratio (Figure 3a). Meanwhile, metabolite levels in G3 remained indistinguishable from G1, indicating that non‐Tela components did not affect glutaminolysis.

Given that Glu‐derived α‐ketoglutarate serves as a primary anaplerotic substrate for the TCA cycle, intracellular Glu depletion was anticipated to impair downstream metabolic flux [71, 72, 73]. To test this hypothesis, key TCA cycle intermediates including α‐ketoglutarate, citrate, succinate, fumarate, malate, and oxaloacetate were quantitatively profiled (Figure 3b and Table S2). In accordance with upstream glutaminase blockade, Tela‐containing groups exhibited significant depletion of these metabolites, with the most pronounced reduction occurring in G6 owing to restricted anaplerotic carbon entry. This metabolic disruption directly translated into bioenergetic impairment, as evidenced by progressive decline in mitochondrial respiration capacity across G4 to G6 assessed by Seahorse XF analysis. Consistent with the metabolite depletion profiles, G2 induced a modest reduction in oxygen consumption rate (OCR), whereas the single‐stimulus G4 and G5 displayed intermediate respiratory inhibition. G6 exhibited substantial mitochondrial dysfunction, characterized by marked decreases in both basal and maximal OCR (Figure 3c–e). Parallel to the OCR profiles, G6 displayed pronounced depletion of intracellular adenosine triphosphate (ATP), confirming substantial loss of cellular energy homeostasis. In contrast, preserved respiratory parameters and stable ATP pools observed in G3 confirmed that non‐Tela components did not disrupt cellular bioenergetics (Figure S16a). Collectively, these integrated metabolic profiles revealed distinct hierarchical bioenergetic vulnerability, wherein the bimodal‐responsive release of Tela in G6 effectively disrupts the glutaminolysis‐TCA cycle axis.

Beyond sustaining mitochondrial bioenergetics, glutaminolysis is coupled to the cellular antioxidant defense network through the production of Glu, which serves as the essential precursor for GSH biosynthesis [73]. As shown in Figure 3f, G6 induced the most significant GSH depletion, with relative levels decreasing to 7.33 ± 2.08% of G1, thereby substantially compromising metabolic resistance to oxidative stress. Fluorescence staining with GSH‐Tracker NIR confirmed a similar trend (Figure 3g and Figure S16b). Conversely, intracellular GSH pools in G3 remained substantially preserved. Total intracellular ROS levels were evaluated using the oxidation‐sensitive fluorescent probe DCFH‐DA, while ^1^O_2_ was specifically detected with the Singlet Oxygen Sensor Green (SOSG) probe. Fluorescence microscopy revealed a graded increase in DCFH‐DA signals, with G2 and G3 displaying only minimal green fluorescence, whereas G4 and G5 exhibited moderate signals (Figure 3g). G6 displayed the most intense intracellular luminescence, and flow cytometric quantification confirmed a substantial ROS accumulation, as the relative mean fluorescence intensity (MFI) in the G6 group reached 11.48 ± 0.60 (Figure 3h,i). Concurrently, SOSG staining revealed the strongest ^1^O_2_‐specific signal in G6, markedly exceeding that in G5 (Figure 3j). Collectively, these findings demonstrate that the bimodal‐responsive TCPD nanodepots achieve a self‐reinforced oxidative stress cascade, wherein Tela‐mediated glutaminolysis blockade induces concurrent bioenergetic depletion and antioxidant vulnerability, effectively sensitizing tumor cells to the ROS burst generated by combined MWA and SDT (Figure 3k).

### 2.4 Bimodal‐Responsive TCPD Nanodepot Empowers Spatially Complementary Sono‐Thermal Ablation

The spatially complementary therapeutic effect of the bimodal‐responsive TCPD nanodepots was investigated in 3D tumor spheroids using the DCFH‐DA probe. Confocal laser scanning microscopy (CLSM) was performed to acquire sequential optical sections along the Z‐axis at depths of 0, 20, 40, 60, and 100 µm (Figure 3l). Tumor spheroids in G1 exhibited negligible green fluorescence across all scanning planes, confirming baseline redox homeostasis in the untreated model. Similarly, G2 showed minimal fluorescence throughout the spheroid, indicating that nanodepot incubation without physical actuation did not induce appreciable oxidative stress. G3 generated moderate and relatively uniform fluorescence signals from the periphery to the internal core, reflecting simultaneous activation of MWA and SDT effects. Upon incorporating metabolic inhibition, distinct spatial ROS distribution patterns were observed. G4 displayed a core‐preferential profile, in which superficial DCFH‐DA signals intensified progressively toward the deeper layers. This pattern is consistent with the pronounced tissue‐penetration capability of MWA, allowing thermal stimulation to effectively elevate ROS levels within the core. Conversely, G5 exhibited intense fluorescence predominantly restricted to the peripheral zone, confirming that US cavitation and localized ROS generation are spatially constrained within the superficial layers. G6 demonstrated the highest and most homogeneous fluorescence signals throughout the entire spheroid, confirming that Tela‐mediated metabolic inhibition effectively cooperates with spatial complementarity of core‐penetrating MWA and surface‐active SDT to establish pan‐spheroidal oxidative stress.

To evaluate the final therapeutic efficacy, Live/Dead fluorescence staining was performed on tumor spheroids, with spatial viability profiles visualized through 3D Z‐stack confocal reconstructions (Figure 3l). G1 displayed predominantly viable cells across all imaging planes, whereas G2 exhibited only minimal and diffuse dead‐cell fluorescence. G3 showed a moderate increase in dead cells throughout the volume, though the cytotoxic effect remained incomplete. Spatial cell death patterns in G4 and G5 directly corresponded to the localized energy deposition profiles of their respective physical modalities. Specifically, G4 displayed a core‐preferential cell death pattern while retaining a viable superficial layer, whereas G5 exhibited an outer cell death pattern predominantly confined to the peripheral zone. The integrated G6 demonstrated extensive and homogeneous dead‐cell signals throughout the entire spheroid, characterized by uniform red fluorescence extending from the exterior margin to the internal core. Collectively, these results provide direct visual evidence that TCPD nanodepot‐empowered spatially complementary sono‐thermal ablation achieved cytotoxic coverage across 3D tumor spheroids. This therapeutic synergy is driven by the integration of metabolic sensitization and dual‐modality physical actuation, wherein superficial sonodynamic oxidative damage eliminates viable peripheral tumor margin to effectively complement the core‐focused MWA.

To assess the proliferative capacity and migratory potential of tumor cells surviving treatments, tumor spheroids from different groups were dissociated into single‐cell suspensions for subsequent functional assays (Figure 3m). Cell cycle distribution was quantified using propidium iodide (PI) staining and flow cytometry (Figure 3n,o). G1 displayed typical cell cycle dynamics, with approximately 40% of the population distributed in G0/G1 phase and a comparable fraction in S phase. Conversely, groups subjected to Tela intervention and/or physical stimulation demonstrated G2/M phase arrest at varying degrees. G6 displayed the most pronounced cell cycle perturbation, with S‐phase fraction reduced to 16.00 ± 0.92% and G2/M phase population increased to 61.70 ± 2.34%. This cell cycle arrest was further examined through longitudinal CCK‐8 proliferation assays (Figure 3p). Specifically, G6 exhibited sustained growth suppression across 72 h, with optical density values remaining near the baseline levels. To assess migratory propensity, surviving populations' motility was examined using a Transwell assay (Figure 3q and Figure S16c). Although G4 and G5 exhibited significantly impaired migration, G6 showed more complete inhibition of cell migration. Taken together, these results indicate that beyond inducing tumor cell death, the TCPD nanodepot‐empowered spatially complementary sono‐thermal ablation effectively suppresses both proliferative and migratory capacities of residual cells, thereby attenuating phenotypes associated with recurrence and metastasis.

### 2.5 Transcriptomic Profiling and Molecular Mechanisms of PANoptosis Execution

**FIGURE 4:**
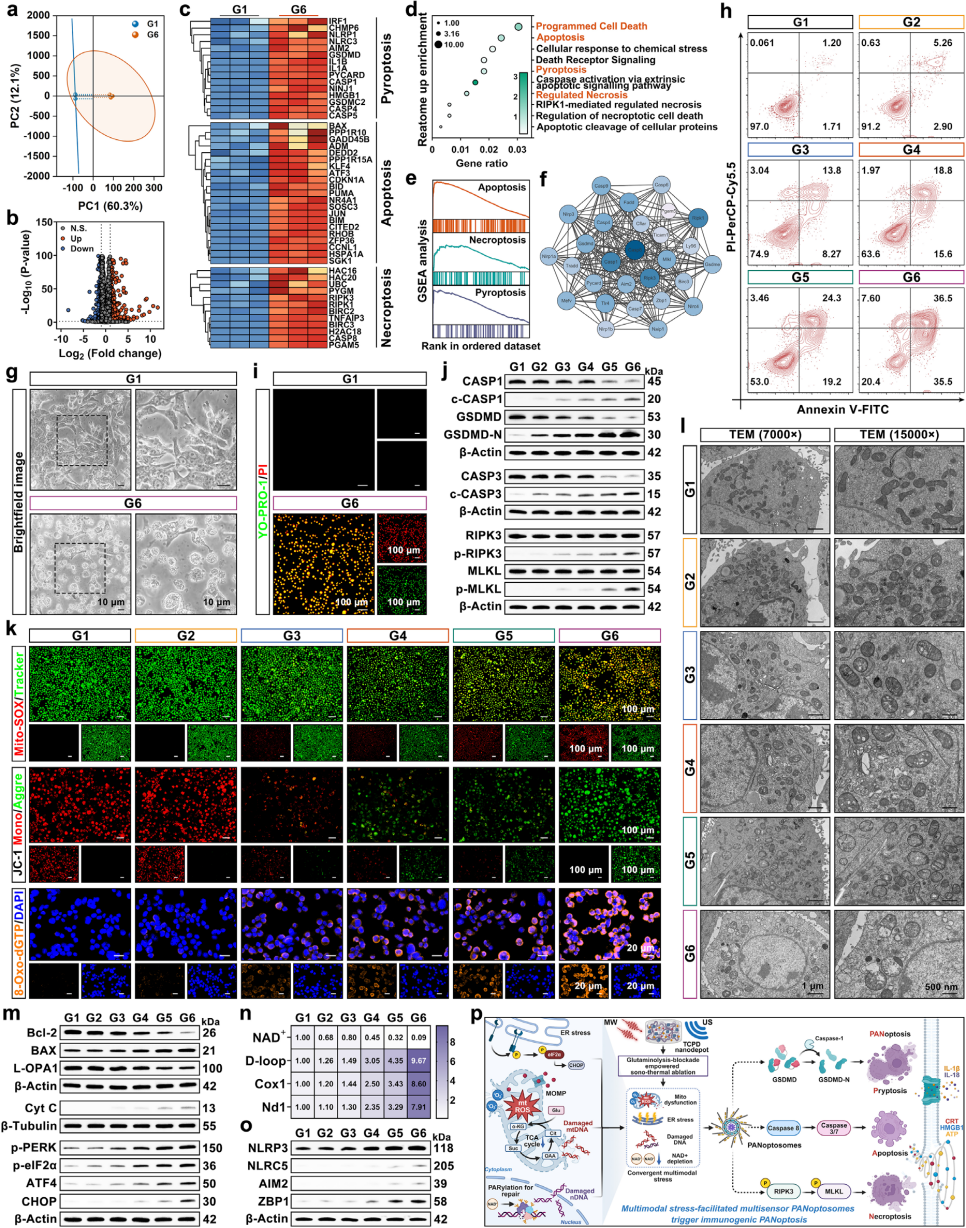
Convergent multimodal stress facilitates the assembly of multisensor PANoptosomes to drive PANoptosis. (a) Principal component analysis (PCA) from bulk RNA sequencing (RNA‐seq). (b) Volcano plot. (c‐f) Heatmap, Reactome enrichment analysis, Gene Set Enrichment Analysis (GSEA), and protein‐protein interaction (PPI) network of death‐related differentially expressed genes (DEGs). (g) Representative brightfield images in G1 and G6. (h) Flow cytometry detection of cell apoptosis. (i) Representative images of YO‐PRO‐1/PI staining. (j) Western blotting for PANoptosis executioners. (k) Representative fluorescence staining images of mito‐SOX, JC‐1 and 8‐oxo‐dGTP. (l) Representative TEM images showing ultrastructural morphological changes. (m) Western blot analysis of proteins expression related to mitochondrial outer membrane permeabilization and endoplasmic reticulum stress. (n) Quantitative analysis of intracellular NAD^+^ levels and cytosolic mtDNA content. (o) Western blot detection of PANoptosome assembly components. (p) Schematic diagram of multimodal stress‐facilitated multisensor PANoptosomes triggering immunogenic PANoptosis. Statistical analysis data represent n = 3 biologically independent experiments and are presented as mean ± standard deviation. Statistical comparisons were performed using one‐way ANOVA with Tukey's multiple comparisons test. Scale bars: 500 nm, and 1, 10, 20, and 100 µm.

To characterize transcriptomic alterations induced by TCPD nanodepot‐empowered spatially complementary sono‐thermal ablation, high‐throughput RNA sequencing was performed on tumor cells from G1 and G6. Principal component analysis (PCA) revealed distinct transcriptional profiles between the two groups (Figure 4a). The first principal component, accounting for 60.3% of total variance, effectively separated G6 from G1, indicating a shift in the gene expression landscape. Concordance between biological replicates within each group along the second principal component (12.1% variance) demonstrated reproducible transcriptional response. As shown in Figure 4b, G6 resulted in 866 upregulated and 411 downregulated differentially expressed genes (DEGs) compared to G1 (fold change ≥ 1, p < 0.05). Gene Ontology (GO) Biological Process (BP) enrichment analysis revealed that downregulated DEGs were associated with regulation of cell growth, response to oxidative stress, and cell cycle processes (Figure S17a). Similarly, Kyoto Encyclopedia of Genes and Genomes (KEGG) enrichment analysis showed that downregulated DEGs were enriched in the PI3K‐Akt signaling pathway, glutathione metabolism, and the JAK‐STAT signaling pathway (Figure S17b). These findings were consistent with functional experimental data. In‐depth examination of gene expression profiles associated with cell death revealed increased DEGs related to pyroptosis, apoptosis, and necroptosis following treatment (Figure 4c). Reactome enrichment analysis and gene set enrichment analysis (GSEA) indicated significant upregulation of pathways associated with programmed cell death, apoptosis, pyroptosis, and necroptosis (Figure 4d,e and Figure S17c–f). This coordinated activation of multiple cell death modalities is consistent with PANoptosis, an inflammatory programmed cell death [74, 75]. Key components of the PANoptosome, a molecular complex that mediates PANoptosis, including NLRP3, ZBP1, receptor‐interacting protein kinase 3 (RIPK3), and apoptosis‐associated speck‐like protein containing a CARD (ASC), were upregulated, with STRING analysis revealing extensive protein‐protein interactions among these molecules (Figure 4f). In summary, the transcriptomic landscape indicated coordinated activation of multiple regulated cell death pathways during treatment.

To determine whether TCPD nanodepot‐empowered spatially complementary sono‐thermal ablation triggered PANoptosis, key death pathways were analyzed (antibody details in Tables S3 and S4). Bright‐field microscopy showed that G6 exhibited distinct morphological hallmarks of pyroptosis, including cellular swelling and cytoplasmic bubbles (Figure 4g). Western blot analysis showed proteolytic conversion of caspase‐1 into its cleaved subunit and processing of GSDMD into its active N‐terminal fragment in G6 (Figure 4j and Figure S18a–d). This GSDMD activation was accompanied by extracellular release of lactate dehydrogenase (LDH) and proinflammatory cytokines IL‐1β and IL‐18 in G6 (Figure S19). Simultaneously, the apoptotic component was evaluated via Annexin V/PI flow cytometry, and G6 exhibited early and late apoptotic fractions of 37.25 ± 1.89% and 40.42 ± 3.40%, respectively (Figure 4h and Figure S20). This apoptotic response was accompanied by depletion of caspase‐3 and accumulation of cleaved caspase‐3 (c‐caspase‐3) in G6 (Figure 4j and Figure S18e, f). To track plasma membrane permeability during treatment‐induced cell death, YO‐PRO‐1/PI dual staining was performed. YO‐PRO‐1 single‐positive cells (green fluorescence) reflect early membrane permeabilization associated with early apoptosis, whereas YO‐PRO‐1/PI dual‐positive cells (orange fluorescence) indicate severe membrane compromise characteristic of late apoptosis, pyroptosis, and necroptosis [76, 77]. G6 displayed elevated populations and mean fluorescence intensity (MFI) of YO‐PRO‐1^+^/PI^+^ cells compared to other groups (Figure 4i and Figure S21). This necroptotic response was associated with elevated phosphorylation of RIPK3 and MLKL, as reflected by increased p‐RIPK3/RIPK3 and p‐MLKL/MLKL ratios (Figure 4j and Figure S18g–j). Multiple immunofluorescence staining was performed for executioner proteins GSDMD‐N (pyroptosis), c‐caspase‐3 (apoptosis), and p‐MLKL (necroptosis). CLSM revealed colocalization of all three markers within individual cells in G6, with higher mean MFI for each marker compared to other groups (Figure S22).

To validate the integrated nature of this cell death response, pharmacological inhibition studies were conducted using pathway‐specific inhibitors. Cells in G6 were incubated with caspase‐1 inhibitor VX‐765 against pyroptosis, pan‐caspase inhibitor Z‐VAD‐FMK against apoptosis, or RIPK3 inhibitor GSK‐872 against necroptosis, individually or in combination (Figure S23). Single‐pathway inhibition resulted in modest increases in cell viability from 29.74 ± 3.48% (G6 alone) to 36.84 ± 2.42% (G6+VX‐765), 39.06 ± 2.83% (G6+Z‐VAD‐FMK), and 35.45 ± 2.53% (G6+GSK‐872). Dual‐pathway inhibition increased cell viability to 61.91 ± 2.53% (G6+VX‐765+Z‐VAD‐FMK), 58.42 ± 2.81% (G6+VX‐765+GSK‐872), and 62.26 ± 2.67% (G6+Z‐VAD‐FMK +GSK‐872). Notably, simultaneous blockade of all three pathways substantially increased cell viability to 85.91 ± 3.60%. These results indicated that inhibition of individual pathways provided limited protection, while complete blockade achieved greater rescue, demonstrating coordinated function of these pathways. Collectively, these molecular and functional findings confirmed that TCPD nanodepot‐enabled sono‐thermal ablation triggered PANoptosis through concurrent activation of pyroptosis, apoptosis, and necroptosis pathways.

### 2.6 Convergent Multimodal Stress Facilitates Multisensor PANoptosome Assembly

The initiation of PANoptosis requires coordinated activation of upstream sensors that respond to damage‐associated signals generated during cellular stress [74, 77]. To evaluate the upstream mitochondrial events, mitochondrial ROS (mtROS) were quantified via MitoSOX Red/Mito‐Tracker dual staining (Figure 4k and Figure S24a). G6 exhibited elevated mtROS compared to moderate increases in G4 and G5. JC‐1 staining revealed the highest relative monomer‐to‐aggregate fluorescence ratio in G6, indicating mitochondrial membrane potential (ΔΨm) depolarization (Figure 4k and Figure S24b). Biological TEM provided ultrastructural validation of mitochondria, revealing widespread cristae dissolution, outer membrane rupture, and matrix content leakage in G6, contrasting with partial cristae damage in G4 and G5 (Figure 4l). Moreover, Western blot analyses demonstrated cleavage of long‐form OPA1 (L‐OPA1), Bcl‐2 downregulation, BAX activation, and cytosolic release of cytochrome c (Cyt c) in G6 (Figure 4m and Figure S25a‐d), consistent with mitochondrial outer membrane permeabilization (MOMP). The observed mitochondrial dysfunction aligns with established mechanisms of PANoptosis initiation. Elevated mtROS combined with GSH depletion likely contributes to ΔΨm depolarization and triggers L‐OPA1 cleavage, which disrupts cristae architecture and facilitates Cyt c mobilization into the intermembrane space [78, 79, 80]. The concurrent Bcl‐2 downregulation and BAX activation create conditions permissive for MOMP, and the resulting cytosolic release of Cyt c activates caspase‐3, contributing to the apoptotic component of PANoptosis.

In parallel, ER stress was evidenced by dilation of the ER lumen and fragmentation of its reticular architecture under TEM. This damage exceeded the capacity of the adaptive unfolded protein response, as demonstrated by phosphorylation of PERK and eIF2α, upregulation of ATF4, and a 6.5 fold elevation of CHOP in G6 (Figure 4m and Figure S25e–h). This ER damage resulted from the combined effects of MWA‐mediated protein denaturation [81], SDT‐derived ^1^O_2_‐induced oxidative impairment of protein folding [82, 83], and Tela‐induced ATP depletion. In comparison, G4 and G5 exhibited moderate activation of the PERK/eIF2α/ATF4/CHOP pathway. Critically, this unresolved ER stress synergized with mtROS to drive NLRP3 inflammasome assembly [76, 84]. Further Western blotting confirmed that NLRP3 expression was highest in G6, triggering the pyroptotic component of PANoptosis.

Alongside these ultrastructural alterations, mitochondrial damage facilitated the release of mtDNA into the cytosol [85, 86]. Quantitative PCR analysis of three mtDNA‐encoded genes (*D‐loop, COX1*, and *Nd1*) in cytosolic extracts demonstrated 9.7 fold, 8.6 fold, and 7.9 fold increases in G6 relative to G1, respectively (Figure 4n). To assess DNA oxidative damage, Alexa Fluor 488‐conjugated avidin binding to 8‐oxo‐dG was performed, revealing the highest 8‐oxo‐dG accumulation in G6 compared to other groups. Oxidative stress also extended to nuclear DNA, as evidenced by immunofluorescence staining for poly(ADP‐ribose) (PAR), which demonstrated substantial nuclear PAR accumulation in G6 (Figure S26). As the primary sensor for DNA damage, PARP synthesizes PAR polymer chains that recruit downstream repair machinery, and the observed PAR accumulation indicated persistent DNA fragmentation [87, 88, 89]. These nucleic acid damages resulted in elevated expression of AIM2 and ZBP1, cytosolic sensors that recognize aberrant nucleic acid structures and double‐stranded DNA [90, 91], as confirmed by Western blot in G6 (Figure 4o and Figure S27). Collectively, TCPD nanodepot‐empowered spatially complementary sono‐thermal ablation exacerbated intracellular nucleic acid oxidative damage, thereby activating damage sensors associated with PANoptosis initiation.

PARP hyperactivation drives substantial consumption of the intracellular NAD^+^ pool [92, 93]. While PARylation facilitates DNA repair under moderate stress, the extensive DNA damage converted this protective response into a metabolic burden. Consequently, sustained PAR synthesis resulted in severe NAD^+^ depletion [94, 95]. The disrupted glutaminolysis‐TCA axis further impaired the supply of α‐ketoglutarate and aspartate, precursors for NAD^+^ salvage synthesis [96, 97]. The NAD^+^ pool in G6 was therefore subjected to dual stress comprising accelerated enzymatic consumption and impaired metabolic regeneration (Figure 4n). NAD^+^ depletion has been shown to trigger PANoptosis by upregulating NLRC5, a sensor that contributes to PANoptosome assembly [44, 95]. Consistent with this mechanism, Western blot revealed a 3.4 fold upregulation of NLRC5 in G6 (Figure 4o and Figure S27). These multimodal cellular stresses collectively resulted in translational upregulation of key innate sensors, including ZBP1, AIM2, NLRP3, and NLRC5, thereby establishing an intracellular microenvironment permissive for PANoptosome assembly.

To determine whether the coordinated stress drives PANoptosome assembly, co‐immunoprecipitation (Co‐IP) assays targeting ASC were performed. Co‐IP analysis confirmed that G6 induced physical association of ASC with multiple PANoptosome components including NLRP3, AIM2, ZBP1, and Pyrin, as well as downstream effectors RIPK1, RIPK3, and caspase‐8 (Figure S28). These data demonstrate that subcellular stress promotes assembly of a multisensor PANoptosome complex on an ASC scaffold. To establish the functional dependency of this assembly, ASC knockdown experiments were performed. Western blot demonstrated that ASC silencing resulted in coordinated attenuation of all three PANoptosis executioners, including GSDMD‐N, c‐caspase‐3, and p‐MLKL (Figure S29), confirming that PANoptosome disruption simultaneously impairs pyroptosis, apoptosis, and necroptosis pathways. To further establish the causal relationship between upstream stress and PANoptosome assembly, functional rescue experiments were performed using the mitochondria‐targeted antioxidant MitoTEMPO. MitoSOX Red/Mito‐Tracker Green dual staining confirmed that MitoTEMPO effectively reduced mtROS levels, with relative MFI decreasing from 15.41 ± 1.11 in G6 to 5.69 ± 0.53 in the rescued group (Figure S30a,b). Consistent with mtROS reduction, Western blot demonstrated that MitoTEMPO significantly attenuated all three PANoptosis executioners, including GSDMD‐N, c‐caspase‐3, and p‐MLKL (Figure S30c–i). Cell viability assays further revealed that MitoTEMPO substantially rescued cell survival, with viability recovering from 29.42 ± 2.34% in G6 to 57.66 ± 1.44% in the rescued group (Figure S30j). These functional rescue data suggest that mitochondrial oxidative stress serves as a critical upstream driver of multisensor PANoptosome assembly and PANoptosis execution. Collectively, these findings demonstrate that TCPD‐empowered spatially complementary sono‐thermal ablation induces convergent multimodal stress, encompassing mitochondrial and ER dysfunction, DNA damage, and NAD^+^ depletion, leading to coordinated activation of multiple sensors and their assembly into a multisensor PANoptosome on an ASC scaffold, thereby executing PANoptosis (Figure 4p).

### 2.7 Immunogenic PANoptosis Coupled With STING Activation to Reprogram Innate Immunity

**FIGURE 5:**
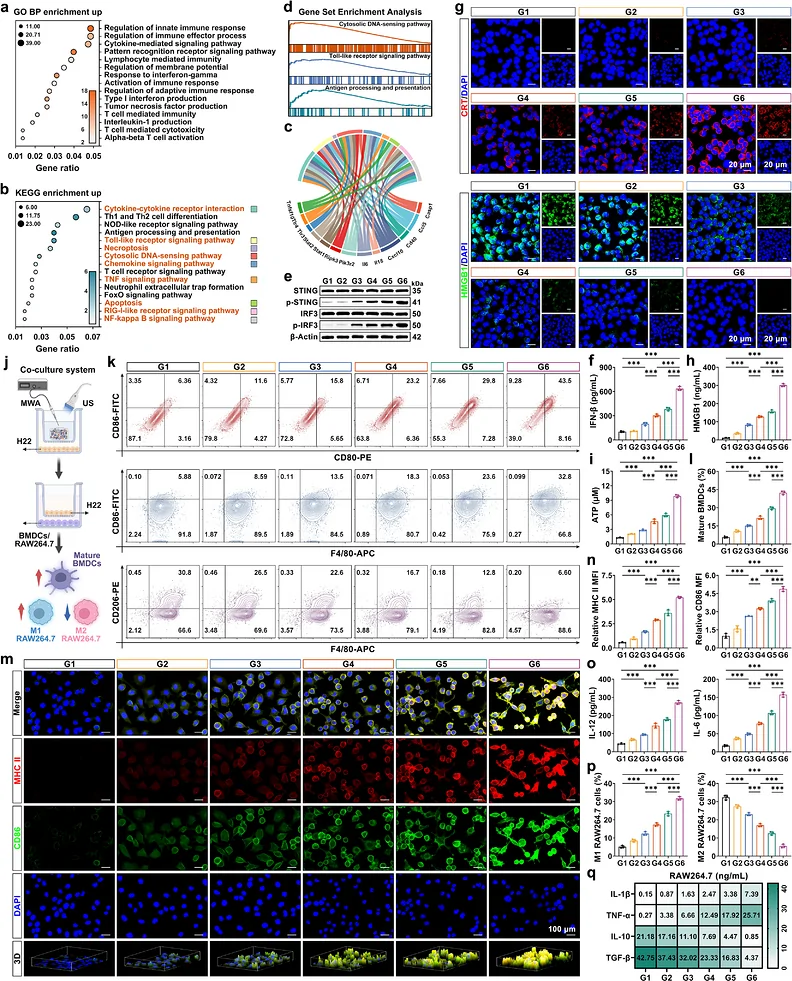
Immunogenic PANoptosis couples with STING Activation for DC maturation and macrophage phenotypic reprogramming. (a‐d) Gene Ontology (GO) enrichment analysis, Kyoto Encyclopedia of Genes and Genomes (KEGG) enrichment analysis, Chord plot, and GSEA of immune‐related DEGs. (e) Western blotting for STING, p‐STING, IRF3, and p‐IRF3 protein expression. (f) Quantification of IFN‐β concentration by an ELISA kit. (g) Representative immunofluorescence staining images and of CRT and HMGB1. (h) Quantification of HMGB1 concentration in supernatant by an ELISA kit. (i) Quantification of ATP content in supernatant by an Enhanced ATP Assay Kit. (j) Schematic diagram of co‐culture model directing BMDC maturation and macrophage phenotypic reprogramming. (k) Flow cytometry images of mature CD11c^+^CD80^+^CD86^+^ BMDCs (gated by CD11c^+^) and F4/80^+^CD86^+^ and F4/80^+^CD206^+^ RAW264.7 cells. (l) Statistical analyses of mature CD11c^+^CD80^+^CD86^+^ BMDCs (gated by CD11c^+^). (m‐n) Representative dual immunofluorescence staining images and statistical analyses of MHC‐II/CD86 in BMDCs. (o) ELISA for cytokine levels of IL‐12 and IL‐6 secreted by BMDCs. (p) Statistical analyses of F4/80^+^CD86^+^ M1 and F4/80^+^CD206^+^ M2 RAW264.7 cells. (q) ELISA for cytokine levels of IL‐1β, TNF‐α, IL‐10, and TGF‐β secreted by RAW264.7 cells. Statistical analysis data represent n = 3 biologically independent experiments and are presented as mean ± standard deviation. Statistical comparisons were performed using one‐way ANOVA with Tukey's multiple comparisons test. Scale bars: 20 and 100 µm.

Beyond cell death pathways, GO BP and KEGG enrichment analysis of upregulated DEGs revealed the activation of diverse innate immune networks, including regulation of innate immune response, pattern recognition receptor signaling pathway, cytokine‐cytokine receptor interaction, antigen processing and presentation, Toll‐like receptor signaling, and the cytosolic DNA‐sensing pathway (Figure 5a,b). Chord plot and GSEA analysis corroborated the enrichment of DEGs involved in antigen presentation, the cytosolic DNA‐sensing pathway, the RIG‐I‐like receptor signaling pathway, and the Toll‐like receptor signaling pathway (Figure 5c,d). The cGAS‐STING signaling axis represents the canonical effector module within the cytosolic DNA‐sensing network [98]. Mechanistically, the cytosolic leakage of damaged DNA described above provides the substrate to engage cGAS, thereby initiating downstream STING‐dependent signaling. Subsequent Western blot demonstrated markedly enhanced phosphorylation of core components, specifically STING and IRF3, compared to G1, reaching maximum activation in G6 (Figure 5e and Figure S31). This cascade activation functionally translated into IFN‐β secretion, measured at 636.59 ± 25.43 pg/mL in G6, which subsequently drove a type I interferon response characterized by transcriptional upregulation of interferon‐stimulated genes (Figure 5f). These results confirm that TCPD nanodepot‐empowered spatially complementary sono‐thermal ablation effectively transduces therapy‐induced genotoxicity into the propagation of innate immune signals.

PANoptosis constitutes a highly immunogenic cell death (ICD) characterized by the emission of DAMPs, specifically the surface translocation of calreticulin (CRT), the extracellular release of nuclear high mobility group box 1 (HMGB1), and the secretion of ATP [62, 90]. Immunofluorescence analysis of these markers revealed negligible CRT membrane exposure and HMGB1 extracellular release in G4 and G5, whereas G6 exhibited extensive CRT surface exposure and profound HMGB1 redistribution (Figure 5g and Figure S32). Biochemical quantification of supernatant mediators confirmed this trend, with extracellular HMGB1 concentration at 301.26 ± 8.13 ng/mL and ATP secretion at 9.86 ± 0.30 µM in G6 (Figure 5h,i). These results establish that the TCPD nanodepot‐empowered spatially complementary sono‐thermal ablation induces an ICD profile concurrent with tumor PANoptosis.

The release of DAMPs and the induction of a type I interferon response foster a pro‐inflammatory milieu capable of promoting DC maturation and reprogramming the immunosuppressive TME [24, 99, 100, 101]. To evaluate nanodepot‐mediated immunomodulation consequences, H22 cells treated with different interventions were co‐cultured with bone marrow‐derived DCs (BMDCs) or RAW264.7 macrophages using a Transwell system (Figure 5j). Flow cytometry confirmed an increased population of CD11c^+^CD80^+^CD86^+^ mature BMDCs in G4 and G5. This maturation was further enhanced in G6, achieving 2.0 fold and 1.4 fold increases relative to G4 and G5, respectively (Figure 5k, l). Further immunofluorescence analysis corroborated the upregulation of MHC‐II and CD86 alongside morphological changes indicative of enhanced antigen‐presenting capacity (Figure 5m,n). This cellular maturation effectively coincided with increased secretion of immunoregulatory cytokines IL‐6, IL‐12, IFN‐γ, and TNF‐α (Figure 5o and Figure S33).

Modulating the intra‐tumoral macrophage phenotype contributes to anti‐tumor immunity [102, 103]. Analysis confirmed that G6 increased the proportion of F4/80^+^CD86^+^ M1‐polarized macrophages to 31.77 ± 0.96% and elevated expression of the M1‐associated marker iNOS (Figure 5p and Figure S34). To model the M2‐dominant immunosuppressive conditions of solid tumors, RAW264.7 cells polarized toward an M2 phenotype via IL‐4 and IL‐13 supplementation were used [104, 105, 106]. While partial repolarization was observed in other groups, G6 reduced the F4/80^+^CD206^+^ M2 population to 5.53 ± 0.97% (Figure 5p). Concordantly, extracellular cytokine quantification showed a pro‐inflammatory shift characterized by elevated IL‐1β and TNF‐α levels with reduced IL‐10 and TGF‐β (Figure 5q). Collectively, these in vitro profiles demonstrate that TCPD‐empowered spatially complementary sono‐thermal ablation triggers immunogenic PANoptosis and STING activation, thereby modulating local immune populations toward an immunostimulatory state.

### 2.8 Bimodal TCPD Nanodepot‐Empowered Sono‐Thermal Ablation Drives PANoptosis and TME Remodeling to Eradicate Large Tumor

**FIGURE 6:**
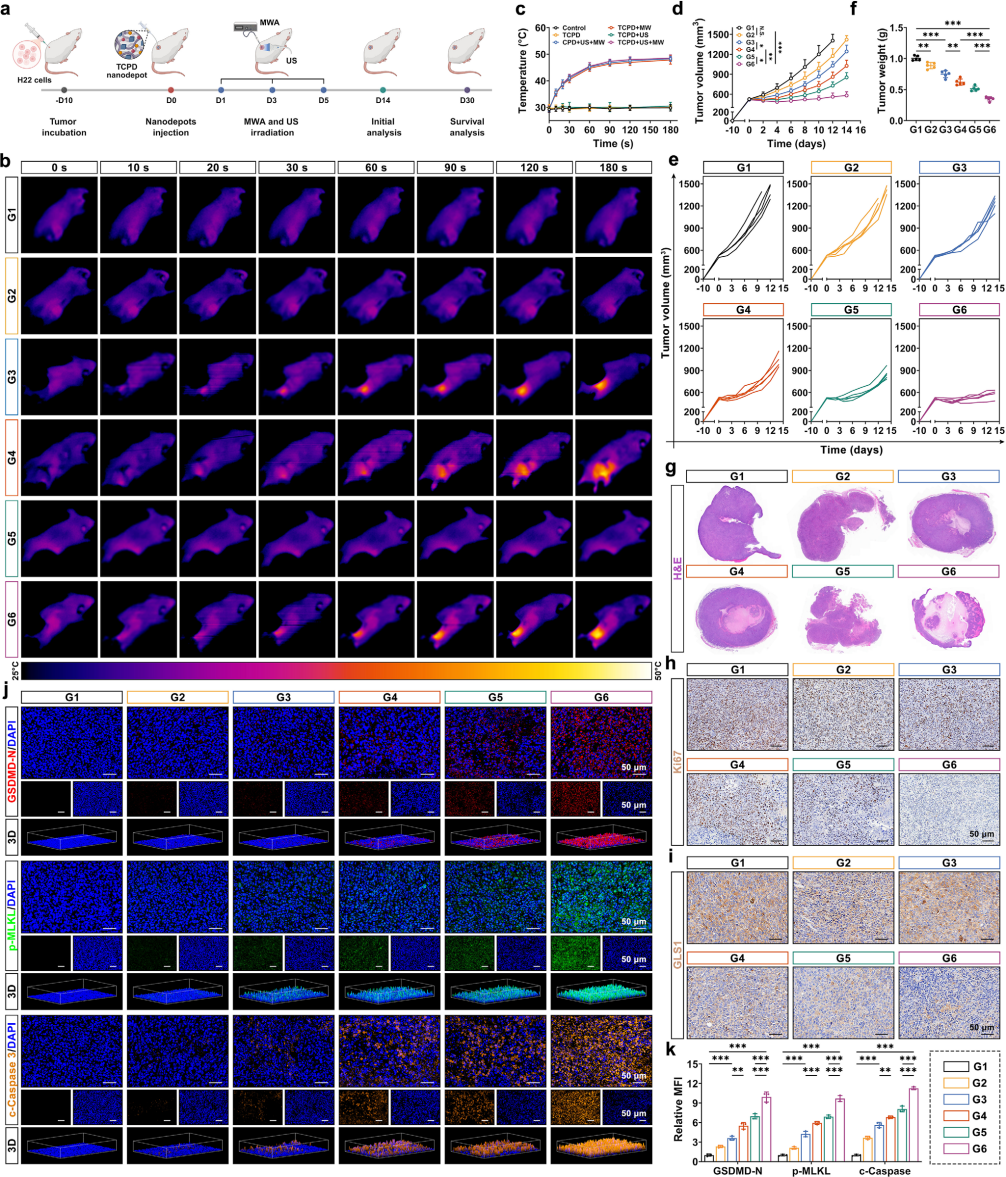
In vivo glutaminolysis blockade TCPD nanodepot‐empowered spatially complementary sono‐thermal ablation against large tumor. (a) Mouse large tumor model timeline flowchart. (b) Representative infrared thermal images of tumor‐bearing mice under different treatments. (c) Quantified tumor‐surface temperature profiles under different treatments. (d) Mean tumor growth curves (days 0–12 for G1‐G6 groups; G1 group euthanized on day 14 at humane endpoint). (e) Individual tumor volume growth trajectories. (f) Terminal tumor weights. (g‐i) Representative staining images of H&E, Ki67 immunohistochemistry, and GLS1 immunohistochemistry in tumor tissues. (j‐k) Representative immunofluorescence staining images and statistical analyses of GSDMD‐N, p‐MLKL, and c‐Caspase 3 in tumor tissues. Data in (d) and (e) represent n = 5 and in (c) and (k) represent n = 3 independent biologically mice per group, and are presented as mean ± standard deviation. Statistical comparisons in (d) were performed REML linear mixed‐effects model with Geisser‐Greenhouse correction and Tukey's multiple comparisons test, and in (k) using one‐way ANOVA with Tukey's multiple comparisons test. Scale bars: 50 µm.

The in vivo therapeutic efficacy was evaluated utilizing a large tumor model in H22 tumor‐bearing BALB/c mice randomly assigned to six intervention cohorts including G1 (Control), G2 (TCPD), G3 (CPD+US+MW), G4 (TCPD+MW), G5 (TCPD+US), and G6 (TCPD+US+MW) (Figure 6a). To assess in vivo MW thermal conversion efficiency, real‐time infrared thermographic imaging was performed on tumor‐bearing mice over the course of the treatment. In G3, G4, and G6 receiving TCPD or CPD nanodepots alongside MW antenna implantation, local tumor temperatures increased rapidly, exceeding the hyperthermia threshold of 45°C within 90 s and reaching 48.3 ± 1.0°C, 47.8 ± 1.6°C, and 48.5 ± 1.2°C at 3 min, respectively (Figure 6b,c). In contrast, groups without MW antenna implantation maintained stable baseline temperatures of approximately 30 °C throughout the monitoring period. To evaluate thermal confinement, peritumoral temperature remained below 37°C throughout the procedure, and hematoxylin and eosin (H&E) staining of peritumoral tissues (adjacent skin and muscle) revealed intact architectures with no signs of thermal damage in G6 (Figure S35). Following 14 days of observation, mice were sacrificed for efficacy analysis. All mice in G1 and two mice in G2 reached the ethical tumor burden limit and were humanely euthanized before day 14. Body weight showed no significant differences between groups, indicating negligible systemic toxicity (Figure S36a). Tumor volume and weight analyses demonstrated that while G4 and G5 partially delayed tumor growth, G6 most effectively suppressed tumor progression (Figure 6d–f). A 30‐day Kaplan‐Meier survival analysis revealed that all mice in G1 died within 20 days due to tumor burden, whereas 60% of mice in G6 survived to the study endpoint (Figure S36b). Moreover, H&E staining of major organs revealed no appreciable histopathological abnormalities, thereby confirming the satisfactory biosafety profile of TCPD nanodepots (Figure S37).

Encouraged by the favorable therapeutic performance, the in vivo biodegradation behavior, metabolic clearance pathways, and systemic biocompatibility of subcutaneously implanted TCPD nanodepots were further evaluated in healthy mice. Free Ce6 was rapidly eliminated within 3 days, whereas TCPD nanodepots maintained a pronounced local fluorescence signal for 14 days with weak residual signals detectable on day 28 (Figure S38a), confirming prolonged retention at the target site. Histological analysis of subcutaneously implanted nanodepots demonstrated progressive degradation, accompanied by gradual host cellular infiltration without tissue necrosis (Figure S38b). By day 35, TCPD nanodepots underwent near‐complete biosorption, exhibiting favorable local tissue biocompatibility. Serum ELISA quantification revealed a minor, transient increase in IL‐6 and TNF‐α levels during the initial post‐implantation phase that returned to baseline by day 7 (Figure S38c,d), confirming that TCPD nanodepots did not induce persistent inflammation. Concurrently, Cs‐PB disassembled into cesium and iron species upon TCPD nanodepot biodegradation, with local elemental content decreasing by 94.0% for cesium and 85.6% for iron over 28 days (Figure S39a). Cesium exhibited transient tissue distribution peaking around day 7 and was predominantly eliminated via renal excretion, achieving 83.70 ± 2.45% urinary and 9.82 ± 2.98% fecal recovery by day 28, corresponding to nearly complete clearance (Figure S39b,d). Meanwhile, iron entered the endogenous iron homeostasis system, showing a later distribution peak around day 14 with modest hepatosplenic retention consistent with transferrin‐mediated transport and reticuloendothelial storage, followed by gradual dual clearance with cumulative recoveries of 30.16 ± 2.74% in urine and 39.23 ± 2.08% in feces by day 28 (Figure S39c, e). Consistent with these clearance profiles, comprehensive hepatic, renal, and hematological assessments performed on days 1, 7, 14, and 28 confirmed that liver and kidney function markers remained within physiological reference ranges and that erythroid and platelet parameters were unperturbed (Figure S40). Collectively, these findings demonstrate that TCPD nanodepots undergo multi‐component degradation and safe physiological clearance without eliciting local or systemic toxicity.

To evaluate the spatial distribution of tumoricidal effect at the histopathological level, H&E staining was performed on excised tumor sections (Figure 6g). G1 exhibited densely packed viable tumor cells characterized by enlarged hyperchromatic nuclei, conspicuous nucleoli, and abundant mitotic figures with minimal necrosis. G2 displayed increased necrotic areas appearing as multifocal patches among viable tumor tissue. G4 and G5 showed distinct spatial necrosis patterns. G4 featured a central zone of coagulative necrosis surrounded by a viable peripheral rim, consistent with MWA penetration and thermal attenuation toward tumor margins. G5 demonstrated necrotic regions localized to outer tumor layers with a viable central core, reflecting depth‐dependent attenuation of SDT. Although these two modalities exhibit spatial complementarity in principle, G3 yielded only fragmented necrosis with substantial viable tissue remaining. Notably, G6 showed extensive necrosis extending from the outer margin to the inner core, with residual cells displaying karyorrhexis, cytoplasmic vacuolization, and structural disintegration. The superior efficacy of G6 can be attributed to Tela‐mediated metabolic reprogramming, which sensitizes tumor cells to physical stimuli and lowers the threshold for cell death. The integration of metabolic sensitization with dual physical modalities addresses both spatial coverage and cellular resistance, achieving more complete tumor destruction. Ki67 immunohistochemistry revealed reduced tumor cell proliferation in G6 compared to other groups (Figure 6h). Tumor sections from other groups retained Ki67‐positive nuclei across histological fields, indicating residual proliferative activity.

To validate in vivo metabolic modulation and PANoptosis execution within the TME, tumor sections were subjected to immunohistochemistry and immunofluorescence staining. Immunohistochemical analysis of GLS1 revealed differential expression patterns across treatment groups (Figure 6i and Figure S36c). Tissues from G1 and G3 exhibited strong GLS1 expression. G2 displayed reduced GLS1 positivity, consistent with Tela release from TCPD nanodepots. G4 and G5 showed further reduction in GLS1 expression following physical stimulation. G6 demonstrated the lowest GLS1 expression, indicating substantial glutaminolysis suppression. Immunofluorescence analysis of GSDMD‐N, c‐caspase‐3, and p‐MLKL revealed distinct expression patterns across groups (Figure 6j). While individual modalities induced localized expression of specific markers, G6 showed the highest expression of all three markers within overlapping tumor regions (Figure 6k). Additionally, G6 exhibited enhanced CRT exposure within tumor tissues compared to other groups (Figure S41). These findings suggest that TCPD nanodepot‐empowered spatially complementary sono‐thermal ablation can induce immunogenic PANoptosis, which may help circumvent inherent resistance and improve eradication of large tumor burdens.

**FIGURE 7:**
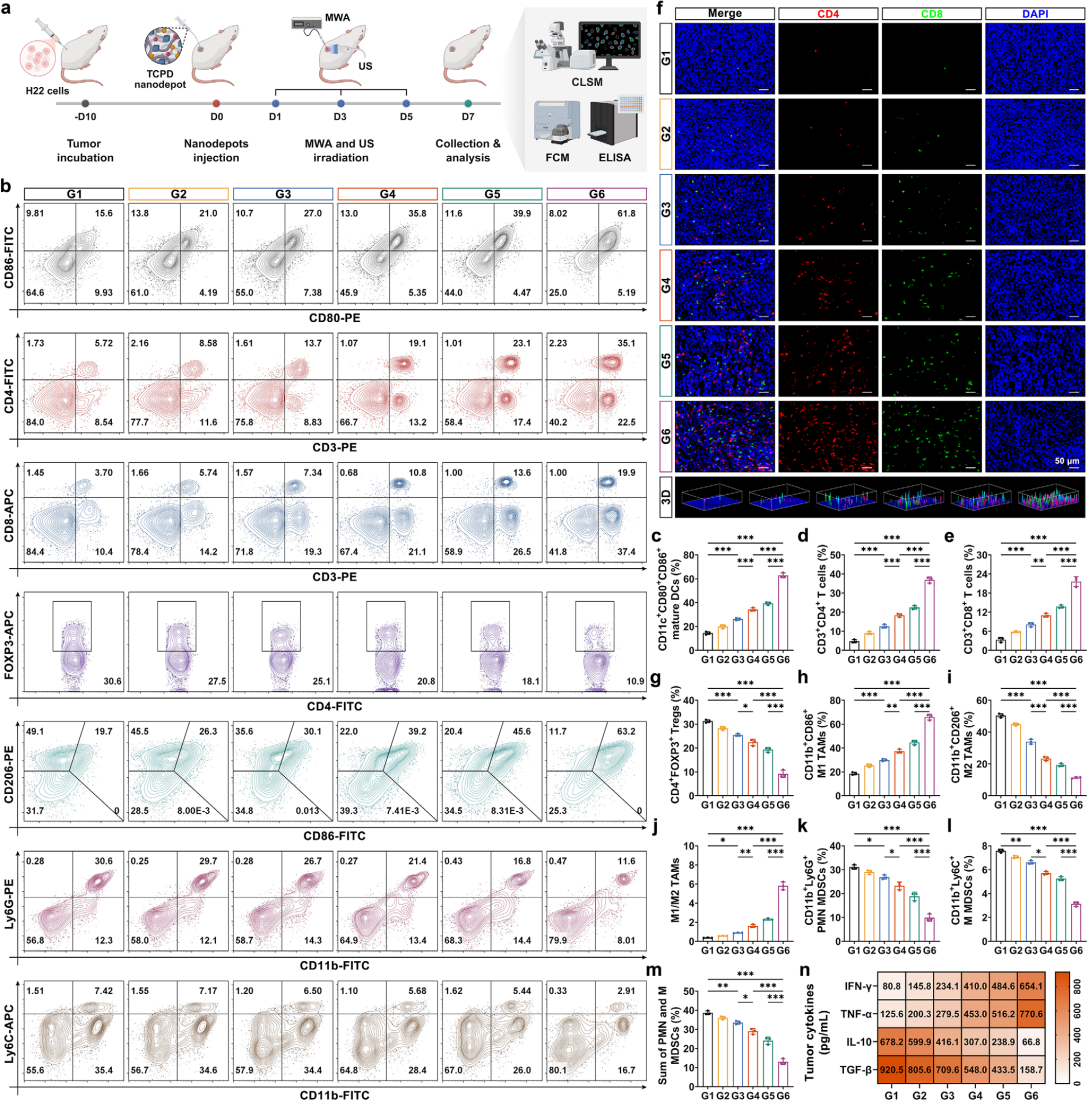
TCPD nanodepot‐empowered spatially complementary sono‐thermal ablation remodels immune microenvironment. (a) Mouse large tumor model timeline flowchart. (b) Flow cytometry images of CD11c^+^CD80^+^CD86^+^ mature DCs (gated on CD11c^+^) in tumor draining lymph nodes, CD3^+^CD4^+^ and CD3^+^CD8^+^ T cells, CD3^+^CD4^+^FOXP3^+^ Tregs (gated on CD3^+^CD4^+^), CD11b^+^CD86^+^ and CD11b^+^CD206^+^ TAMs (gated on CD11b^+^), and CD11b^+^Ly6G^+^ PMN‐ and CD11b^+^Ly6C^+^ M‐MDSCs in tumor tissues. (c‐e) Statistical analyses of CD11c^+^CD80^+^CD86^+^ mature DCs (gated on CD11c^+^) in tumor draining lymph nodes, CD3^+^CD4^+^ and CD3^+^CD8^+^ T cells in tumor tissues. (f) Representative dual immunofluorescence staining images of CD4/CD8 in tumor tissues. (g‐m) Statistical analyses of CD3^+^CD4^+^FOXP3^+^ Tregs (gated on CD3^+^CD4^+^), CD11b^+^CD86^+^ and CD11b^+^CD206^+^ TAMs (gated on CD11b^+^), and CD11b^+^Ly6G^+^ PMN and CD11b^+^Ly6C^+^ M MDSCs in tumor tissues. (n) Quantitative analyses of IFN‐γ, TNF‐α, IL‐10, and TGF‐β in tumor tissues. Data represent n = 3 biologically independent mice per group and are presented as mean ± standard deviation. Statistical comparisons were performed using one‐way ANOVA with Tukey's multiple comparisons test. Scale bars: 50 µm.

To further elucidate the underlying immunological effects, a murine subcutaneous large tumor model was re‐established with tumor‐draining lymph nodes (TDLNs) and tumor tissues harvested on day 7 post‐treatment for comprehensive profiling (Figure 7a). Flow cytometric analysis of TDLNs revealed that G6 exhibited the highest proportion of CD11c^+^CD80^+^CD86^+^ mature DCs at 62.85 ± 2.00% (gated on CD45^+^CD11c^+^), compared to G4 and G5 (Figure 7b,c). To assess T cell infiltration independent of tumor cellularity changes following treatment, flow cytometric profiling of tumor tissues employed a hierarchical gating strategy in which CD45^+^ leukocytes were first identified to exclude tumor cells and stromal components. Within the CD45^+^ compartment, G6 exhibited an elevated frequency of total CD3^+^ T cells at 59.17 ± 2.54%, compared to G4 (31.66 ± 0.64%) and G5 (39.03 ± 1.19%) (Figure S42a). Further subset analysis demonstrated that CD3^+^CD4^+^ and CD3^+^CD8^+^ T cells within the CD45^+^ population in G6 reached 36.90 ± 1.57% and 21.64 ± 1.53% (gated on CD45^+^), respectively (Figure 7d,e). Moreover, CD4/CD8 dual immunofluorescence staining revealed that both CD4^+^ and CD8^+^ T cell densities within viable tumor parenchyma were elevated in the G6 group compared to other groups, with preferential localization at the invasive margin and peritumoral stroma (Figure 7f and Figure S42b, c). This spatial distribution pattern suggests lymphocyte recruitment rather than passive accumulation. Further analysis of the T‐cell compartment revealed that CD3^+^CD4^+^FOXP3^+^ Tregs in G6 were reduced to 9.14 ± 1.53% (gated on CD3^+^CD4^+^), a level below that observed in other groups (Figure 7g). Profiling of TAMs showed a M2 macrophage‐dominated baseline in G1, whereas G6 induced antitumoral repolarization. Specifically, CD86^+^ TAMs reached 65.93 ± 2.39% with a concurrent reduction of CD206^+^ TAMs to 11.38 ± 0.46% (gated on CD45^+^F4/80^+^), yielding an ratio of 5.80 ± 0.41 (Figure 7h–j). Intratumoral frequencies of CD11b^+^Ly6G^+^ PMN and CD11b^+^Ly6C^+^ M MDSCs in G6 decreased to 9.91 ± 1.47% and 3.13 ± 0.19% (gated on CD45^+^), respectively, remaining lower than those in other groups (Figure 7k–m). Flow cytometric antibodies are detailed in Table S5. Quantitative ELISA analysis detected elevated levels of pro‐inflammatory cytokines IFN‐γ and TNF‐α alongside reduced levels of immunosuppressive factors IL‐10 and TGF‐β in G6 (Figure 7n and Table S6). Collectively, these results suggest that TCPD nanodepot‐empowered spatially complementary sono‐thermal ablation can induce an immunomodulatory effect characterized by enhanced DC maturation, TAM phenotypic reprogramming toward M1 polarization, and depletion of Tregs and MDSCs. This integrated process may help convert the tolerogenic TME into an immune‐active niche, potentially establishing a coordinated response that bridges innate immune sensing with adaptive antitumor immunity.

### 2.9 Synergizing With ICIs to Potentiate Systemic Antitumor Immunity and Durable Memory

**FIGURE 8:**
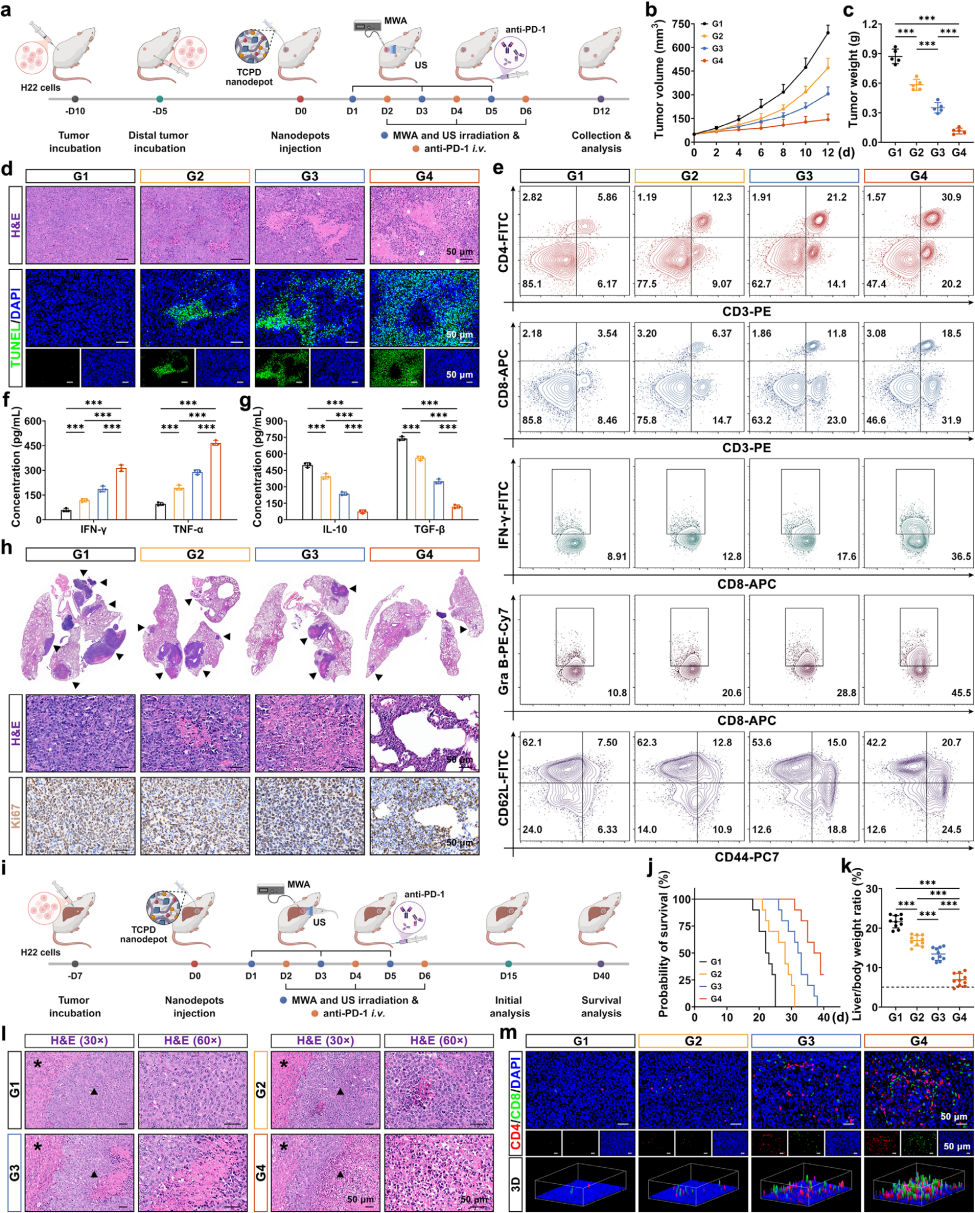
Combination with ICIs potentiates systemic antitumor immunity and orthotopic liver cancer suppression. (a) Mouse bilateral tumor model timeline flowchart. (b‐c) Contralateral tumor volume curves and tumor weights. (d) H&E and Ki67 immunohistochemistry staining of tumor tissues. (e) Flow cytometry images of CD3^+^CD4^+^ and CD3^+^CD8^+^ T cells, and IFN‐γ^+^ and granzyme B^+^ CTLs (gated on CD3^+^CD8^+^) in contralateral tumor tissues, and central memory T cells (T_CM_) and effector memory T cells (T_EM_) cells in spleens (gated on CD3^+^CD8^+^). (f‐g) Quantitative analyses of IFN‐γ, TNF‐α, IL‐10, and TGF‐β in contralateral tumor tissues. (h) H&E and Ki67 immunohistochemistry staining of lungs. (i) Mouse orthotopic liver cancer model timeline flowchart. (j‐k) Liver to body weight ratio and survival analysis of mice during treatment. (l) Images of H&E staining of orthotopic liver tumors, triangles represent normal liver tissue and asterisks represent tumor tissue. (m) Representative dual immunofluorescence staining images of CD4/CD8 in orthotopic liver tumors. Data in (b) and (c) represent n = 5, in (f) and (g) represent n = 3, and in (j) and (k) represent n = 10 biologically independent mice per group, and are presented as mean ± standard deviation. Statistical comparisons were performed using one‐way ANOVA with Tukey's multiple comparisons test. Scale bars: 50 µm.

The cancer‐immunity cycle is frequently disrupted by PD‐1/PD‐L1 checkpoint‐mediated immunosuppression, which attenuates CTL function within the TME [107]. Immune checkpoint blockade can reverse CTL exhaustion and restore antigen‐specific cytotoxicity, representing a viable strategy for maintaining durable antitumor immunity. To investigate whether the TCPD nanodepot combined with MWA and US irradiation could synergize with anti‐PD‐1 therapy to suppress distant metastasis and elicit long‐term immune memory, a mouse bilateral tumor model was constructed (Figure 8a). Tumor‐bearing mice were randomized into four groups: G1 (Control), G2 (anti‐PD‐1), G3 (TCPD+US+MW), and G4 (TCPD+US+MW+anti‐PD‐1). Endpoint analysis on day 12 demonstrated delayed contralateral tumor progression across all treatment groups, with the most pronounced suppression observed in G4 (Figure 8b,c). H&E staining corroborated these macroscopic findings. Contralateral tumor tissues in G1 retained malignant histological features, including structural disorganization and cellular pleomorphism, without apparent necrosis. In contrast, G4 exhibited pronounced abscopal tumoricidal effects with the highest necrotic burden. These morphological alterations were supported by TUNEL staining, which revealed a significantly elevated apoptotic index within distant tumors of G4 (Figure 8d).

To characterize the systemic abscopal immune response, flow cytometry was performed to quantitatively profile immune cell populations in untreated contralateral tumors. Immune profiling revealed increased immune infiltration in G4, with CD3^+^CD4^+^ and CD3^+^CD8^+^ T cell populations rising to 32.29 ± 1.25% and 18.98 ± 1.03%, respectively (Figure 8e and Figure S43a,b). The functional status of these infiltrating CTLs was enhanced, as frequencies of CD3^+^CD8^+^IFN‐γ^+^ and CD3^+^CD8^+^Granzyme B^+^ CTLs reached 38.45 ± 1.77% and 47.26 ± 1.57% (gated on CD3^+^CD8^+^) in G4, exceeding those in other groups (Figure S43c, d). Concurrently, immunosuppressive features of the TME were attenuated. Although G2 and G3 exhibited partial reductions in Tregs relative to G1, G4 demonstrated the most pronounced suppression, reducing Treg fraction to 6.26 ± 1.99% (Figure S44a, b). This relief of immune tolerance was accompanied by a shift in TAM polarization toward the tumoricidal CD86^+^ phenotype and reductions in both CD11b^+^Ly6G^+^ PMN and CD11b^+^Ly6C^+^ M MDSCs (Figure S44c–g). Splenic memory T cell analysis confirmed the establishment of systemic immune memory. In G4, populations of CD3^+^CD8^+^CD44^+^CD62L^+^ central memory T cells (T_CM_) and CD3^+^CD8^+^CD44^+^CD62L^−^ effector memory T cells (T_EM_) expanded to 22.76 ± 1.79% and 25.58 ± 1.72% (gated on CD3^+^CD8^+^), respectively (Figure 8e and Figure S43e, f). ELISA‐based cytokine profiling within contralateral tumors corroborated these cellular shifts, demonstrating elevated secretion of immunostimulatory factors alongside reduced levels of immunosuppressive cytokines (Figure 8f,g).

Concurrently, a mouse lung metastasis model was established to evaluate the capacity of this therapeutic strategy to suppress hematogenous dissemination and prevent metastatic colonization (Figure S45). Lung tissues were harvested for histological analysis. H&E and Ki67 immunohistochemical staining revealed that G4 exhibited a reduction in pulmonary metastatic burden. Specifically, metastatic lesions in G4 displayed reduced cellular atypia and suppressed proliferative activity, as evidenced by a decrease in the proportion of Ki67‐positive tumor cells (Figure 8h). These findings collectively indicate that the combined regimen attenuates pulmonary metastatic progression. The integrated TCPD+US+MW+anti‐PD‐1 strategy enhanced systemic antitumor immune response and engaged multiple steps of the cancer‐immunity cycle. This was characterized by a sequence of immunological events including tumor‐associated antigen release, immune priming, TME remodeling, and CTL activation, which contributed to establishment of antitumor immune memory and sustained tumor control.

### 2.10 Synergizing with ICIs for Orthotopic Liver Cancer Suppression

To evaluate the clinical translatability of the proposed therapeutic strategy, an orthotopic liver cancer model was subjected to treatment protocols mirroring subcutaneous investigations (Figure 8i). This multimodal regimen incorporated TCPD nanodepots with MWA, US irradiation, and intravenous anti‐PD‐1 administration. Kaplan‐Meier survival analysis demonstrated that while all mice in G1 died within 25 days due to tumor progression, 30% of animals receiving the combined therapy survived beyond day 40 (Figure 8j). Assessment of macroscopic tumor burden on day 15 demonstrated tumor growth suppression, as G1 exhibited a liver‐to‐body weight ratio of 21.42 ± 1.98% compared to a reduced ratio of 6.93 ± 1.58% in G4 (Figure 8k). Histopathological evaluation by H&E staining corroborated these findings, revealing more extensive architectural disruption and confluent tumor necrosis in G4 relative to other groups (Figure 8l). Furthermore, CD4/CD8 dual immunofluorescence staining demonstrated that the multimodal regimen induced higher intratumoral infiltration of both CD4^+^ and CD8^+^ T cells in G4 (Figure 8m). Collectively, these results indicate that integrating TCPD nanodepot‐empowered spatially complementary sono‐thermal ablation and ICIs suppresses tumor progression and prolongs survival in a clinically relevant orthotopic liver cancer model.

## 3 Conclusions

In summary, we engineered an injectable dual‐crosslinked TCPD nanodepot that serves as a bimodal‐responsive reservoir for sustained release of GLS inhibitor Tela, sonosensitizer Ce6, and MW thermal converter Cs‐PB. Locally administered under MWA and US irradiation, the TCPD nanodepot drives spatially complementary sono‐thermal ablation empowered by glutaminolysis‐TCA cycle axis blockade. Mechanistically, this bimodal‐responsive nanodepot simultaneously triggers multimodal subcellular stress, including mitochondrial/ER dysfunction, genotoxicity, and NAD^+^ depletion. These organelle‐level perturbations, together with released DNA fragments, converge to facilitate multisensor PANoptosome assembly, thereby promoting immunogenic PANoptosis and activating the cGAS‐STING pathway. Consequently, exposure to DAMPs and production of immunoregulatory cytokines enhance tumor immunogenicity and remodel the immunosuppressive TME by promoting DC maturation, reprogramming myeloid populations, and facilitating T lymphocyte infiltration. Integration with anti‐PD‐1 therapy further reinvigorates CTL function and establishes antitumor immune memory to effectively suppress tumor progression and metastasis, advancing a multimodal therapeutic strategy for large HCC.

## Author Contributions

H.Z. oversaw all research; D.W., Y.L., and J.Q. designed the experiments; Y.L., S.W., X.Z., Y.C., P.S., H.Y., and X. W. performed the experiments; all authors analyzed and interpreted the data; Y.L., J.Q., D.W., and H.Z. wrote and revised the manuscript; all authors reviewed and edited the paper.

## Funding

The study was supported by the National Natural Science Foundation of China (82402408, 82072039, 82372067), the National Key R&D Program of China (No. 2023YFC2413500), Jiangsu Provincial Medical Innovation Center (CXZX202219), The Natural Science Foundation of Jiangsu Province (BG2024007), and SEU Innovation Capability Enhancement Plan (CXJH SEU 25048). The funding sources had no role in the writing of the report or the decision to submit the paper for publication.

## Ethics Statement

All animal experiments were granted approval by the Experimental Animal Care & Welfare Committee of Southeast University (20251010002).

## Conflicts of Interest

The authors declare no conflicts of interest.

## Supporting information




**Supporting File**: advs77933‐sup‐0001‐SuppMat1.docx.

## Data Availability

The data that support the findings of this study are available on request from the corresponding author. The data are not publicly available due to privacy or ethical restrictions.
