# Supplementary material for: Glutaminolysis Blockade‐Empowered Bimodal Nanodepot for Spatially Complementary Sono‐Thermal Ablation via PANoptosis and STING Activation Against Large Tumors
Source: Adv Sci (Weinh). 2026 Sep 27:e77933. Online ahead of print. doi: 10.1002/advs.77933 (PMC13616403; doi:10.1002/advs.77933)
Supplement: Supplementary file 1 [file ADVS-9999-e77933-s001.docx]

Supporting information

**Glutaminolysis Blockade-Empowered Bimodal Nanodepot for Spatially Complementary Sono-Thermal Ablation via PANoptosis and STING Activation against Large Tumors**

Yi-Ming Liu ^1, #^, Juan Qin ^1, #^, Si-Yuan Wu ^1^, Xue-Zhen Zhu ^1^, Yun-Fei Cai ^1^, Pei-Yao Sun ^1^, Hao Yang ^1^, Xing-Yue Wang ^1^, Shu Li ^2^, Duo Wang ^1, *^, and Hai-Dong Zhu ^1, *^

^1^ Center of Interventional Radiology and Vascular Surgery, Department of Radiology, Zhongda Hospital, Medical School, Southeast University, 87 Dingjiaqiao Road, Nanjing 210009, China.

^2^ The Third Affiliated Hospital of Zhengzhou University, Zhengzhou 450052, China.

^#^ Yiming Liu and Juan Qin contributed equally to this work.

* **Corresponding authors**

*E-mail addresses:* wangduo2022@126.com (D. Wang), zhuhaidong9509@163.com (H.D. Zhu)

**Supplementary Materials and Methods**

***1. Materials and cell lines***

Sodium hyaluronate (HA; H909938) and dopamine hydrochloride (DA; D756911) was provided by Shanghai McLean Biochemical Technology Co., Ltd. (Shanghai, China). N-Ethyl-N′-(3-dimethylaminopropyl)carbodiimide (EDC; 39391), N-Hydroxysuccinimide (NHS; 130672), Cetyltrimethylammonium bromide (CTAB; 219374), 2-methylimidazole (2-MeIM; M50850), and ethyl alcohol (459836) were sourced from Merck KGaA (Darmstadt, Germany). Cobalt nitrate hexahydrate [Co(NO₃)₂.6H₂O] and copper nitrate trihydrate [Cu(NO₃)₂.3H₂O] were procured from Sinopharm Chemical Reagent Co., Ltd. Chlorin e6 (Ce6; HY-13594) and glutaminase inhibitor Telaglenastat (Tela; HY-12248) was obtained from MedChemExpress Biotechnology LLC (New Jersey, USA). Mouse H22 and Hepa1-6 liver cancer cells were obtained from the Shanghai Cell Center (Chinese Academy of Sciences). Dulbecco’s modified Eagle’s medium (DMEM; 11965118), fetal bovine serum (FBS; A5256701), penicillin-streptomycin (15140122), and trypsin-EDTA (25200056) were supplied by Thermo Fisher Scientific Inc. (Massachusetts, USA). The Live and Dead Cell Double Staining Kit (KTA1001) and Cell Counting Kit-8 (CCK-8; KTA1020) were sourced from Abbkine Scientific Co., Ltd. (Wuhan, China). The Annexin V-FITC/Propidium iodide (PI) Apoptosis Kit (E-CK-A211) was purchased from Elabscience Biotechnology Co., Ltd. (Wuhan, China). The Cell Cycle and Apoptosis Analysis Kit (C1052), GSH-Tracker NIR (S1087M), Reactive Oxygen Species Assay Kit (S0033M), Beyo3D™ Calcein/PI Cell Viability/Cytotoxicity Assay Kit (C1371M), Beyo3D™ Reactive Oxygen Species Assay Kit with DCFH-DA (S1105M), Apoptosis and Necrosis Detection Kit with YO-PRO-1 and PI (C1075M), Mitochondrial Superoxide Assay Kit with MitoSOX™ Red (S0061M), Enhanced Mitochondrial Membrane Potential Assay Kit with JC-1 (C2003S), LDH Cytotoxicity Assay Kit (C0016), Mito-Tracker Green (C1048), and Hoechst 33342 Staining Solution (C1029) were purchased from Beyotime Biotechnology Co., Ltd. (Shanghai, China). All other chemical and biological reagents were used without modification.

***2. Preparation and characterizations of cesium-doped Prussian blue nanoparticles (Cs-PB)***

Cesium-doped Prussian blue nanoparticles (Cs-PB) are typically synthesized via a citrate-capped dual-precursor co-precipitation approach: 406 mg of FeCl₃·6H₂O (1.5 mmol) and 505 mg of CsCl (3 mmol) are dissolved in 50 mL of 0.25 M citric acid solution to formulate the iron precursor A, while 126.7 mg of K₄Fe(CN)₆·3H₂O (0.3 mmol) is dissolved in 550 mL of the same citric acid solution to formulate the ferrocyanide precursor B; under vigorous stirring, solution A is added dropwise into solution B over a 10 min period, and the mixture is then heated to 60 °C and aged for an additional 20 min, after which the precipitate is collected by centrifugation and washed three times with deionized water to yield Cs-PB nanoparticles with high crystallinity and superior peroxidase-like activity. This method leverages the high distribution coefficient and low hydration energy of Cs⁺ to enable its in-situ incorporation into the tetrahedral sites of the Prussian blue lattice during precipitation, substantially reducing [Fe(CN)₆] vacancies, increasing the coordination number of iron centers (forming FeN₅/FeN₆ sites), and fundamentally reprogramming the material’s catalytic selectivity.

The elemental composition of Cs-PB was determined by inductively coupled plasma optical emission spectrometry (ICP-OES; 7800, Agilent, USA). Powder X-ray diffraction (PXRD) patterns were collected on a multipurpose X-ray diffractometer (SmartLab SE, Rigaku, Japan) with Cu Kα radiation (λ = 1.5406 Å) over a 2θ range of 10–80°. Full-pattern Rietveld refinement was performed using a face-centered cubic Prussian blue structure model to determine lattice parameters, crystallite size, and relative crystallinity. Morphology and elemental distribution of Cs-PB were characterized by transmission electron microscopy (TEM; JEM 2100, JEOL, Japan) coupled with energy-dispersive X-ray spectroscopy (EDS) mapping. Moreover, surface chemical states of Cs-PB were characterized by X-ray photoelectron spectroscopy (XPS; Thermo Scientific™ K-Alpha™, Thermo Fisher Scientific, USA) using a monochromatic Al Kα source, and the valence states were determined via peak deconvolution of the high-resolution core-level spectra employing a mixed Gaussian–Lorentzian fitting function. Cesium release behavior was evaluated by incubating Cs-PB in phosphate-buffered saline (PBS, pH 7.4) or acetate buffer (pH 6.5) at 37°C with gentle shaking. At predetermined time intervals, samples were centrifuged, and the Cs⁺ concentration in the supernatant was measured by ICP-OES to calculate cumulative release percentage.

***3. Synthesis of injectable nanodepots***

Firstly, HA-DA was synthesized according to a previously reported procedure. Briefly, 1 g of HA was dissolved in 100 mL of PBS, and the pH of the solution was adjusted to 5.5 using an aqueous hydrochloric acid solution. The mixture was purged with nitrogen for 30 min, followed by the addition of 338 mg of EDC and 204 mg of NHS. After activating the carboxyl groups for 15 min, 474 mg of DA was added. The reaction was maintained at pH 5.5 and stirred in the dark for 24 hours under a nitrogen atmosphere. The resulting solution was extensively dialyzed to remove unreacted reagents and urea byproducts, and the purified conjugate was finally obtained by lyophilization. To prevent oxidation, the final HA-DA powder was stored at 4 °C under vacuum in a light-proof desiccator.

Subsequently, HA-DA powder was accurately weighed using an analytical balance and dissolved in purified water, followed by thorough stirring to obtain a 4 wt% HA-DA (HD) aqueous solution. Cs-PB at 0.1 wt%, the sonosensitizer Ce6 at 0.1 wt%, and the glutaminase inhibitor Tela at 0.5 wt% were sequentially introduced into the HD solution and stirred for an additional 12 hours in the dark. After thorough mixing, 0.05 M hydrogen peroxide (H₂O₂) was introduced to initiate crosslinking at room temperature, yielding the Tela/Ce6/Cs-PB@HA-DA (TCPD) injectable nanodepots. Meanwhile, Ce6/Cs-PB@HA-DA (CPD) and Cs-PB@HA-DA (PD) injectable nanodepots were prepared following the same protocol. Moreover, horseradish peroxidase (HRP)-catalyzed HD hydrogels were fabricated as controls by introducing HRP at an activity equivalent to that of the Cs-PB group into the HA-DA solution, followed by crosslinking with 0.05 M H₂O₂. The detailed compositions of PD, CPD, and TCPD are detailed in **Tables S1**.

***4. Physicochemical characterizations of injectable nanodepots***

Fourier transform infrared spectroscopy (FTIR; TNZ1-5700, Nicolet, USA) and Raman spectroscopy (DXR2, Thermo Fisher Scientific, USA) were performed to analyze the chemical groups of raw materials and nanodepots, and the scanning range of the test was 500-4000 cm^-1^. The surface morphology and elements were observed using scanning electron microscopy (SEM; JSM-7401 F, JEOL, Japan) with EDS analysis system. The encapsulation efficiency (EE) and wet-weight loading content (LC) of therapeutic payloads within the nanodepots were quantitatively determined. The EE was calculated as the ratio of the actual drug amount in the hydrogel to the initial feeding amount, while the LC was expressed as the mass percentage of encapsulated drug relative to the total wet hydrogel weight. The injectability of injectable composite hydrogels was evaluated by drawing up and injecting assays using 23G and 26G syringes, respectively. The thermal stability of the nanodepots was comprehensively evaluated using thermogravimetric-differential scanning calorimetry (TG-DSC) measurements from a simultaneous thermal analyzer (STA 509 Jupiter Classic, NETZSCH, Germany).

***5. Viscosity and rheological properties***

The rheological behavior of the injectable nanodepots (diameter, 20 mm; thickness, 1 mm) was characterized using a rotational rheometer (HAAKE MARS 60, Thermo Fisher Scientific, USA) equipped with a Peltier temperature-controlled bottom plate set at 37 °C. Dynamic viscosity was evaluated under rotational mode by recording steady-state flow curves during a linearly increasing shear rate ramp from 0.01 to 100 s⁻¹. The resulting viscosity–shear rate profiles were used to assess shear-thinning behavior, and the flow behavior index was derived by fitting the data to the power-law model. Viscoelastic moduli were further determined under oscillatory mode through strain amplitude sweeps conducted at a fixed frequency of 1 Hz over a strain range of 0.1-1000 %. The storage modulus (G′) and loss modulus (G″) were recorded as functions of applied strain, and the linear viscoelastic region (LVER) was identified as the strain range over which both moduli remained independent of strain. The gel-like, solid-dominant character of the nanodepots was confirmed by G′ exceeding G″ within the LVER.

***6. Swelling behavior***

The swelling behavior of nanodepots was evaluated using a gravimetric method. The samples were freeze-dried to constant weight, and the dry weight (Wd) was recorded. Subsequently, the samples were immersed in phosphate-buffered saline (PBS) at pH = 7.4, respectively, and incubated at 37°C in a constant-temperature shaking incubator. At predetermined time points (0, 0.5, 1, 2, 4, 6, 12, 24, 48, and 72 h), the samples were removed, gently blotted with filter paper to remove surface excess liquid, and immediately weighed to obtain the wet weight (Wt). Three parallel samples were set for each time point, and the experiment was repeated three times. The swelling ratio was calculated according to the following formula:

$$Swelling ratio \left( \% \right)=\frac{W_{t-}W_{d}}{W_{d}}\times100\%$$

W_d_, the dry weight mass; W_t_, the measured wet mass at predetermined time points.

***7. Hemolysis ratio assay***

For the hemolytic ratio test, fresh whole blood was extracted from mice ophthalmic vein and diluted with saline at a ratio of 1:1.25. The samples were subsequently co-incubated with the diluted blood at 37°C for 1 hour. The hemolysis status was observed after centrifugation at 3000 rpm for 10 min. The optical density (OD) at 545 nm of the supernatant was measured using a microplate analyzer (NanoDrop, Thermo Fisher, USA). The hemolysis ratio was calculated by the following equation:

$$Hemolysis ratio \left( \% \right)=\frac{{OD}_{s}-{OD}_{n}}{{OD}_{p}-{OD}_{n}}\times100\%$$

ODs, the OD values of the sample; ODp, the OD values of the positive control; and ODn, the OD values of the negative control.

***8. Enzymatic activities evaluation***

*Peroxidase-like and glutathione peroxidase-like activities evaluation:* The intrinsic peroxidase (POD)-like and glutathione peroxidase (GSH-Px)-like catalytic activities of the TCPD nanodepots were systematically evaluated using 3,3′,5,5′-tetramethylbenzidine (TMB) and 5,5′-dithiobis(2-nitrobenzoic acid) (DTNB) as chromogenic substrates, respectively. To dissect the synergistic contribution of each modality, the following cohorts were compared: (G1) Control, consisting of PBS only; (G2) TCPD, containing TCPD nanodepots; (G3) CPD+US+MW, containing CPD nanodepots followed by microwave (MW) and ultrasound (US) irradiation; (G4) TCPD+MW, containing TCPD nanodepots followed by MW irradiation; (G5) TCPD+US, containing TCPD nanodepots followed by US irradiation; and (G6) TCPD+US+MW, containing TCPD nanodepots followed by MW and US irradiation. For POD-like activity, TCPD or CPD nanodepots (equivalent doses of Ce6 and Cs-PB) were mixed with containing 5 μL TMB (10 mg/mL) and 20 μL H₂O₂ (1 mmol/L) in 925 μL deionized waters. For GSH-Px-like activity, glutathione (GSH) and H₂O₂ (1 mmol/L) were provided as co-substrates, and 5 μL DTNB (0.2 mM) was introduced to derivatize residual thiols after the reaction. To further delineate the maximal catalytic capacity of the fully stimulated TCPD nanodepots, a multidimensional parameter optimization was conducted exclusively for the TCPD+US+MW group under the principle of controlling a single variable at a time. Concentration-dependent activities were profiled across a TCPD gradient (0, 3, 5, 10, 20, and 30 mg/mL). Power density-dependent catalytic responses were examined under MW (0, 0.5, 1, 3, 5, and 7 W/cm², 2.45 GHz; WB-3100, BXING MEDICALEQUIPMENT, China) and US (0, 0.2, 0.5, 1, 1.5, and 2 W/cm², 1 MHz; Intelect Mobile Ultrasound DJO 2776, Chattanooga, USA) irradiation. Time-dependent peroxidase-like activity was monitored over 0, 1, 3, 5, 7, and 9 min, while glutathione peroxidase-like activity was assessed at 0, 10, 15, 20, 25, and 30 min. The increase in oxidized TMB absorbance at 652 nm and the decrease in DPBF absorbance at 410 nm were recorded via a UV–visible spectrophotometer (UV-3600 Plus, Shimadzu, Japan).

*Hydroxyl radical (·OH) generation assessment:* Methylene blue (MB) as a chromogenic probe, whose characteristic absorbance at 664 nm decays upon oxidation by hydroxyl radical (·OH). To verify the synergistic ·OH production, the same six groups (G1–G6) were compared under identical grouping logic. Specifically, TCPD or CPD nanodepots (equivalent doses of Ce6 and Cs-PB) were mixed with containing 5 μL MB (1 mg/mL) and 20 μL H₂O₂ (1 mmol/L) in 925 μL deionized waters, and the mixture was exposed to the designated MW and/or US irradiation in the dark. To further characterize the maximal ·OH-generating capacity, a multidimensional optimization was conducted exclusively for the TCPD+US+MW group by controlling a single variable at a time. Concentration-dependent, power density-dependent, and time-dependent ·OH generation activities were examined using the same parameter ranges as described above for the POD-like activity assay. The decrease in MB absorbance at 664 nm was recorded using a UV–visible spectrophotometer.

*Singlet oxygen (^1^O_2_) generation assessment:*1,3-diphenylisobenzofuran (DPBF) was used as a molecular probe to assess singlet oxygen (^1^O_2_) generation. To verify the synergistic ¹O₂ production, the same six groups were compared under identical grouping logic. Specifically, TCPD or CPD nanodepots (equivalent doses of Ce6 and Cs-PB) were mixed with 40 μL DPBF (8 mM) in 910 μL deionized waters, and the mixture was exposed to the designated MWA and/or US irradiation in the dark. To further characterize the maximal ¹O₂-generating capacity, a multidimensional optimization was conducted exclusively for the TCPD+US+MW group by controlling a single variable at a time. Concentration-dependent activity was profiled across a TCPD nanodepot gradient (0, 3, 5, 10, 20, and 30 mg/mL) under fixed physical parameters. Power density-dependent responses were examined at the optimal TCPD concentration by independently varying US irradiation (0, 0.2, 0.5, 1, 1.5, and 2 W/cm², 1 MHz), with the complementary modality held constant. Time-dependent activity was monitored over 0, 1, 3, 5, 7, and 9 min. The decrease in DPBF absorbance at 410 nm was recorded using a UV-visible spectrophotometer.

***9. In vitro microwave thermal conversion performance evaluation***

To systematically assess the intrinsic microwave-to-thermal conversion performance of TCPD nanodepots *in vitro*, thermal performance assays were meticulously stratified into parameter-dependent monitoring, parallel group comparative profiling, and cyclic stability phases. Initially, a comprehensive comparative thermal evaluation was systematically conducted to decipher the specific triggers for thermogenesis across predetermined treatment configurations: (G1) Control, consisting of PBS only; (G2) CPD+US+MW, containing CPD nanodepots followed by US and MW irradiation; (G3) TCPD+MW, containing TCPD nanodepots followed by MWA; (G4) TCPD+US, containing TCPD nanodepots followed by US irradiation; and (G5) TCPD+US+MW, containing TCPD nanodepots followed by US and MW irradiation. Subsequently, for the concentration-dependent and power-dependent thermal analyses, TCPD nanodepots across a well-defined concentration gradient (0, 10, 20, 25, and 30 mg/mL) were exposed to fixed physical parameters, whereas an optimized concentration of TCPD nanodepots was independently subjected to MW irradiation at a spectrum of precisely calibrated power densities (0, 1, 3, 5, and 7 W/cm²). Spatiotemporal temperature evolutions across all evaluated cohorts were dynamically tracked and recorded in real time utilizing an infrared thermal imaging camera (Testo 865, Shanghai, China) to generate holistic thermographic profiles. Furthermore, to rigorously validate the thermal durability and reproducible microwave-thermal endurance of the formulation, the optimized TCPD nanodepots were challenged with four consecutive heating-cooling cycles under targeted MW irradiation at varying power densities of 3, 5, and 7 W/cm², each followed by natural convective cooling, with all real-time thermodynamic trajectories meticulously documented.

***10. In vitro pH- and stimuli-responsive Tela and Ce6 release kinetics***

To investigate the stimuli-responsive release profiles of Tela and Ce6, 50 mg TCPD nanodepots containing 50 μg Ce6 and 250 μg Tela were enclosed in dialysis bags (MWCO 8-14 kDa) and immersed in 50 mL PBS at two distinct pH values, pH 7.4 to simulate normal physiological conditions and pH 6.5 to mimic the mildly acidic tumor microenvironment. Given the limited aqueous solubility of both Ce6 and Tela, the release medium volume was selected to ensure that theoretical maximum drug concentrations upon complete release, 1 μg/mL for Ce6 and 5 μg/mL for Tela, would remain substantially below their respective saturation limits. The entire system was incubated at 37 °C with continuous orbital shaking at 100 rpm. Moreover, to evaluate the triggering efficacy of exogenous physical stimuli, samples under both pH conditions were assigned to four parallel groups: (1) Control, receiving no physical stimulation; (2) MW irradiation alone; (3) US irradiation alone; and (4) combined MW and US irradiation. For the stimulated groups, irradiation was applied immediately before each scheduled sampling time point. At predetermined intervals, 1 mL of the release medium was withdrawn from each vessel and immediately replaced with an equal volume of fresh, pre-warmed PBS at the corresponding pH. The collected samples were subsequently diluted with the mobile phase, and the concentrations of both Tela and Ce6 were simultaneously quantified by reversed-phase high-performance liquid chromatography (HPLC; LC-20A, Shimadzu, Japan) based on their distinct retention times and absorption maxima. The cumulative release percentage was calculated with volume correction using the following equation:

$$Cumulative release (\%)=\frac{V_{0}C_{n}+V_{s}\sum_{i=1}^{t-1} C_{i}}{M_{loaded}}\times100\%$$

V_0_, the total volume of the release medium; C_n_, the drug concentration in the release medium at time n; V_s_, the volume of the withdrawn sample (1 mL); C_i_, the concentration of the drug measured at time i; and M_loaded_, the initial mass of the drug perfectly encapsulated within TCPD nanodepots.

***11. Cellular experiments***

To systematically evaluate the multidimensional therapeutic efficacy, a comprehensive panel of murine cells, incorporating hepatic carcinoma cells H22 and Hepa1-6 as tumor models and bone marrow-derived dendritic cells (BMDCs) alongside RAW264.7 macrophages as immune models, was utilized. Prior to mechanistic investigations, an initial cytotoxicity screening was performed using the Cell Counting Kit-8 (CCK-8) assay across a range of formulations and stimuli configurations. As shown in **Fig. S15**, the HD, PD, and CPD nanodepots alone, as well as the US and MW physical stimuli alone, exhibited negligible intrinsic cytotoxicity. Moreover, free Ce6 combined with US irradiation, free Cs-PB or Tela combined with US and MW irradiation, as well as PD nanodepots combined with US and MW irradiation, demonstrated significantly lower efficacy compared to the integrated TCPD nanodepots. Based on these preliminary findings, six experimental groups were established to evaluate the therapeutic mechanisms as follows: (G1) Control, receiving an equivalent volume of PBS; (G2) TCPD, incubated with TCPD nanodepots without exogenous physical triggers; (G3) CPD+US+MW, incubated with CPD nanodepots followed by US and MW irradiation; (G4) TCPD+MW, incubated with TCPD nanodepots followed by MW irradiation; (G5) TCPD+US, incubated with TCPD nanodepots followed by US irradiation; and (G6) TCPD+US+MW, incubated with TCPD nanodepots followed by US and MW irradiation. All TCPD and CPD nanodepot-treated groups were administered at a standardized equivalent therapeutic dose (Cs-PB at 20 μg/mL, Ce6 at 20 μg/mL, and/or Tela at 100 μg/mL). MW irradiation was uniformly performed using a custom-designed coaxial monopole antenna (15 mm effective radiating length, 1.0 mm outer diameter, polytetrafluoroethylene-insulated) connected to a solid-state MW generator (SPW-1A, Xuzhou Shengpu Medical Equipment Technology Co., Ltd., Xuzhou, China). US irradiation was delivered using a planar transducer integrated into a portable therapeutic ultrasound system (Chattanooga DJO 2776, Enovis Corporation, Delaware, USA) with acoustic gel (Aquasonic 100, Parker Laboratories, New Jersey, USA). For groups involving exogenous physical stimuli, cells were exposed to MW (2.45 GHz, 5 W, 3 min) and/or US irradiation (1.0 MHz, 1.5 W/cm² ISPTA, 50% duty cycle, 3 min).

***11.1. Glutamine metabolism and TCA cycle intermediates biochemical quantification***

H22 cells were seeded into 6-well plates at 2 × 10⁵ cells per well and incubated with designated treatments for 6 h. Subsequently, cells were rapidly washed twice with ice-cold PBS, immediately quenched with pre-chilled 80% (v/v) methanol, and scraped into cold microcentrifuge tubes. After vigorous vertexing for 1 min, cell suspensions were centrifuged at 15000 × g for 15 min at 4°C, and the metabolite-rich supernatants were collected. For tumor tissue samples, freshly harvested tissues were immediately snap-frozen in liquid nitrogen, weighed, and homogenized in extraction buffer at a ratio of 10 mg tissue per 100 μL buffers using a mechanical homogenizer on ice. Lysates were centrifuged at 12000 × g for 10 min at 4°C, and supernatants were collected for analysis. Intracellular levels of glutamine, glutamate, glutaminase activity, and TCA cycle intermediates (citrate, isocitrate, α-ketoglutarate, succinate, fumarate, malate, and oxaloacetate), as well as GLS1 enzymatic activity, were quantified using commercial colorimetric assay kits according to the manufacturer’s instructions (**Table. S2**). Standards were prepared according to kit protocols to generate calibration curves. Absorbance was measured at the specified wavelength using a microplate reader. Metabolite concentrations and enzyme activity were calculated from standard curves, normalized to total protein content determined by an Enhanced BCA Protein Assay Kit (P0009, Beyotime, Shanghai, China), and expressed as relative fold changes compared to the control group.

***11.2. Cellular metabolic flux real-time assessment***

To interrogate the dynamic shifts in mitochondrial respiration and glycolytic function, the oxygen consumption rate (OCR) and extracellular acidification rate (ECAR) of H22 cells were assessed using a Seahorse XF Pro Analyzer (Agilent, USA). Cells were seeded into XF96 microplates at a density of 1 × 10⁴ cells per well and cultured overnight, followed by 6 h of exposure to the designated treatments. For mitochondrial stress testing, prior to measurement, the medium was replaced with unbuffered Seahorse XF Base Medium (supplemented with 2 mM glutamine, 1 mM pyruvate, and 10 mM glucose, pH 7.4) and equilibrated in a non-CO₂ incubator at 37 °C for 1 h. OCR was then recorded under basal conditions and sequentially following the automated injection of: (i) ATP synthase inhibitor oligomycin (1 μM), (ii) mitochondrial uncoupler carbonyl cyanide-4-(trifluoromethoxy)phenylhydrazone (FCCP; 1.5 μM), and (iii) Complex I & III inhibitors rotenone (0.5 μM) plus antimycin A (0.5 μM). For glycolysis stress testing, a separate set of wells was similarly prepared and equilibrated. ECAR was monitored under basal conditions and subsequently after the automated injection of: (i) glucose (10 mM), (ii) oligomycin (1 μM), and (iii) hexokinase inhibitor 2-deoxy-D-glucose (2-DG; 50 mM). Bioenergetic parameters including basal respiration, ATP-linked respiration, maximal respiratory capacity, spare respiratory capacity, basal glycolysis, glycolytic capacity, and glycolytic reserve, were calculated using Seahorse Wave Pro software (Agilent, USA). All values were normalized to the cellular protein content of each well, determined by an Enhanced BCA Protein Assay Kit performed immediately after the flux assay.

***11.3. Oxidative stress assessment***

H22 cells (1 × 10⁵/mL) were co-incubated with different interventions for 6 hours at 37°C, followed by staining with distinct fluorescent probes: total reactive oxygen species (ROS) was accessed using the 2′,7′-dichlorodihydrofluorescein diacetate (DCFH-DA; 10 μM) with Hoechst 33342 (1 μg/mL); ^1^O_2_ was assessed using Singlet Oxygen Sensor Green (SOSG; 5 μM) with Hoechst 33342 (1 μg/mL); and GSH was assessed using GSH-Tracker NIR probe (20 μM) with Hoechst 33342 (1 μg/mL); In all cases, H22 cells were incubated with the probes for 30 min as appropriate, washed twice with PBS, and subsequently imaged by a fluorescence microscopy (IX71, Olympus Corporation, Japan) and analyzed by Image J (National Institutes of Health, Bethesda, USA).

***11.4. Three-dimensional tumor spheroid penetration and spatially complementary cytotoxicity assay***

*Three-dimensional tumor spheroids established:* To evaluate the spatially complementary cytotoxicity, three-dimensional (3D) tumor spheroids were established utilizing Hepa1-6 cells, which were specifically selected for their superior propensity to spontaneously assemble into highly compact spheroids relative to H22 cells. Briefly, Hepa1-6 cells were seeded into ultra-low attachment 96-well plates at a density of 1 × 10³ cells per well. The microplates were cultured for 7 days until tightly aggregated, uniform spheroids approximately 400-500 μm in diameter were successfully formed.

*Spatial complementarity ROS generation:* The treated spheroids were incubated with 20 μM DCFH-DA in serum-free medium at 37 °C for 30 min. After washing thoroughly with PBS to remove background fluorescence, the spheroids were imaged via confocal laser scanning microscopy (CLSM; STELLARIS, Leica, Germany). Z-stack optical sectioning was performed at 20 μm intervals from the spheroid periphery towards the necrotic core. The fluorescence intensity of DCFH-DA was quantified across discrete Z-planes as a function of depth to construct comprehensive spatial ROS distribution profiles.

*Spatial complementarity cytotoxicity evaluation:* Cell viability within the spheroids was assessed using a Calcein-AM (2 μM) and propidium iodide (PI; 4 μM) co-staining assay. Following a 10-min incubation at 37 °C in dark and subsequent PBS washing, the spheroids were similarly subjected to confocal Z-stack scanning. Viable cells were visualized exhibiting green fluorescence, whereas dead cells displayed red fluorescence. Three-dimensional architectural reconstructions of the live and dead optical slices were generated to accurately quantify and map the spatial patterns of cell death across varying depth zones.

***11.5. Anti-proliferation and migration assessment***

To assess the proliferative capacity and migratory potential of tumor cells surviving the respective treatments, Hepa1-6 tumor spheroids were first subjected to the designated interventions (G1–G6) as described above. After treatment, spheroids were collected, washed twice with PBS, and enzymatically dissociated into single-cell suspensions by incubation with 0.25% trypsin-EDTA at 37 °C for 3–5 min with gentle pipetting. Complete DMEM medium containing 10% fetal bovine serum was added to terminate digestion. The single-cell suspensions were passed through a 40 μm cell strainer to remove residual aggregates, centrifuged at 300 × g for 5 min, and resuspended in fresh complete medium.

*Cell cycle assay:* Equal numbers of viable cells from each treatment group (1 × 10^5^/mL) were washed with ice-bath pre-cooled PBS. Subsequently, 1 ml of ice-bath pre-cooled 70% ethanol was added and fixed at 4ºC for 30 min. Centrifuge again, wash with ice-bath pre-cooled PBS and resuspend. The appropriate amount of PI staining solution was prepared according to the manufacturer’s instructions. 0.5 ml of PI staining solution was added to each tube of cell samples, and the cell precipitates were resuspended slowly and well, and the warm bath was protected from light at 37ºC for 30 min. The red fluorescence at the excitation wavelength of 488 nm was detected using flow cytometry (NovoCyte 3130, ACEA Biosciences, USA), simultaneously with the detection of light scattering.

*CCK-8 assay:* Equal numbers of viable cells from each treatment group (3 × 10^3^/100 μL) were seeded into 96‑well plates and cultured in complete medium at 37 °C under 5% CO₂. At the designated endpoint (12, 24, 36, 48, and 72 h), 10 μL of CCK‑8 reagent (Dojindo, Japan) was added to each well, and the plates were incubated for an additional 3 h at 37 °C. The absorbance value at 450 nm was detected with a microplate analyzer (NanoDrop, Thermo Fisher, USA).

*Transwell assay*: Equal numbers of viable cells from each treatment group (5 × 10^4^/mL) were seeded into the upper compartment of a Transwell chamber. Subsequently, the corresponding treatments were added to the upper chamber for the different groups. After incubation for 36 hours, the migrated cells on the lower surface of the filter were stained with 0.5% crystal violet solution, fixed in 4% paraformaldehyde, and examined by light microscopy. The number of migrating cells in each group was analyzed using ImageJ software.

***11.6. Transcriptome sequencing and high-throughput analysis***

The H22 cells from the G1 and G6 groups (three samples per group) was flash-frozen in liquid nitrogen and subsequently pulverized into a fine powder. Total RNA was extracted from the tissue using TRIzol reagent, followed by purification for library preparation and sequencing on an Illumina HiSeq™ platform. Raw single-strand sequencing data were aligned using the Salmon package and mapped to the GRCm38 reference genome. Transcripts permillion values were calculated for each gene. Differential expression analysis was carried out with the DESeq2 R package, considering genes as significantly differentially expressed when the fold change exceeded 2 and the adjusted p value (q value) was below 0.05. Subsequently, Reactome enrichment analysis, Gene Set Enrichment Analysis (GSEA), and protein-protein interaction (PPI) analysis for differentially expressed genes (DEGs) was performed using the clusterProfiler R package.

***11.7. PANoptosis assessment***

*PANoptosis executioner examination:* The concomitant activation networks of terminal executioner proteins mediating the three interconnected cell death modalities were systematically profiled via Western blotting. These parallel effectors encompassed: (i) pyroptosis-related mediators, including caspase-1, cleaved caspase-1, Gasdermin D (GSDMD), and its active N-terminal fragment (GSDMD-N); (ii) necroptosis-associated proteins, featuring receptor-interacting protein kinase 3 (RIPK3), phosphorylated RIPK3 (p-RIPK3), mixed lineage kinase domain-like pseudokinase (MLKL), and phosphorylated MLKL (p-MLKL); as well as (iii) canonical apoptosis indicators, such as caspase 3 and cleaved caspase 3 (c-caspase 3).

*PANoptosome sensor examination:* To validate the stress-induced formation of the upstream PANoptosome complex, key pattern recognition receptors governing this machinery were quantified via Western blotting. Specifically, the expression levels of critical sensors including NOD-like receptor family pyrin domain containing 3 (NLRP3), NLR family CARD domain containing 5 (NLRC5), absent in melanoma 2 (AIM2), and Z-DNA binding protein 1 (ZBP1) were systematically analyzed.

*Immunofluorescence Staining:* H22 cells (1 × 10⁵ cells per well) were seeded onto confocal dishes and subjected to designated treatments for 24 h. Cells were fixed with 4% paraformaldehyde for 20 min at room temperature, permeabilized with 0.3% Triton X-100 for 15 min, and blocked with 3% BSA for 1 h at room temperature. Cells were sequentially incubated with anti-p-MLKL, anti-c-caspase 3, and anti-GSDMD-N primary antibodies overnight at 4°C. After washing three times with PBS, cells were incubated with corresponding fluorophore-conjugated secondary antibodies for 1 hour at room temperature in the dark. The cell nuclei were stained with 4’,6-diamidino-2-phenylindole (DAPI). A CLSM was used for data acquisition, and ImageJ was used for data analysis.

*Western blot assay:* The total proteins of H22 cells were extracted with RIPA lysis buffer (R0020, Solarbio, Beijing, China) supplemented with phenylmethylsulfonyl fluoride (PMSF, BL507A, Biosharp), followed by homogenization and sonication, and then centrifugation for 10 min at 4 °C. Subsequently, the concentrations of proteins were quantified by a BCA assay. Proteins were then separated by dodecyl sulfate, sodium salt-polyacrylamide gel electrophoresis (SDS-PAGE) and transferred to polyvinylidene fluoride (PVDF) membranes. The membranes were blocked with 5% BSA at room temperature overnight, probed with PANoptosis proteins executioners and PANoptosome sensors proteins primary antibodies at room temperature for 2 hours, followed by anti-rabbit or anti-mouse secondary antibodies. The protein bands were visualized using ECL detection reagent (GE Healthcare, USA). An anti β Actin polyclonal antibody was used as the endogenous control. For ASC knockdown experiments, H22 cells were seeded in 6-well plates at 2 × 10⁵ cells per well and transfected with ASC-specific siRNA or scrambled control siRNA using Lipofectamine™ RNAiMAX transfection reagent (13778100, Thermo Fisher Scientific, Massachusetts, USA) according to the manufacturer’s protocol. After 48 hours of transfection, cells were subjected to different treatments for an additional 24 hours, followed by Western blot assay to detect PANoptosis executioner proteins. The details of primary and secondary antibodies are listed in **Table S3** and **Table S4**, respectively.

*Co-Immunoprecipitation (Co-IP) assay:* H22 cells were seeded in 10 cm dishes at 5 × 10⁶ cells per dish and treated with designated interventions for 24 h. Cells were washed twice with ice-cold PBS and lysed in Pierce™ IP lysis buffer (87787, Thermo Fisher Scientific, Massachusetts, USA) supplemented with protease inhibitor cocktail and phosphatase inhibitor cocktail on ice for 30 min. Lysates were centrifuged at 12000 × g for 15 min at 4°C, and protein concentrations in supernatants were quantified using an Enhanced BCA Protein Assay Kit. Equal amounts of protein were incubated with anti-ASC polyclonal antibody or normal IgG overnight at 4°C with gentle rotation. Protein A/G magnetic beads (88802, Thermo Fisher Scientific, Massachusetts, USA) were added and incubated for an additional 4 h at 4°C. Beads were washed five times with ice-cold IP lysis buffer, and bound proteins were eluted by boiling in 1× SDS loading buffer for 10 min. Immunoprecipitated proteins were analyzed by Western blotting using anti-ZBP1 polyclonal antibody, anti-AIM2 monoclonal antibody, anti-NLRP3 polyclonal antibody, anti-Pyrin polyclonal antibody, anti-caspase-1 polyclonal antibody, anti-caspase-8 polyclonal antibody, anti-RIPK1 polyclonal antibody, and anti-RIPK3 monoclonal antibody to detect PANoptosome component interactions. Input lysates were included as loading controls.

*Cell death modality assay:* To comprehensively evaluate the varied cell death modalities, cells were first seeded on 6-well plates at a density of 1 × 10^5^ cells/mL and cultured overnight, followed by incubation with designated interventions for 24 hours. To quantify apoptosis, the treated H22 cells were harvested, washed, and dual-stained with Annexin V-FITC and PI for 20 min in the dark, followed by analysis using a flow cytometer. For the phenotypic visualization of necroptosis, cells were washed with PBS and co-stained with YO-PRO-1 and PI for 20 min. Fluorescence images were subsequently captured via a fluorescence microscope and quantified utilizing ImageJ software. Furthermore, to specifically examine pyroptotic execution, the morphological hallmarks of treated Hepa1-6 cells (such as cell swelling and membrane ballooning) were evaluated and recorded under bright-field microscopy.

*Lactic dehydrogenase, IL-1β, and IL-18 evaluation:* H22 cells were seeded on 96-well plates at a density of 5×10³cells/well. After the different interventions as mentioned above, the release of lactic dehydrogenase (LDH) was measured according to the instructions of the LDH cytotoxicity test kit. After the same treatment as mentioned above. the supernatant was collected to detect the release of IL-1β and lL-18 through ELISA kits, respectively.

***11.8. Mitochondrial damage and genotoxicity evaluation***

*Mitochondrial ROS and membrane potential detection:* H22 cells (1 × 10^5^/mL) were co-incubated with different interventions for 6 hours at 37°C, followed by staining with distinct fluorescent probes: mitochondrial ROS was accessed using the MitoSOX™ Red (2 μM) and Mito-Tracker Green (200 nM) probes staining for 60 min; and mitochondrial membrane potential was determined via JC-1 probe staining for 30 min. After washing to remove excess probes, the H22 cells were imaged by fluorescence microscopy.

*Mitochondrial morphology examination:* For mitochondrial morphology examination, H22 cells were incubated with different interventions for 24 hours, immediately fixed in 2.5% glutaraldehyde, dehydrated through a graded series of ethanol and propylene oxide, and subsequently embedded in epoxy resin. Ultrathin sections (70 nm thickness) were prepared using a ultramicrotome (Leica UC7, Leica, Germany), stained with uranyl acetate and lead citrate, and digitally imaged with a TEM.

*Mitochondrial outer membrane permeabilization (MOMP) evaluation:* To evaluate mitochondrial outer membrane permeabilization (MOMP), the expression of key regulatory proteins and the translocation of cytochrome c (Cyt c) were assessed by Western blot analysis. Total protein was extracted using RIPA lysis buffer supplemented with a protease inhibitor cocktail, and protein concentrations were determined using a BCA protein assay kit. For detection of long-form optic atrophy 1 (L-OPA1), protein samples were incubated at 37°C for 30 min to preserve protein integrity and avoid boiling-induced aggregation. Other whole-cell lysates and cytosolic fractions were denatured at 95°C for 5 min for analysis of B-cell lymphoma 2 (Bcl-2) and BCL-2-associated X protein (BAX). Cytosolic fractions were isolated using a Mitochondrial/Cytosolic Fractionation Kit (ab65320, Abcam, Cambridge, UK) according to the manufacturer's protocol. Equal amounts of protein were separated by SDS-PAGE using gradient gels (8% for L-OPA1, 12% for Bcl-2/BAX, and 15% for cytosolic Cyt c) and transferred onto PVDF membranes. After blocking in 5% non-fat milk for 1 h at room temperature, membranes were incubated overnight at 4°C with the following primary antibodies: anti-Bcl-2 monoclonal antibody, anti-BAX polyclonal antibody, anti-OPA1 polyclonal antibody, and anti-Cyt c polyclonal antibody. The release of Cyt c into the cytosol, together with the expression levels of Bcl-2, BAX, and L-OPA1, was analyzed to assess mitochondrial apoptotic signaling and membrane integrity. β-Actin and β-Tubulin served as loading controls for whole-cell lysates and cytosolic fractions, respectively.

*Cytosolic mitochondrial DNA (mtDNA) quantification:* H22 cells (2 × 10⁶) were seeded in 10 cm dishes and treated with designated interventions for 24 h. Cells were harvested by trypsinization, washed twice with ice-cold PBS, and pelleted at 500 × g for 5 min at 4°C. Cell pellets were resuspended in cytosolic extraction buffer (10 mM HEPES pH 7.4, 10 mM KCl, 0.1 mM EDTA, 0.1 mM EGTA, 1 mM DTT, and protease inhibitor cocktail), incubated on ice for 15 min, then lysed with 0.025% digitonin for 10 min on ice. Lysates were centrifuged at 1000 × g for 10 min at 4°C to pellet intact mitochondria and nuclei. The supernatant was transferred to a fresh tube and further centrifuged at 10000 × g for 10 min at 4°C to remove residual mitochondrial contamination. DNA was extracted from the purified cytosolic fraction using DNA Extract All Reagents (4403319, Thermo Fisher Scientific, Massachusetts, USA). Cytosolic mtDNA levels were quantified by qRT-PCR using primers specific for mitochondrial genes (*D-loop*, *Cox1*, and *Nd1*) and normalized to nuclear DNA (18S rRNA). qRT-PCR was performed using PowerTrack™SYBR Green Master Mix (A46110, Thermo Fisher Scientific, Massachusetts, USA) with cycling conditions of 95°C for 10 min, followed by 40 cycles of 95°C for 15 s and 60°C for 1 min. Relative cytosolic mtDNA abundance was calculated using the 2-ΔΔCt method.

*Genotoxicity examination:* H22 cells (1 × 10^5^/mL) were seeded in confocal well and treated with different interventions for 24 h, respectively. Then, the cells were washed with PBS, fixed in 4% paraformaldehyde solution, blocked with 1% BSA, permeabilized with 0.1% Triton X-100 and then incubated with anti-8-Oxo-dG primary antibody and anti-poly(ADP-ribose) (PAR) primary antibody at 4°C for 2 h. After washing three times with PBS, cells were incubated with secondary antibody for 30 min. Finally, nuclei were stained with DAPI and then the stained sections were imaged by fluorescence microscopy.

***11.9. Endoplasmic reticulum stress evaluation***

H22 cells were seeded in 6‑well plates at a density of 1 × 10⁵ cells/mL and cultured overnight. After incubation with the designated interventions for 24 h, cells were harvested and subjected to Western blot analysis following the same protocol as described previously. To assess the PERK-eIF2α-CHOP signaling axis, which serves as a central mediator of endoplasmic reticulum (ER) stress‑induced apoptosis, the expression levels of its key components were detected using the following primary antibodies: anti‑phospho‑PERK (Thr982) polyclonal antibody, anti‑phospho‑eIF2α (Ser51) monoclonal antibody, anti‑ATF4 polyclonal antibody, and anti‑CHOP polyclonal antibody. An anti‑β‑Actin polyclonal antibody was used as the endogenous control.

***11.10. Intracellular NAD^+^/NADH levels measurement***

Intracellular NAD⁺ and NADH levels were measured using an Enhanced NAD^+^/NADH Assay Kit with WST-8 (S0176S, Beyotime, Shanghai, China) following the manufacturer’s protocol. H22 cells were seeded on 6-well plates at 1 × 10⁵ cells/mL and cultured overnight, followed by incubation with different interventions for 24 hours. Cells were washed twice with ice-cold PBS, lysed in 200 μL NAD⁺/NADH extraction solution for 10 min on ice, then centrifuged at 12000 × g for 10 min at 4°C. The supernatant was collected for analysis. For total NAD⁺ and NADH measurement, 20 μL of sample supernatant was added to each well of a 96-well microplate, along with 90 μL of alcohol dehydrogenase (ADH) working solution (prepared by 1:20 dilution in reaction buffer). The plate was incubated at 37°C for 10 min, followed by addition of 10 μL chromogen solution and incubation at 37°C in the dark for 30 min. For selective NADH measurement, 50 μL of sample supernatant was heated at 60°C for 30 min to decompose NAD⁺, cooled to room temperature, and processed identically. Absorbance was measured at 450 nm using a microplate reader. NAD⁺ and NADH contents were calculated from a standard curve, with NAD⁺ content determined by subtracting NADH from total NAD⁺/NADH. All values were normalized to total protein content determined by an Enhanced BCA Protein Assay Kit, and expressed as relative fold changes compared to the control group.

***11.11. cGAS-STING pathway activation assessment***

H22 cells were first seeded on 6-well plates at a density of 1 × 10^5^ cells/mL and cultured overnight, followed by incubation with designated interventions for 24 hours. Subsequently, using the same experimental method as previously described for Western blot assay, the expression levels of STING pathway-related proteins were detected using anti-STING polyclonal antibody, anti-p-STING (Ser366) polyclonal antibody, anti-IRF3 polyclonal antibody, anti-p-IRF3 (Ser396) monoclonal antibody. An anti‑β‑actin polyclonal antibody was used as the endogenous control.

***11.12. ICD assessment***

H22 cells surface exposure of CRT and release of HMGB1 and ATP were examined *in vitro* to investigate ICD induced by different interventions. Specifically, H22 cells (1 × 10^5^/mL) were seeded onto confocal well, treated with different interventions. For CRT surface exposure detection, cells were fixed with 4% paraformaldehyde for 20 min at room temperature without permeabilization to preserve membrane integrity, blocked with 1% BSA for 30 min at 25°C, sequentially incubated with anti-CRT primary antibody and Cy3-labeled secondary antibody. To assess HMGB1 nuclear translocation, H22 cells were fixed, permeabilized with 0.1% Triton X-100, blocked, and incubated with anti-HMGB1 primary antibody followed by Cy3-labeled secondary antibody. Nuclei were counterstained with 4’,6-diamidino-2-phenylindole (DAPI), and fluorescence images were acquired and analyzed using ImageJ software. Culture supernatants were collected to quantify released HMGB1 and ATP using a Mouse HMGB1 ELISA Kit and an Enhanced ATP Assay Kit (S0027, Beyotime, Shanghai, China) according to the manufacturer's instructions, respectively.

***11.13. Mouse BMDCs maturation examination***

Mouse BMDCs were isolated from 4-week-old female BALB/c. BMDCs were then cultured with RPMI‑1640 medium containing granulocyte-macrophage colony-stimulating factor (GM-CSF, 25 ng/mL; P6006, Beyotime, Shanghai, China) and IL-4 (10 ng/mL; P5916, Beyotime, Shanghai, China) for 6 days to obtain immature BMDCs. H22 cells were treated with different interventions for 24 h, after which the treated H22 cells were co-cultured with immature BMDCs in a transwell system with tumor cells in the upper chamber and BMDCs in the lower chamber to assess BMDC maturation based on an established method [1]. The culture mediums of the co-culture system were collected and the concentrations of cytokines such as IL-6, IL-12p70, TNF-α, and IFN-γ were measured by ELISA kits according to the manufacturer’s instructions, respectively. Meanwhile, BMDCs were collected and incubated with anti-mouse CD11c-APC, anti-mouse CD80-PE, and anti-mouse CD86-FITC at 4°C in dark for 30 min. Finally, the percentage of mature BMDCs (CD11c^+^CD80^+^CD86^+^) was quantified by flow cytometry. In addition, the expression and localization of MHC-II and CD86 proteins in BMDCs from different treatments were detected by double immunofluorescence staining.

***11.14. Macrophage phenotypic reprogramming evaluation***

RAW264.7 macrophages (5 × 10⁴ cells/mL) were pre-polarized toward the M2 phenotype by stimulation with IL-4 (100 ng/mL) and IL-13 (100 ng/mL) for 24 hours according to an established protocol [2]. Subsequently, H22 cells were treated with different interventions for 24 h, after which the treated H22 cells were transferred to the upper chamber of a transwell system and co-cultured with M2 RAW264.7 macrophages in the lower chamber for an additional 24 hours. The culture mediums of the co-culture system were collected and the concentrations of cytokines such as IL-1β, IL-10, TNF-α, and TGF-β were measured by ELISA kits according to the manufacturer’s instructions, respectively. Meanwhile, RAW264.7 cells were collected and incubated with anti-mouse F4/80-APC and anti-mouse CD86-FITC or anti-mouse CD206-PE at 4°C in dark for 30 min. Finally, the percentage of M1 RAW264.7 cells (F4/80^+^CD86^+^) and M2 RAW264.7 cells (F4/80^+^CD206^+^) was quantified by flow cytometry. In addition, the expression and localization of iNOS proteins in RAW264.7 cells with different interventions were detected by immunofluorescence staining.

***12. Animal experiments***

Female Balb/C mice (4 weeks old) were purchased from the laboratory animal center of SPF biotechnology co., Ltd (Beijing, China). All implantation procedures were approved by the Experimental Animal Care & Welfare Committee of Southeast University and complied with the National Institutes of Health Guide for the Care and Use of Laboratory Animals.

***12.1. Microwave thermosensitization and large tumor suppression performance***

To evaluate the therapeutic potential of the engineered nanodepots against large liver cancer, a mouse subcutaneous tumor model was established. Briefly, BALB/c mice were subcutaneously engrafted with H22 cells (5 × 10⁶ cells in 100 μL PBS) on the right anterior dorsum. When tumors reached approximately 500 mm³, a volume representing large solid tumors in mouse models via allometric scaling and widely utilized in preclinical oncology [3]. The mice were randomly assigned to six treatment groups (n = 5 per group) for the following intratumoral interventions: (G1) Control, receiving an equivalent volume of saline administration; (G2) TCPD, receiving TCPD nanodepots alone; (G3) CPD+US+MW, receiving CPD nanodepots followed by MWA and US irradiation; (G4) TCPD+MW, receiving TCPD nanodepots followed by MWA; (G5) TCPD+US, receiving TCPD nanodepots followed by US irradiation; and (G6) TCPD+US+MW, receiving TCPD nanodepots followed by MWA and US irradiation.

The day of the initial intervention was designated as Day 0. For all relevant cohorts, saline or nanodepots were administered intratumorally on Day 0 at a standardized single volume of 50 μL, ensuring uniform administration of the components across groups. Specifically, MWA was uniformly performed using a custom-designed coaxial monopole antenna (2.45 GHz, 15 mm effective radiating length, 1.0 mm outer diameter, polytetrafluoroethylene-insulated) connected to a solid-state MW generator (SPW-1A, Xuzhou Shengpu Medical Equipment Technology Co., Ltd., Xuzhou, China) and inserted perpendicularly into the tumor center (approximately 50% of mean tumor depth) under stereotactic guidance. The generator was operated at 5 W forward power in continuous wave mode for 5 min. This intratumoral insertion strategy enabled direct energy deposition within the tumor core while achieving superior impedance matching (reflected power <3%, standing wave ratio < 1.15:1). The antenna remained stationary throughout treatment to maintain consistent thermal field geometry. Concurrently, localized US irradiation was delivered using a planar transducer (1.0 MHz, 10 mm aperture diameter) integrated into a portable therapeutic ultrasound system (Chattanooga DJO 2776, Enovis Corporation, Delaware, USA) and coupled to the tumor surface with acoustic gel (Aquasonic 100, Parker Laboratories, New Jersey, USA). The US protocol utilized 1.5 W/cm² spatial-peak temporal-average intensity (I SPTA) with a 50% duty cycle (5 s ON/5 s OFF) for 5 minutes total duration (150 seconds active irradiation time). Subsequently, MWA and US irradiation were applied on Days 1, 3, and 5 according to the group assignments. Mouse body weights and tumor volumes were recorded every other day, with tumor volume (mm³) calculated using the formula: 0.5 × length × width². On day 14, tumor tissues of mice from each group were collected. The tumor weights, hematoxylin and eosin (H&E) staining, Ki67 immunohistochemistry and TUNEL staining of tumor tissues were used to evaluate the antitumor performance. Moreover, N-GSDMD, p-MLKL, and c-Caspase 3 immunofluorescence were respectively used to evaluate the PANoptosis induction properties. The major organs (heart, liver, spleen, lung, kidney) were extracted for H&E staining to evaluate biosafety *in vivo*.

***12.2. In vivo degradation assessment***

To evaluate the *in vivo* biodegradation behavior and local biocompatibility of TCPD nanodepots, healthy BALB/c mice were subcutaneously injected with TCPD nanodepots or free Ce6 as control. Fluorescence imaging was performed at predetermined time points (days 0, 3, 7, 14, 21, and 28) using an IVIS Spectrum *in vivo* imaging system (IVIS Spectrum, PerkinElmer, USA) to monitor local retention and degradation kinetics. For histological assessment, mice were euthanized on days 7, 14, 21, 28, and 35 post-injection, and tissue samples from the injection site were collected, fixed in 4% paraformaldehyde, embedded in paraffin, sectioned at 5 μm thickness, and stained with H&E to examine nanodepots degradation, cellular infiltration, and tissue compatibility. Blood samples were collected via retro-orbital bleeding on days 1, 3, 7, 14, 21, and 28, and serum was separated by centrifugation at 3000 rpm for 10 min at 4°C. Serum levels of IL-6 and TNF-α were quantified using ELISA kits according to the manufacturer’s instructions to assess inflammatory responses.

***12.3. Metabolic clearance and systemic toxicity evaluation***

For elemental distribution and clearance studies, mice were euthanized on days 7, 14, 21, and 28 post-injections. Tissue samples from the injection site, major organs (heart, liver, spleen, lungs, kidneys), urine, and feces were collected and digested with concentrated nitric acid at 120°C overnight. The digested samples were diluted with deionized water, and cesium and iron contents were quantified by ICP-OES. Urinary and fecal excretion rates were calculated based on cumulative elemental content recovered from urine and feces collections at each time point. Complete blood counts were analyzed using an automated hematology analyzer (BS-600M, Mindray, China) to evaluate hematological toxicity. Hepatic and renal function were assessed by measuring serum levels of albumin (ALB), alanine aminotransferase (ALT), aspartate aminotransferase (AST), blood urea nitrogen (BUN), and creatinine (CREA) using an automatic biochemical analyzer (AU5800, Beckman Coulter, USA).

***12.4. Immune activation and immunomodulation performance***

A mice subcutaneous tumor model was re-established, which were then treated with the same regimen above described for large liver cancer. On day 7, tumor tissues and draining lymph nodes of mice from each group were collected to assess the antitumor immunomodulation effects. After dissociating the tumor tissues and draining lymph nodes into a single-cell suspensions, DCs (anti-CD11c-APC, anti-CD80-PE, and anti-CD86-FITC) in draining lymph nodes, CD4^+^ T lymphocytes (anti-CD3-PE and anti-CD4-FITC), CD8^+^ T lymphocytes (anti-CD3-PE and anti-CD8α-APC), TAMs (anti-CD11b-APC, anti-CD86-FITC, and anti-CD206-PE), Tregs (anti-CD4-FITC and anti-FOXP3-APC), and PMN and M MDSCs (anti-CD11b-FITC, anti-Ly6G-PE, and anti-Ly6C-APC) in tumor tissues were detected by flow cytometry and analyzed FlowJo software. Moreover, the IFN-γ, TNF-α, IL-10, and TGF-β in tumor tissues were examined by ELISA kits. The details of flow cytometry antibodies and ELISA kits are listed in **Table S5** and **Table S6**, respectively.

***12.5.*** ***Distant metastasis suppression and immune memory establishment***

To further explore the potential of TCPD nanodepots combined with US and MW irradiation to suppress distant metastasis and elicit long-term immune memory via synergy with anti-PD-1 therapy, mouse bilateral tumor and lung metastasis models were established. The mice were randomly assigned to four treatment groups (n = 6 per group) to receive the following interventions: (G1) Control, receiving saline administration; (G2) anti-PD-1, receiving systemic administration of anti-PD-1 via tail vein injection; (G3) TCPD+US+MW, receiving intratumoral TCPD nanodepots followed by MWA and US irradiation; and (G4) TCPD+US+MW+anti-PD-1, receiving intratumoral TCPD nanodepots followed by MWA and US irradiation plus systemic anti-PD-1 administration. For all relevant cohorts, MWA and US irradiation were applied on Days 1, 3, and 5; anti-PD-1 therapy was intravenously administered at a dose of 1 mg/kg on Days 2, 4, and 6. The nanodepot formulation and physical intervention parameters (MWA and US irradiation) remained identical to the protocols above.

For mouse bilateral tumor model, H22 cells (1×10⁶ in 100 μL PBS) were inoculated into the contralateral flank on day -5. The tumor volume and mouse body were recorded every other day. On day 12, the mice were euthanized, and samples including primary and contralateral tumor tissues along with spleens were harvested. Single-cell suspensions derived from primary tumors were subjected to flow cytometric analysis to evaluate the infiltration levels of CD3⁺CD4⁺ T lymphocytes, CD3⁺CD8⁺ T lymphocytes, CD3⁺CD8⁺IFN-γ⁺ and CD3⁺CD8⁺Granzyme B⁺ cytotoxic T lymphocytes (CTLs; anti-CD3-PE, anti-CD8α-APC, anti-IFN-γ-FITC, and anti-Granzyme B-PE-Cy7 antibodies). Similarly, contralateral tumor suspensions were assessed for the prevalence of CD3⁺CD4⁺ and CD3⁺CD8⁺ T lymphocytes, CD11b⁺CD86⁺ M1 TAMs and CD11b⁺CD206⁺ M2 TAMs, CD11b⁺Ly6G⁺ PMN MDSCs and CD11b⁺Ly6C⁺ M MDSCs, CD3⁺CD8⁺IFN-γ⁺ and CD3⁺CD8⁺Granzyme B⁺ CTLs. Splenic suspensions were analyzed for central memory T cells (T_CM_) and effector memory T cells (T_EM_) via staining with anti-CD3-PE, anti-CD8-APC, anti-CD44-PE-Cy7, and anti-CD62L-FITC. Furthermore, CD4 and CD8 dual immunofluorescence staining of contralateral tumor tissues was performed. Concurrently, the concentrations of TNF-α and IFN-γ in contralateral tumor tissues were quantified using ELISA.

For mouse lung metastasis model, on day 7 after the synergistic therapeutic intervention, H22 cells were intravenously injected via the tail vein to induce pulmonary metastases. On day 18, the mice were sacrificed, and lung tissues were excised for analysis. Lung weights were documented, and subsequent histological examinations included H&E and Ki67 IHC staining to assess necrosis and proliferative activity of pulmonary metastases, respectively.

***12.6.*** ***Orthotopic liver cancer suppression***

To evaluate therapeutic efficacy of TCPD nanodepots combined with MWA and US irradiation in synergy with anti-PD-1 for orthotopic liver cancer suppression, H22 cells (3 × 10⁶ cells in 20 μL PBS) were surgically inoculated into the left lower hepatic lobe of BALB/c mice via direct subcapsular injection. Seven days post-inoculation, tumor-bearing mice were randomly assigned to four treatment groups (n = 6 per group) and received interventions as described in **Section 12.5**. For G3 and G4, intratumoral TCPD nanodepot injection, MWA and US irradiation were performed under direct visualization through a 1.5 cm midline abdominal incision. Specifically, the MW antenna was inserted perpendicularly into the tumor center to a depth of approximately 3 mm, while US was delivered via a planar transducer coupled to the exposed liver surface with sterile acoustic gel. Both physical stimulations were administered one day post-TCPD nanodepot injection to permit nanodepot distribution. Real-time infrared thermography monitored intratumoral and adjacent normal liver tissue temperatures throughout MWA. On Day 15 post-treatment, mice were euthanized for weight measurement of liver/body and histological evaluation via H&E staining and CD4/CD8 dual immunofluorescence staining to assess tumor necrosis and immune infiltration. Parallel cohorts (n = 6 per group) were monitored for survival up to day 40.

***13. Statistical*** ***analysis***

All results were indicated as the mean ± standard deviation. Individual data represent biologically independent experiments, individual animals, or technical replicates. Student’s t-test was used for two-group comparisons and one-way analysis of variance (ANOVA) with Tukey’s multiple comparisons test was utilized for multiple comparisons. For longitudinal tumor volume data, statistical analysis was performed using a Restricted Maximum Likelihood (REML)-based linear mixed-effects model with Geisser-Greenhouse correction to evaluate the main effects of treatment, time, and their interaction, followed by Tukey’s multiple comparisons test. Survival analysis was assessed using the Kaplan-Meier method with log-rank test. All statistical analyses were carried out with GraphPad Prism 8.0. (N.S., not significant; **P* < 0.05; ***P* < 0.01; ****P* < 0.001).

**Supplementary Figures**

**Fig. S1** (a) Representative high-resolution transmission electron microscopy (HRTEM) and EDS elements mapping images of Cs-PB. (b) Hydrodynamic size and Zeta potential analyses of Cs-PB. (c) EDS elements analysis of Cs-PB. (d-e) Full-pattern Rietveld refinement of powder X-ray diffraction pattern and three-dimensional reconstruction of Cs-PB. (f) Raman spectra of Cs-PB and Telaglenastat. (g) Enlarge Raman spectra of Cs-PB. (h) X-ray photoelectron spectroscopy of Cs-PB and Telaglenastat. (i) Cumulative *in vitro* release profiles of Cs⁺ from Cs-PB (20 μg/mL) in buffers at pH 7.4 and pH 6.5. (j) Cell viability assessed by CCK-8 assays following incubation with different concentrations of CsCl. Data in (i) and (j) represent n=3 biologically independent experiments and are presented as mean ± standard deviation. Scale bar: 5, 10, and 50 nm.

**Fig. S2** (a) Absorbance changes of the TMB probe under different experimental conditions. (b-c) Infrared thermographic images and corresponding temperature-time profiles under various interventions. Data in (c) represent n=3 biologically independent experiments and are presented as mean ± standard deviation.

**Fig. S3** Gelation processes of HD hydrogel and TCPD nanodepot.

**Fig. S4** (a) Loading content (LC) of PD, CPD, and TCPD nanodepots. (b) Encapsulation efficiency (EE) of PD, CPD, and TCPD nanodepots. Data represent n=3 independent technical replicates and are presented as mean ± standard deviation.

**Fig. S5** Raman spectra of nanodepots.

**Fig. S6** Gelation time of different nanodepots.

**Fig. S7** Injectable performance of TCPD nanodepots with 23G and 26G syringes.

**Fig. S8** Thermogravimetric analysis.

**Fig. S9** (a) Swelling behaviors of different nanodepots. (b) Hemolysis experiments analysis. NC: saline; PC: deionized water. Data in (a) represent n=3 independent technical replicates and in (b) represent n=6 biologically independent experiments, and are presented as mean ± standard deviation.

**Fig. S10** (a) UV absorption of TMB probe, H₂O₂ alone, TCPD extract alone, and TMB probe incubated with TCPD extract. (b) Absorbance changes of the TMB probe under various interventions. (c) Concentration-dependent UV absorption of TMB probe treated with TCPD nanodepots.

**Fig. S11** (a) UV absorption of MB probe, H₂O₂ alone, TCPD extract alone, and MB probe incubated with TCPD extract. (b) UV absorption of MB probe treated with different interventions. (c) Concentration-dependent UV absorption of MB probe treated with TCPD nanodepots. (d) Time-dependent UV absorption of MB probe treated with TCPD nanodepots (20 mg/mL).

**Fig. S12** (a) UV absorption of DPBF probe, H₂O₂ alone, TCPD extract alone, and DPBF probe incubated with TCPD extract. (b) UV absorption of DPBF probe treated with different interventions. (c) Concentration-dependent UV absorption of DPBF probe treated with TCPD nanodepots. (d) Time-dependent UV absorption of DPBF probe treated with TCPD nanodepots (20 mg/mL).

**Fig. S13** (a) UV absorption of DTNB probe, DTNB probe incubated with GSH, H₂O₂ alone, TCPD extract alone, and DTNB probe incubated with GSH and TCPD extract. (b) UV absorption of DTNB probe treated with different interventions. (c) Concentration-dependent UV absorption of DTNB probe treated with TCPD nanodepots.

**Fig. S14** (a) Concentration-dependent thermal images. (b) Concentration-dependent MW intensity-dependent temperature curves treated with TCPD nanodepots (20 mg/mL). (c) MW intensity-dependent temperature curves treated with TCPD nanodepots. Data are presented as mean ± standard deviation.

**Fig. S15** Cell Counting Kit-8 assays for H22 cells viability treated with different interventions. Data represent n=6 biologically independent experiments and are presented as mean ± standard deviation. Statistical comparisons were performed using one-way ANOVA with Tukey’s multiple comparisons test. ∗*P*<0.05, ∗∗*P*<0.01, ∗∗∗*P*<0.001, ∗∗∗∗*P*<0.0001; N.S., not significant.

**Fig. S16** (a) Statistical analysis of relative intracellular (IC) ATP content. (b) Statistical analysis of relative GSH mean fluorescence intensity (MFI). (c) Statistical analysis of relative cell migration. Data represent n=3 biologically independent experiments and are presented as mean ± standard deviation. Statistical comparisons were performed using one-way ANOVA with Tukey’s multiple comparisons test.

**Fig. S17** (a) Gene Ontology (GO) Biological Process (BP) and (b) Kyoto Encyclopedia of Genes and Genomes (KEGG) enrichment analysis of differentially downregulated genes (DEGs) in the RNA-seq. (c-f) Gene Set Enrichment Analysis (GSEA) enrichment analyses of immune-related upregulated and cellular-related downregulated DEGs.

**Fig. S18** (a-j) Statistical analyses of relative Caspase 1, c-Caspase 1, GSDMD, GSDMD-N, Caspase 3, c-Caspase 3, RIPK3, p-RIPK3, MLKL, and p-MLKL proteins expression levels. Data represent n=3 biologically independent experiments and are presented as mean ± standard deviation. Statistical comparisons were performed using one-way ANOVA with Tukey’s multiple comparisons test.

**Fig. S19** (a) Statistical analysis of lactate dehydrogenase (LDH) release. (b) Quantification of IL-β and IL-18 concentration in supernatant from H22 cells by ELISA kits. Data represent n=3 biologically independent experiments and are presented as mean ± standard deviation. Statistical comparisons were performed using one-way ANOVA with Tukey’s multiple comparisons test.

**Fig. S20** Statistical analysis of H22 cells apoptosis. Data are presented as mean ± standard deviation. Statistical comparisons were performed using one-way ANOVA with Tukey’s multiple comparisons test.

**Fig. S21** Representative images and statistical analysis of YO-PRO-1/PI dual staining. Scale bar: 100 μm. Data represent n=3 biologically independent experiments and are presented as mean ± standard deviation. Statistical comparisons were performed using one-way ANOVA with Tukey’s multiple comparisons test. Scale bar: 100 μm.

**Fig. S22** (a-d) Representative images and statistical analysis of GSDMD-N, cleaved Caspase 3, and p-MLKL immunofluorescence staining in H22 cells. Scale bar: 20 μm. Data represent n=3 biologically independent experiments and are presented as mean ± standard deviation. Statistical comparisons were performed using one-way ANOVA with Tukey’s multiple comparisons test. Scale bar: 20 μm.

**Fig. S23** Cell Counting Kit-8 assays for H22 cells viability treated with different interventions. Data represent n=6 biologically independent experiments and are presented as mean ± standard deviation. Statistical comparisons were performed using one-way ANOVA with Tukey’s multiple comparisons test.

**Fig. S24** (a-c) Statistical analyses of relative mito-SOX, JC-1 monomers/aggregates, and 8-oxo-dG MFI. Data represent n=3 biologically independent experiments and are presented as mean ± standard deviation. Statistical comparisons were performed using one-way ANOVA with Tukey’s multiple comparisons test.

**Fig. S25** (a-j) Statistical analyses of relative Bcl-2, BAX, L-OPA1, Cytochrome C (Cyt C), p-PERK, p-eIF2α, ATF4, and CHOP proteins expression levels. Data represent n=3 biologically independent experiments and are presented as mean ± standard deviation. Statistical comparisons were performed using one-way ANOVA with Tukey’s multiple comparisons test.

**Fig. S26** (a-b) Representative images and statistical analysis of PAR immunofluorescence staining in H22 cells. Data represent n=3 biologically independent experiments and are presented as mean ± standard deviation. Statistical comparisons were performed using one-way ANOVA with Tukey’s multiple comparisons test. Scale bar: 20 μm.

**Fig. S27** (a-e) Statistical analyses of relative NLRP3, NLRC5, AIM2, and ZBP1 proteins expression levels. Data represent n=3 biologically independent experiments and are presented as mean ± standard deviation. Statistical comparisons were performed using one-way ANOVA with Tukey’s multiple comparisons test.

**Fig. S28** Co-immunoprecipitation in H22 cells with IgG control antibodies or anti-ASC antibodies after TCPD nanodepots combined with US and MW irradiation. G1: Control; G6: TCPD+US+MW.

**Fig. S29** (a-g) Western blotting and statistical analyses for PANoptosis executioner proteins in ASC-silenced H22 cells treated with TCPD-empowered sono-thermal ablation. Data represent n=3 biologically independent experiments and are presented as mean ± standard deviation. Statistical comparisons were performed using one-way ANOVA with Tukey’s multiple comparisons test.

**Fig. S30** (a-b) Representative images and of statistical analysis mito-SOX fluorescence staining. (c-i) Western blotting and statistical analyses for PANoptosis executioner proteins. (j) Cell Counting Kit-8 assays for H22 cells viability. Data represent n=3 biologically independent experiments and are presented as mean ± standard deviation. Statistical comparisons were performed using one-way ANOVA with Tukey’s multiple comparisons test. Scale bar: 100 μm.

**Fig. S31** (a-d) Statistical analyses of relative STING, p-STING, IRF3, and p-IRF3 proteins expression levels. Data represent n=3 biologically independent experiments and are presented as mean ± standard deviation. Statistical comparisons were performed using one-way ANOVA with Tukey’s multiple comparisons test.

**Fig. S32** (a-c) Statistical analyses of relative CRT and HMGB1 MFI. Data represent n=3 biologically independent experiments and are presented as mean ± standard deviation. Statistical comparisons were performed using one-way ANOVA with Tukey’s multiple comparisons test.

**Fig. S33** (a-b) Quantification of TNF-α and IFN-γ concentration in supernatant from BMDCs by ELISA kits. Data represent n=3 biologically independent experiments and are presented as mean ± standard deviation. Statistical comparisons were performed using one-way ANOVA with Tukey’s multiple comparisons test.

**Fig. S34** (a) Representative images and statistical analysis of iNOS immunofluorescence staining in RAW264.7 cells. Data represent n=3 biologically independent experiments and are presented as mean ± standard deviation. Statistical comparisons were performed using one-way ANOVA with Tukey’s multiple comparisons test. Scale bar: 100 μm.

**Fig. S35** (a) Time-temperature profiles of peritumoral and intratumoral tissues. (b) Representative H&E staining images of peritumoral tissues. Data represent n=3 biologically independent experiments and are presented as mean ± standard deviation. Scale bar: 100 μm and 1 mm.

**Fig. S36** (a-b) Body weight and survival analysis curves of mice during treatments. (c) Statistical analysis of GLS1 immunohistochemistry staining in tumor tissues. Data in (a), (b) represent n=3 independent biologically mice per group, and (c) represent n=3 biologically independent experiments, and are presented as mean ± standard deviation. Statistical comparisons were performed using one-way ANOVA with Tukey’s multiple comparisons test.

**Fig. S37** Representative H&E staining images of major organs including heart, liver, spleen, lung, and kidney. Scale bar: 50 μm. G1: Control; G2: TCPD; G3: CPD+US+MW; G4: TCPD+MW; G5: TCPD+US; G6: TCPD+US+MW.

**Fig. S38** (a) Representative images from small animal imaging systems used to track degradation of TCPD nanodepots *in vivo*. (b) Representative H&E staining images of TCPD nanodepots and surrounding tissues. (c-d) Changes in serum IL-6 and TNF-α levels following TCPD nanodepots implantation. Data represent n=3 biologically independent experiments and are presented as mean ± standard deviation. Scale bar: 50 μm.

**Fig. S39** (a) Time-dependent residual contents of Cs and Fe from TCPD at the subcutaneous injection site over 28 days. (b-c) *In vivo* biodistribution kinetics of Cs and Fe in blood and major organs (heart, liver, spleen, lung, kidney, and muscle) at predetermined time points. (d-e) Cumulative excretion profiles of Cs and Fe in urine and feces over 28 days. Data represent n=3 biologically independent experiments and are presented as mean ± standard deviation.

**Fig. S40** (a) Comprehensive hematological evaluations indicating systemic immune compatibility over 28 days. (b) Serum biochemical analysis of hepatic and renal function markers over 28 days. Data are presented as mean ± standard deviation.

**Fig. S41** (a-b) Representative images and statistical analysis of CRT immunofluorescence staining in tumor tissues. Data represent n=3 biologically independent experiments and are presented as mean ± standard deviation. Statistical comparisons were performed using one-way ANOVA with Tukey’s multiple comparisons test. Scale bar: 50 μm.

**Fig. S42** (a) Statistical analysis of CD45^+^CD3^+^ T cells in tumor tissues. (b-c) Statistical analyses of CD4/CD8 dual immunofluorescence staining. Data represent n=3 biologically independent experiments and are presented as mean ± standard deviation. Statistical comparisons were performed using one-way ANOVA with Tukey’s multiple comparisons test.

**Fig. S43** (a-d) Statistical analyses of CD3^+^CD4^+^ and CD3^+^CD8^+^ T cells, CD3^+^CD8^+^IFN-γ^+^ cytotoxic T lymphocytes (CTLs; gated on CD3^+^CD8^+^), and CD3^+^CD8^+^ Granzyme B^+^ (Gra B) CTLs (gated on CD3^+^CD8^+^) in contralateral tumor tissues. (e-f) Statistical analyses of CD3^+^CD8^+^CD44^+^CD62L^+^ central memory T cells (T_CM_) and CD3^+^CD8^+^CD44^+^CD62L^-^ effector memory T cells (T_EM_) in spleens. Data represent n=3 biologically independent experiments and are presented as mean ± standard deviation. Statistical comparisons were performed using one-way ANOVA with Tukey’s multiple comparisons test.

**Fig. S44** (a) Flow cytometry images and statistical analyses of CD3^+^CD4^+^FOXP3^+^ regulatory T cells (Tregs; gated on CD3^+^CD4^+^), CD86^+^ and CD206^+^ tumor-associated macrophages (TAMs; gated on CD45^+^F4/80^+^), and Ly6G^+^ polymorphonuclear (PMN) and Ly6C^+^ mononuclear myeloid-derived suppressor cells (PMN and M MDSCs; gated on CD45^+^) in contralateral tumor tissues. Data represent n=3 biologically independent experiments and are presented as mean ± standard deviation. Statistical comparisons were performed using one-way ANOVA with Tukey’s multiple comparisons test.

**Fig. S45** Mouse lung metastasis model timeline flowchart.

**Supplementary tables**

**Table S1.** The sample codes and compositions

| **Sample code** | **W_HA-DA_ (wt%)** | **W_Tela_ (wt%)** | **W_Ce6_ (wt%)** | **W_Cs-PB_ (wt%)** | **H_2_O_2_ (M)** |
| --- | --- | --- | --- | --- | --- |
| Cs-PB@HD (PD) | 4 | 0 | 0 | 0.1 | 0.05 |
| Ce6/Cs-PB@HD (CPD) | 4 | 0 | 0.1 | 0.1 | 0.05 |
| Tela/Ce6/Cs-PB@HD (TCPD) | 4 | 0.5 | 0.1 | 0.1 | 0.05 |

W_HA-DA_, W_Tela_, W_Ce6_, and W_Cs-PB_ denote the mass of the HA-DA polymer, Tela powder, Ce6 powder, and Cs-PB nanoparticles, respectively, relative to the total mass of the aqueous HA-DA solution.

**Table S2**. List of colorimetric assay kits

| **Codes** | **Species** | **Supplier** | **Category** |
| --- | --- | --- | --- |
| Glutamine | Mouse | Elabscience Biotechnology, China | E-BC-K853-M |
| Glutamate | Mouse | Elabscience Biotechnology, China | E-BC-K903-M |
| Glutaminase | Mouse | Elabscience Biotechnology, China | E-BC-K660-M |
| Citrate | Mouse | Arigo Biolaboratories, China | ARG81388 |
| Isocitrate | Mouse | Arigo Biolaboratories, China | ARG82172 |
| α-ketoglutarate | Mouse | Arigo Biolaboratories, China | ARG82657 |
| Succinate | Mouse | Arigo Biolaboratories, China | ARG82193 |
| Fumarase | Mouse | Arigo Biolaboratories, China | ARG82158 |
| Succinate | Mouse | Arigo Biolaboratories, China | ARG82193 |
| Fumarase | Mouse | Arigo Biolaboratories, China | ARG82158 |
| Malate | Mouse | Arigo Biolaboratories, China | ARG82178 |
| Oxaloacetate | Mouse | Arigo Biolaboratories, China | ARG82187 |

**Table S3.** List of primary antibodies

| **Antigens** | **Host**/**category** | **Dilution (IF)** | **Dilution (IHC)** | **Dilution (WB)** | **Supplier** |
| --- | --- | --- | --- | --- | --- |
| Caspase 1 | Rabbit/polyclonal | - | - | 1:2000 | ProteinTech Group, China, Cat. 22915-1-AP |
| Cleave Caspase 1 | Rabbit/monoclonal | - | - | 1:1000 | Cell Signaling Technology, USA, Cat. 89332 |
| GSDMD | Rabbit/polyclonal | - | - | 1:5000 | ProteinTech Group, China, Cat. 20770-1-AP |
| GSDMD-N | Rabbit/monoclonal | 1:200 | - | 1:2000 | MedChemExpress, USA, Cat. HY-P86158 |
| Caspase 3 | Rabbit/monoclonal | - | - | 1:2000 | ProteinTech Group, China, Cat. 19677-1-AP |
| Cleave Caspase 3 | Rabbit/monoclonal | 1:100 | - | 1:2000 | ProteinTech Group, China, Cat. 25128-1-AP |
| RIPK3 | Mouse/monoclonal | - | - | 1:5000 | ProteinTech Group, China, Cat. 66675-1-Ig |
| p-RIPK3 | Mouse/monoclonal | - | - | 1:5000 | ProteinTech Group, China, Cat. 66675-1-Ig |
| MLKL | Mouse/monoclonal | - | - | 1:5000 | ProteinTech Group, China, Cat. 66675-1-Ig |
| p-MLKL | Rabbit/monoclonal | 1:1600 | - | 1:1000 | Cell Signaling Technology, USA, Cat. 37333 |
| Bcl-2 | Mouse/monoclonal | - | - | 1:10000 | ProteinTech Group, China, Cat. 68103-1-Ig |
| BAX | Rabbit/monoclonal | - | - | 1:20000 | ProteinTech Group, China, Cat. 50599-2-Ig |
| OPA1 | Rabbit/monoclonal | - | - | 1:10000 | ProteinTech Group, China, Cat. 27733-1-AP |
| Cytochrome C | Rabbit/polyclonal | - | - | 1:5000 | ProteinTech Group, China, Cat. 10993-1-AP |
| 8-Oxo-dG | Mouse/monoclonal | - | - | 1:10000 | Abcam Ltd, USA, Cat. ab62623 |
| Poly-ADP Ribose | Rabbit/monoclonal | - | - | 1:1000 | Cell Signaling Technology, USA, Cat. 89190 |
| p-PERK | Rabbit/polyclonal | - | - | 1:2000 | ProteinTech Group, China, Cat. 82534-1-RR |
| p-eIF2α | Mouse/monoclonal | - | - | 1:5000 | ProteinTech Group, China, Cat. 68023-1-Ig |
| ATF4 | Rabbit/polyclonal | - | - | 1:1000 | ProteinTech Group, China, Cat. 10835-1-AP |
| CHOP | Rabbit/polyclonal | - | - | 1:3000 | ProteinTech Group, China, Cat. 15204-1-AP |
| NLRP3 | Rabbit/polyclonal | - | - | 1:5000 | ProteinTech Group, China, Cat. 85967-1-RR |
| NLRC5 | Rabbit/polyclonal | - | - | 1:1000 | Abcam Ltd, UK, Cat. ab105411 |
| ASC | Rabbit/polyclonal | - | - | 1:5000 | ProteinTech Group,  China, Cat. 30641-1-AP |
| AIM2 | Mouse/monoclonal | - | - | 1:500 | ProteinTech Group, China, Cat. CL488-66902 |
| ZBP1 | Rabbit/polyclonal | - | - | 1:1000 | Thermo Fisher Scientific, USA, Cat. PA5-20455 |
| Pyrin | Rabbit/polyclonal | - | - | 1:2000 | ProteinTech Group, China, Cat. 24280-1-AP |
| Caspase 8 | Rabbit/polyclonal | - | - | 1:2000 | ProteinTech Group, China, Cat. 13423-1-AP |
| RIPK1 | Rabbit/polyclonal | - | - | 1:2000 | ProteinTech Group, China, Cat. 34062-1-AP |
| Cleave Caspase 7 | Rabbit/monoclonal | - | - | 1:1000 | Abcam Ltd, UK, Cat. ab256469 |
| STING | Rabbit/polyclonal | - | - | 1:1000 | Abcam Ltd, UK, Cat. ab288157 |
| p-STING | Rabbit/polyclonal | - | - | 1:1000 | Thermo Fisher Scientific, USA, Cat. PA5-105674 |
| IRF3 | Rabbit/polyclonal | - | - | 1:5000 | ProteinTech Group, China, Cat. 11312-1-AP |
| p-IRF3 | Rabbit/monoclonal | - | - | 1:1000 | Thermo Fisher Scientific, USA, Cat. MA5-57852 |
| CRT | Rat/monoclonal | 1:200 | - | - | ProteinTech Group, China, Cat. 10292-1-AP |
| HMGB1 | Rabbit/polyclonal | 1:1000 | - | - | Servicebio Technology, China, Cat. GB11103-100 |
| MHC-II | Rat/monoclonal | 1:200 | - | - | Thermo Fisher Scientific, USA, Cat. 14-0920-82 |
| iNOS | Rat/monoclonal | 1:500 | - | - | Thermo Fisher Scientific, USA, Cat. 53-5920-82 |
| Ki67 | Rat/monoclonal | - | 1:600 | - | Thermo Fisher Scientific, USA, Cat. 14-5698-82 |
| Granzyme B | Rabbit/polyclonal | 1:200 | - | - | ProteinTech Group, China, Cat. 13588-1-AP |
| CD4 | Rabbit/monoclonal | 1:200 | - | - | Servicebio Technology, China, Cat. GB15064-100 |
| CD8 | Rabbit/monoclonal | 1:400 | - | - | Servicebio Technology, China, Cat. GB15068-100 |
| CD86 | Rat/monoclonal | 1:300 | - | - | Thermo Fisher Scientific, USA, Cat. MA1-10299 |
| β-Actin | Rabbit/polyclonal | 1:500 | - | 1:4000 | ProteinTech Group, China, Cat. 20536-1-AP |
| β-Tubulin | Rabbit/polyclonal | - | - | 1:2000 | ProteinTech Group, China, Cat. 10094-1-AP |

**Table S4.** List of secondary antibodies and fluorescent antibodies

| **Secondary detection** | **Host** | **Method** | **Dilution** | **Supplier** |
| --- | --- | --- | --- | --- |
| Anti-Mouse-IgG (H+L)-Alexa Fluor 488 | Goat | IF | 1:300 | Servicebio Technology, China, Cat. GB22301 |
| Anti- Rabbit-IgG (H+L)-Alexa Fluor 488 | Goat | IF | 1:300 | Servicebio Technology, China, Cat. GB25303 |
| Anti-Rabbit-IgG (H+L)-Alexa Fluor 594 | Goat | IF | 1:300 | Servicebio Technology, China, Cat. GB28301 |
| Anti-Rabbit-IgG (H+L)-FITC | Goat | IF | 1:300 | Servicebio Technology, China, Cat. GB22303 |
| Anti-Rabbit-IgG (H+L)-Cy3 | Goat | IF | 1:300 | Servicebio Technology, China, Cat. GB21303 |
| Anti-Rat-IgG (H+L)-Cy3 | Goat | IF | 1:300 | Servicebio Technology, China, Cat. GB21302 |
| Anti-Mouse-IgG (H+L)-Cy3 | Goat | IF | 1:300 | Servicebio Technology, China, Cat. GB21301 |
| Anti-Mouse-IgG (H+L)-HRP | Goat | IHC | 1:500 | Servicebio Technology, China, Cat. GB23301 |
| Anti-Rat-IgG (H+L)-HRP | Goat | IHC | 1:500 | Servicebio Technology, China, Cat. GB23302 |
| Anti-Rabbit-IgG (H+L)-HRP | Goat | IHC | 1:500 | Servicebio Technology, China, Cat. GB23303 |

**Table S5**. List of flow cytometer antibodies

| **Antigens** | **Host** | **Dilution** | **Supplier** |
| --- | --- | --- | --- |
| Anti-CD11b-FITC | Rat | 1:25 | Thermo Fisher Scientific, USA,  Cat. 11-0112-82 |
| Anti-CD11b-APC | Rat | 1:25 | Thermo Fisher Scientific, USA,  Cat. 17-0112-83 |
| Anti-CD11c-APC | Armenian hamster | 1:25 | Thermo Fisher Scientific, USA,  Cat. 17-0114-82 |
| Anti-CD80-PE | Armenian hamster | 1:25 | Thermo Fisher Scientific, USA,  Cat. 12-0801-82 |
| Anti-CD86-FITC | Armenian hamster | 1:25 | Thermo Fisher Scientific, USA,  Cat. 11-0862-82 |
| Anti-CD206-PE | Rat | 1:25 | Thermo Fisher Scientific, USA,  Cat. 12-2061-82 |
| Anti-F4/80-APC | Rat | 1:25 | Thermo Fisher Scientific, USA,  Cat. 17-4801-82 |
| Anti-CD3-PE | Rat | 1:25 | Thermo Fisher Scientific, USA,  Cat. 12-0031-82 |
| Anti-CD4-FITC | Rat | 1:25 | Thermo Fisher Scientific, USA,  Cat. 11-0041-82 |
| Anti-CD8a-APC | Rat | 1:25 | Thermo Fisher Scientific, USA,  Cat. 17-0081-82 |
| Anti-FOXP3-APC | Rat | 1:25 | Thermo Fisher Scientific, USA,  Cat. 17-5773-82 |
| Anti-Gr-1-APC | Rat | 1:25 | Thermo Fisher Scientific, USA,  Cat. 17-5931-82 |
| Anti-Ly6C-APC | Rat | 1:25 | Thermo Fisher Scientific, USA,  Cat. 17-5932-82 |
| Anti-Ly6G-PE | Rat | 1:25 | Thermo Fisher Scientific, USA,  Cat. 12-9668-82 |
| Anti-IFN-γ-FITC | Rat | 1:25 | Thermo Fisher Scientific, USA,  Cat. 11-7311-82 |
| Anti- Granzyme B-PE-Cyanine 7 | Rat | 1:25 | Thermo Fisher Scientific, USA,  Cat. 25-8898-82 |
| Anti-CD44-PE-Cyanine 7 | Rat | 1:25 | Thermo Fisher Scientific, USA,  Cat. 25-0441-82 |
| Anti-CD45-Brilliant Violet 421 | Rat | 1:25 | Thermo Fisher Scientific, USA,  Cat. 404-0451-82 |
| Anti-CD62L-FITC | Rat | 1:25 | Thermo Fisher Scientific, USA,  Cat. 11-0621-82 |

**Table S6**. List of enzyme-linked immunosorbent assay kits

| **Codes** | **Species** | **Supplier** | **Category** |
| --- | --- | --- | --- |
| IL-1β | Mouse | Servicebio Technology, China | GEM0001-96T |
| IL-6 | Mouse | Servicebio Technology, China | GEM0001-96T |
| IL-10 | Mouse | Servicebio Technology, China | GEM0003-96T |
| IL-12 | Mouse | Elabscience Biotechnology, China | E-EL-M3062 |
| IL-18 | Mouse | Elabscience Biotechnology, China | E-EL-M0730 |
| TNF-α | Mouse | Elabscience Biotechnology, China | E-EL-M3063 |
| IFN-γ | Mouse | Elabscience Biotechnology, China | E-EL-M0048 |
| IFN-β | Mouse | Elabscience Biotechnology, China | E-EL-M0033 |
| TGF-β | Mouse | Elabscience Biotechnology, China | E-EL-0162 |

**Reference**

[1]	a) Q. Wang, Y. Wen, B. Bi, K. Li, Y. Liu, B. Li, S. Zhou, Z. Li, J. Xu, M. Qiu, Y. Li, M. Wu, Y. Chen, W. Wu, J. Zhao, *Sci Bull (Beijing)* **2025**, *70* (11), 1804, <https://doi.org/10.1016/j.scib.2025.03.015>; b) J. E, Y. Zhao, X. Zhang, Q. Zhang, Y. Xu, S. Liu, H. Ding, J. Zhu, P. Yang, R. Xie, *Sci Adv* **2026**, *12* (25), eaea6844, <https://doi.org/10.1126/sciadv.aea6844>; c) C. Zhang, B. Wang, Y. Wang, H. Yu, H. Liu, W. Ma, W. Lou, F. Xu, L. Xu, X. Zhang, L. Xie, X. Lu, Q. Zhao, T. Jiang, *Adv Mater* **2026**, *38* (19), e20546, <https://doi.org/10.1002/adma.202520546>.

[2]	a) Z. Yao, C. Qi, F. Zhang, H. Yao, C. Wang, X. Cao, C. Zhao, Z. Wang, M. Qi, C. Yao, X. Wang, H. Xia, *Acta Biomater* **2024**, *173*, 365, <https://doi.org/10.1016/j.actbio.2023.10.024>; b) L. Qiao, G. Zhu, T. Jiang, Y. Qian, Q. Sun, G. Zhao, H. Gao, C. Li, *Adv Mater* **2024**, *36* (8), e2308241, <https://doi.org/10.1002/adma.202308241>.

[3]	a) B. Sun, J. Liu, X. Liu, J. Li, G. Zhang, T. Sun, C. Zheng, X. Kan, *Cell Death Dis* **2025**, *16* (1), 288, <https://doi.org/10.1038/s41419-025-07622-z>; b) M. P. Mulligan, M. W. Boudreau, B. A. Bouwens, Y. Lee, H. W. Carrell, J. Zhu, S. Mousses, D. J. Shapiro, E. R. Nelson, T. M. Fan, P. J. Hergenrother, *ACS Cent Sci* **2025**, *11* (2), 228, <https://doi.org/10.1021/acscentsci.4c01628>; c) K. Heckelsmiller, S. Beck, K. Rall, B. Sipos, A. Schlamp, E. Tuma, S. Rothenfusser, S. Endres, G. Hartmann, *Eur J Immunol* **2002**, *32* (11), 3235, <https://doi.org/10.1002/1521-4141(200211)32:11><3235::Aid-immu3235>3.0.Co;2-j.
